# Downregulation of Engulfment and cell motility 1 (Elmo1) induces quiescence and resistance to poly(I:C)-induced apoptosis in endothelial cells

**DOI:** 10.1038/s41419-025-08341-1

**Published:** 2025-12-20

**Authors:** Yukako Kayashima, Anshulika A. Deshmukh, Yuki Kiyokawa, Niroshani M. W. Wariyapperuma Appuhamillage, Jiayi Zhou, Mohamed-Yahia S. Monawar, Melanie Nassar-Guifarro, Feng Li, Nobuyo Maeda

**Affiliations:** Department of Pathology and Laboratory Medicine, University of North Carolina at Chapel Hill, Chapel Hill, NC USA

**Keywords:** Apoptosis, Cell division

## Abstract

Severe viral infections can cause cell death as a protective mechanism to eliminate defective cells and limit further viral propagation. However, the precise mechanism underlying the decision between cell survival and death remains unclear. Here, we demonstrate that Engulfment and Cell Motility 1 (ELMO1), an intracellular protein that facilitates cytoskeleton rearrangement through activation of Rac family small GTPase 1 (RAC1), is involved in the Toll-like receptor 3 (TLR3)-induced apoptosis in human umbilical vein endothelial cells. RNA sequencing of cells treated with ELMO1-targeting siRNA (siELMO1) revealed that knockdown of ELMO1 increased the transcripts of extracellular matrix genes including *COL5A1* and decreased the expression of cell cycle and DNA replication-related genes such as *CCND1* and *CDK1*. These siELMO1 treated cells also showed G1/S cell cycle arrest. When stimulated with polyinosinic-polycytidylic acid (poly(I:C)), a TLR3 agonist, inflammatory cytokines and chemokines such as *CXCL8* and *IL6* robustly increased in Mock-treated cells, while siNT (non-targeting) cells underwent massive cell death and showed reduced inflammatory responses, reflecting apoptosis-inducing effects of sequential TLR3 activation by siRNA and poly(I:C). Strikingly, siELMO1 cells were resistant to the cell death, and restored inflammatory cytokine responses to the same level as Mock-treated cells. Mechanistically, apoptosis in siNT increased through the activation of caspase-8, whereas downregulation of ELMO1 inhibited the cleavage of caspase-8. These results indicate that ELMO1 is involved in the regulation between survival or death in endothelial cells through the regulation of caspase-8 activity downstream of TLR3, suggesting the importance of the ELMO1 in cell proliferation as well as susceptibility to poly(I:C)-induced apoptosis.

## Introduction

Engulfment and Cell Motility 1 (ELMO1) is an intracellular molecule that facilitates the activity of Rac family small GTPase 1 (RAC1) through interaction with the Dedicator of Cytokinesis (DOCK) family of guanine nucleotide exchange factors (GEFs) [1, 2]. In response to various stimuli such as activation of an engulfment receptor Brain Angiogenesis Inhibitor 1 (BAI1) [3], ELMO1 forms a complex with DOCK and RAC1, enhancing the function of DOCK that facilitates transition of RAC1 from a GDP-bound inactive form to GTP-bound active form [4, 5]. The activated RAC1 then modulates several diverse cellular functions such as phagocytosis and cell migration through cytoskeletal rearrangement, as well as production of reactive oxygen species (ROS) as a component of NADPH oxidase (NOX) [6].

Experiments using animal models showed Elmo1 plays a critical role in acute and chronic inflammatory diseases via multiple cellular mechanisms, including bacterial infection [7, 8], rheumatoid arthritis, ankylosing spondylitis and peritonitis [9–11] by enhancing bacterial invasion or by accelerating chemotaxis of immune cells. In addition, Elmo1 modifies the severity of diabetic nephropathy and cardiomyopathy through production of ROS [12, 13]. These studies indicate that suppression of ELMO1 is likely protective in various inflammatory diseases.

Although ELMO1 is highly expressed in endothelial cells (proteinatlas.org) [14], and these cells are known to play a critical role in inflammatory diseases [15, 16], little research on ELMO1 has focused on inflammatory endothelial cells. During inflammation, endothelial cells are activated by pathogen-associated molecular patterns (PAMPs) and damage-associated molecular patterns (DAMPs), as well as inflammatory cytokines and chemokines [17]. The activated endothelial cells in turn produce interferons, inflammatory cytokines and chemokines to induce anti-pathogenic responses and to attract immune cells such as neutrophils and monocytes [16]. Expression of cell surface adhesion molecules is also upregulated, accelerating attachment of immune cells in circulation [16].

Toll-like receptor 3 (TLR3) is a pattern recognition receptor that recognizes double-stranded RNA (dsRNA) produced from viruses and initiates innate immune responses, including production of Type I interferons, inflammatory cytokines, and chemokines [18]. In severe and persistent infection, activation of TLR3 can lead to cell death, which is a beneficial mechanism to the body that eliminates defective cells and restricts further viral propagation [19–21]. The switching mechanism between survival or death has been extensively studied in the context of death receptor signaling such as tumor necrosis factor receptor 1 (TNFR1) [22]. Upon stimulation of TNFR1, receptor-interacting serine/threonine kinase 1 (RIPK1) is recruited to the adapter protein tumor necrosis factor receptor associated death domain protein (TRADD) and ubiquitylated by cellular inhibitor of apoptosis proteins 1/2 (cIAP1/2). Ubiquitylated RIPK1 activates NF-kB, leading to the induction of pro-survival and pro-inflammatory genes [23–25]. Deubiquitylation of RIPK1 promotes the formation of a death-inducing complex composed of RIPK1, caspase-8, Fas-associated protein with death domain (FADD), and cellular FLICE-inhibitory protein (cFLIP_L_) [23–25]. FADD functions as an adapter that activates capase-8, and homodimerization and autoproteolysis of caspase-8 induce apoptosis through downstream effector caspases, including caspase-3 and caspase-7. The activity of caspase-8 is also modulated by cFLIP, a catalytically inactive homolog of caspase-8, through the formation of cFLIP-caspase-8 heterodimers [26]. Similar mechanisms likely exist under TLR activation [27, 28]. TLR3 signals through an adapter protein TIR-domain-containing adapter molecule 1 (TICAM1/TRIF), which interacts with RIPK1 and activates NF-kB, causing pro-survival and pro-inflammatory gene induction [29]. Stimulation of TLR3 also drives caspase-8 activation through assembly of ripoptosome, which includes caspase-8, RIPK1, FADD, and cFLIP [30], ultimately leading to apoptosis [24, 31, 32]. Lastly, caspase-8 can exert dual roles: the non-enzymatic role as a scaffold protein to enhance inflammatory responses [33–35], and the enzymatic role as an inducer of apoptosis and necroptosis [36–38].

In this study, we examined the effect of ELMO1 downregulation in EA.hy926 human umbilical vein endothelial cells (HUVECs) and found that knockdown of ELMO1 induces cell quiescence. Moreover, ELMO1 knockdown inhibited TLR3-induced cell death by blocking caspase-8 activation, while simultaneously restoring inflammatory responses. Our results suggest that ELMO1 is a modulator of the switching mechanism between survival or death in endothelial cells under inflammation.

## Materials and methods

### Cell culture and reagents

EA.hy926, a human umbilical vein endothelial cell (HUVEC) line originally immortalized by fusion with an A549 human lung epithelial line [39], is a kind gift from Dr. Cora-Jean S. Edgel at UNC Chapel Hill. Cells were maintained in Dulbecco’s modified eagle medium (DMEM) (Thermo Fisher Scientific, Waltham, MA, USA) supplemented with 10% fetal bovine serum (FBS) (MilliporeSigma, Burlington, MA, USA) and 100 U/ml penicillin/100 μg/mL streptomycin (Thermo Fisher Scientific) in 5% CO_2_ at 37 °C. The absence of mycoplasma contamination was confirmed using the Mycoplasma PCR Detection Kit (Abcam, Cambridge, MA, USA), Caspase-8 inhibitor Z‑IETD‑FMK was purchased from R&D systems (Minneapolis, MN, USA). SYTOX^TM^ Green nucleic acid stain was obtained from Thermo Fisher Scientific.

### RNA interference

EA.hy926 cells were seeded into 24-well plates at a density of 3 × 10^4^ cells/500 μl/well and incubated in 5% CO_2_ at 37 °C. The next day, human ELMO1 siRNA, which is a pool of three ELMO1 targeting siRNAs (Santa Cruz Biotechnology, Dallas, TX, USA), was transfected into the cells at 10 nM with Lipofectamine RNAiMAX (Thermo Fisher Scientific) according to the manufacturer’s protocol. Individual human ELMO1 siRNA #1 (sc-40525A) and #2 (sc-40525B) (SantaCruz Biotechnology) were also used to confirm the effects of ELMO1 knockdown. Untransfected cells, cells that were treated with Lipofectamine-only without siRNA (Mock), and siNT cells transfected with non-targeting siRNA [control siRNA-A, B and C (SantaCruz Biotechnology)] were used as controls for the ELMO1 knockdown cells. Experiments were repeated at least three times with each experimental group consisting of 3 replicates.

### Poly(I:C) treatment

EA.hy926 cells were transfected with siRNA as described above and cultured in DMEM with 10% FBS for one day. Cells were then starved in DMEM with 0.5% FBS overnight. Forty-eight hours after the transfection, cells were stimulated with 1 μg/ml of poly(I:C) (Tocris Bioscience, Bristol, UK) for the indicated time (3–4 wells/treatment).

### RNA sequencing and data analysis

Total RNA was extracted with RNeasy mini kit (Qiagen, Hilden, Germany) according to the manufacturer’s protocol. Library preparation and sequencing were performed by the High Throughput Sequencing Facility at UNC Chapel Hill. Each experimental group consisted of 3 biological replicates (total n = 18), and approximately 500 ng of total RNA was used per sample for library preparation with KAPA Stranded mRNA-Seq Kit (Roche) following the manufacturer’s protocol. Paired-end raw sequences of 100 bp were acquired by NextSeq 2000 (Illumina, San Diego, CA). Each sample yielded an average of ~28.3 million paired-end reads (range: 19.6–33.4 million), with a mean unique alignment rate of 92.7% across all samples, ensuring sufficient depth for gene- and transcript-level analysis. Subsequent demultiplexing was performed using bclconvert with a 1-mismatch threshold to segregate reads based on indexes. Quality control (QC) of the raw FASTQ files was performed using FastQC, with assessments of per base sequence quality, GC content, adapter contamination, and sequence duplication levels. FastQC reports indicated no significant adapter contamination or quality issues; thus, adapter trimming and read-length filtering were not required. These raw sequences were aligned to the human GRCh38 reference genome using the STAR aligner, followed by annotation using Gencode v44. Quantification of gene expression levels was conducted with the Stringtie transcriptome aligner. Utilizing a Python script (prepDE.py) provided by Stringtie, count matrices for genes and transcripts were generated from the output files. The count matrices were imported into R for preprocessing, normalizing, and quality filtering before differential gene expression analysis was performed using linear models with appropriate Bioconductor packages. Genes with total counts <100 across all samples were excluded, and those with maximum counts per million (CPM) < 1 were filtered out. Normalization was conducted using the Trimmed Mean of M-values (TMM) method in edgeR using default parameters. The normalized data was prepared for linear modeling using the voom transformation method, which estimates the mean–variance relationship in log2 CPM and assigns observation-level weights. Differential expression was assessed using limma’s linear modeling framework with empirical Bayes moderation, and the DREAM was applied for robust estimation of fixed and random effects across multifactorial experimental designs. Additionally, gene set enrichment analysis was performed using the Database for Annotation, Visualization, and Integrated Discovery (DAVID) [40, 41]. Gene expression data were deposited in the NCBI Gene Expression Omnibus (GEO) database (accession no: GSE299038).

### Quantitative reverse transcription PCR (qRT-PCR)

Total RNA was extracted from the cells with Trizol (Thermo Fisher Scientific) according to the manufacturer’s protocol. One-step quantitative RT-PCR was performed in a total 20 μl mixture containing 100 ng of RNA, 5 U of MultiScribe Reverse Transcriptase (Thermo Fisher Scientific) and 1× iTaq Universal Probes Supermix (Bio-Rad Laboratories, Hercules, CA, USA) with 7500 Real-Time PCR system (Thermo Fisher Scientific) or QuantStudio 3 Real-Time PCR system (Thermo Fisher Scientific). To detect each gene, TaqMan Gene Expression Assays Hs00174103_m1 for *CXCL8* and Hs00404992_m1 for *ELMO1* were purchased from Thermo Fisher Scientific. Gene-specific primers and probe for *GAPDH* (forward primer: 5’-GAAGGTGAAGGTCGGAGTC-3’, reverse primer: 5’-GAAGATGGTGATGGGATTTC-3’ and a TaqMan probe: FAM-CAAGCTTCCCGTTCTCAGCC-TAMRA) were purchased from Eton Bioscience (San Diego, CA, USA) and Thermo Fisher Scientific, respectively.

### Enzyme-linked immunosorbent assay (ELISA)

Cytokine concentrations in the cell culture media were quantified with DuoSet ELISA kits for human IL-8/CXCL8 and IFN-β (R&D Systems, Minneapolis, MN, USA) according to the manufacturer’s instructions.

### Western blot

Cells were lysed with RIPA buffer (50 mM Tris-HCl pH 8.0, 150 mM NaCl, 0.5% Sodium Deoxycholate, 0.1% SDS and 1% NP-40) with cOmplete^TM^ protease inhibitor (Roche, Basel, Switzerland). Proteins were separated by SDS-PAGE in 4–20% Mini-PROTEAN TGX precast protein gels (Bio-Rad Laboratories) and transferred to Immobilon-P PVDF membranes (MilliporeSigma). The membranes were then blocked in Tris-buffered saline (20 mM Tris, 150 mM NaCl, pH 7.4) with 0.05% Tween-20 (TBST) containing 3% BSA (MilliporeSigma), followed by incubation with primary antibodies overnight at 4 °C. After washing with TBST, the membranes were incubated with horseradish peroxidase (HRP)-conjugated anti-rabbit or anti-mouse antibody (1:2000, #7074 or #7076, Cell Signaling Technology, Danvers, MA, USA). Signals were detected with SuperSignal West Pico PLUS chemiluminescent substrate (Thermo Fisher Scientific) using Odyssey Fc (LI-COR, Lincoln, NE, USA). Primary antibodies against Elmo1 (1:1000, #14457), cleaved caspase-8 (1:1000, #9496), cleaved caspase-3 (1:1000, #9661), caspase-3 (1:1000, #9662), and caspase-7 (1:1000, #9492) were purchased from Cell Signaling Technology, an antibody against full-length caspase-8 (1:1000, #AG-20B-0057) was from AdipoGen Life sciences (San Diego, CA, USA), an anti-COL5A1 (1:1000, sc-166155) was from Santa Cruz Biotechnology, and an anti-PLOD1(LH1) (1:1000, NBP2-38770) was from Novus Biologicals (Centennial, CO, USA). Original western blots are shown in supplemental materials.

### Cell proliferation assay

EA.hy926 cells were plated on a 96-well plate at the density of 5 × 10^3^ cells/100 μl/well and incubated in 5% CO_2_ at 37°C. The next day, transfection of siRNA was performed as described above. Forty-four hours after the transfection, 5 μM 5-ethynyl-2’-deoxyuridine (EdU) was added to the medium and the cells were cultured for 4 h. Incorporation of EdU to newly synthesized DNA was detected with Click-iT^TM^ EdU Proliferation Assay for Microplates (Thermo Fisher Scientific) according to the manufacturer’s instructions. Fluorescent signal was detected with BioTek Synergy HT microplate reader (Agilent, Santa Clara, CA, USA). Each value was normalized to cell numbers quantified by staining with 1 μg/ml 4′,6-Diamidino-2-phenylindole dihydrochloride (DAPI) (MilliporeSigma).

### Cell cycle assay

1 × 10^6^ cells were harvested and fixed in 70% ethanol at −20 °C overnight then washed with phosphate-buffered saline (PBS) containing 1% FBS and stained with 5 μg/ml DAPI (MilliporeSigma). Samples were analyzed by LSRFortessa (BD Biosciences, Franklin Lakes, NJ, USA) or Attune NxT (Thermo Fisher Scientific). The cell cycle was analyzed by FlowJo software (BD Biosciences).

### Apoptosis assay

Apoptotic cells were visualized by terminal deoxynucleotidyl transferase dUTP nick end labeling (TUNEL) assay using ApopTag Red In Situ Apoptosis Detection Kit (MilliporeSigma) according to the manufacturer’s protocol. Early and late apoptosis/cell death were detected with RealTIme-Glo^TM^ Annexin V Apoptosis and Necrosis Assay (Promega, Madison, WI, USA) according to the manufacturer’s protocol. Early apoptosis was detected through the exposure of phosphatidylserine on the outer layer of the membrane, while late apoptosis/cell death was detected by the compromised membrane integrity. Fluorescent and chemiluminescent signal was detected with Varioskan LUX microplate reader (Thermo Fisher Scientific).

### Statistics

Statistical analyses were performed by one-way analysis of variance (ANOVA) followed by Tukey–Kramer’s Honest Significant Difference test. The sample size was determined based on our preliminary experiments. Data are shown as mean ± SD. *p* < 0.05 was considered as statistically significant. Data were analyzed using JMP Pro software version 17 (SAS Institute, Cary, NC, USA).

## Results

### Effects of ELMO1 knockdown on global gene expression before and after TLR3 stimulation

**Fig. 1:**
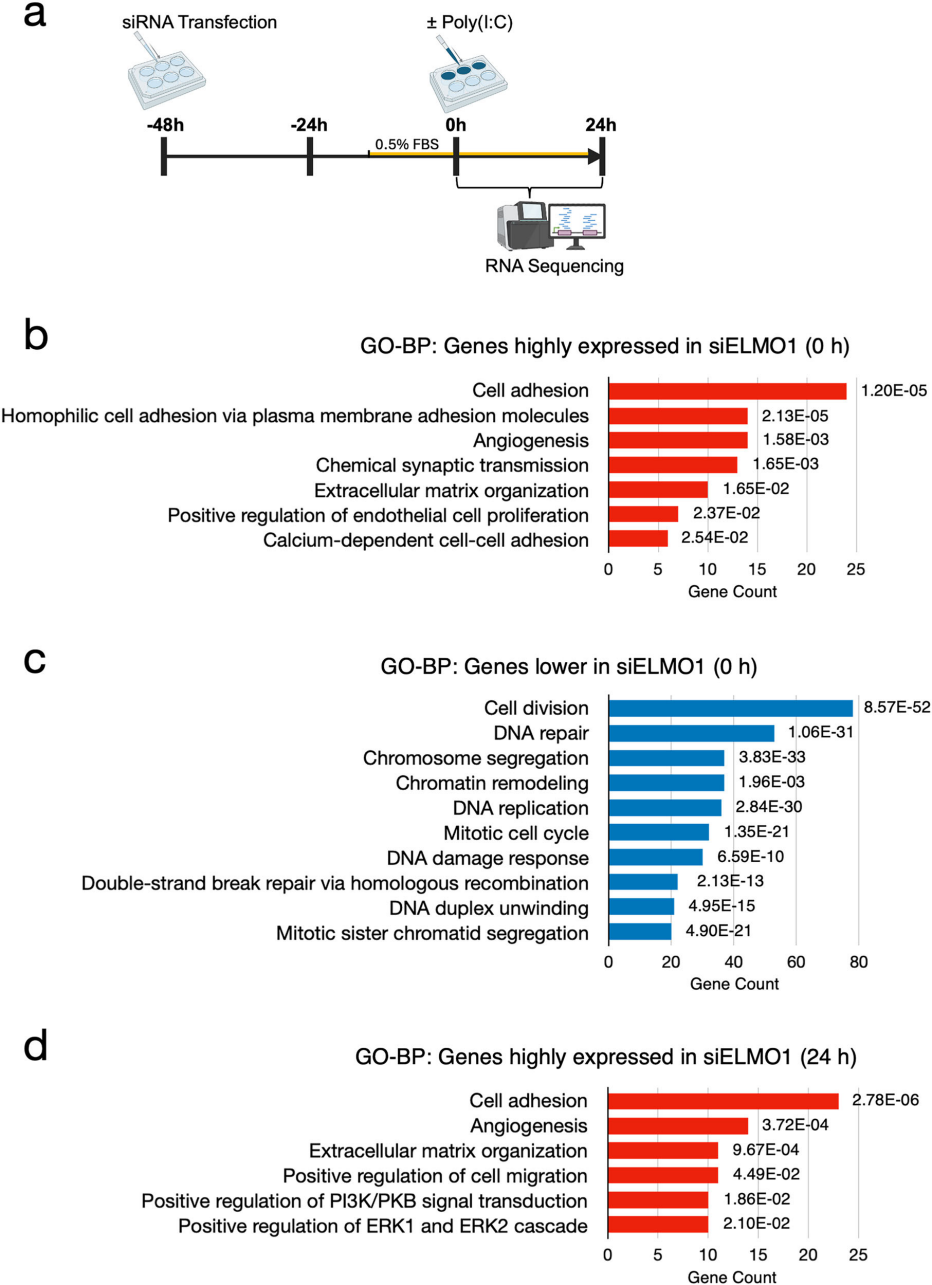
Transcriptome profiling of ELMO1 knockdown endothelial cells before and after stimulation with a TLR3 agonist poly(I:C). **a** Experimental design. EA.hy926 human endothelial cells were transfected with ELMO1 siRNA (siELMO1) and incubated for 48 h, followed by stimulation with 1 μg/ml of poly(I:C) for 24 h. Cells before (0 h) and after (24 h) the poly(I:C) stimulation were analyzed by RNAseq. Cells treated with the transfection reagent without siRNA (Mock) and those transfected with non-targeting siRNA (siNT) were used as controls. The image was created with BioRender.com. **b** Gene ontology (GO) analyses of genes highly expressed in siELMO1 compared to Mock and siNT at 0 hour. **c** GO analyses of genes less expressed in siELMO1 compared to Mock and siNT at 0 hour. **d** GO analyses of genes highly expressed in siELMO1 compared to Mock and siNT at 24 h. Genes were subjected to GO analyses using DAVID (false discovery rate (FDR) < 0.05). FDR for each gene set is indicated in the graphs. *N* = 3 per group. No significant GO terms were detected for the genes expressed at lower levels in siELMO1 cells at 24 h.

To examine whether ELMO1 is involved in endothelial inflammation, we transiently transfected ELMO1-targeting siRNA into an EA.hy926 human umbilical vein endothelial cell (HUVEC) line and stimulated the cells with poly(I:C), a TLR3 agonist which mimics viral RNA, at 48 h after the transfection (Fig. 1a). Cells treated with transfection reagent without siRNA (Mock) and cells transfected with non-targeting siRNA (siNT) were used as controls. In the ELMO1 siRNA-transfected cells (siELMO1), ELMO1 was reduced by approximately 85% at both mRNA and protein levels and remained low at 24 h after the poly(I:C) stimulation (Supplementary Figs. S1a and S1b).

We then performed RNAseq analysis of the Mock, siNT and siELMO1 cells before (0 h) and after (24 h) the poly(I:C) stimulation to analyze the global effects of ELMO1 knockdown on endothelial cells. Differentially expressed genes (DEGs) analyses (Fold change > 2, *p* < 0.05) revealed 260 genes were significantly higher and 490 genes were significantly lower in siELMO1 cells compared with both the Mock and siNT cells before the poly(I:C) stimulation (0 h). Collagen (*COL5A1*, *COL13A1*, *COL5A2, COL4A1*) and integrin (*ITGB8*) were among the highly expressed genes (Supplementary Fig S2a and Table S1). GO (gene ontology) analyses indicated cell adhesion and extracellular matrix organization were enriched in siELMO1 (Fig. 1b, Supplementary Fig. S3a). On the other hand, cell cycle and DNA replication-related genes, *E2F2*, *PKMYT1*, *CDK1*, *PBK*, *DEPDC1*, *DSCC1*, *ASF1B*, *DEPDC1B*, *FANCB*, *PLK1*, *SKP2*, *UBE2C* and *CDKN3* had lower expression in siELMO1 cells than in Mock and siNT cells (Supplementary Fig S2b and Table S2). GO analyses showed decreased expression of cell division and cell cycle genes in siELMO1 than in Mock and siNT cells (Fig. 1c, Supplementary Fig S3b).

At 24 h after poly(I:C) treatment, DEG analysis showed expression of 226 genes were higher and 82 genes were lower in siELMO1 cells compared with the Mock and siNT cells. Collagen (*COL5A1*, *COL4A1*), *THBS1* and *VWF* were higher in siELMO1 cells (Supplementary Fig S2c and Table S3). On the other hand, redox genes including *SLC7A11* and *TXN* were lower in siELMO1 cells than in siNT at 24 h (Supplementary Fig S2d and Table S4). SLC7A11 is a membrane transporter that imports extracellular cystine in exchange for intracellular glutamate. GO analyses showed that genes high in siELMO1 cells were enriched with cell adhesion and ECM organization, while no statistically significant terms were detected for the genes expressed at lower levels in siELMO1 cells (Fig. 1d, Supplementary Fig S3c). DEG analysis between 0 hour and 24 h revealed poly(I:C) increased 1736 genes in the Mock cells (Supplementary Table S5). Among them, transcript levels of inflammatory genes, including *CCL5*, *IL6*, *CXCL1*, *CXCL2*, and *CXCL8*, were robustly increased by poly(I:C) at 24 h. These genes were less expressed in siNT than in Mock cells, and significantly higher in siELMO1 cells than in siNT cells (Supplementary Fig S4a). The lower inflammatory gene expression in siNT is likely because of the increased cell death in the siNT cell population as shown below (Fig. 4). GO analyses indicated that innate immune response and inflammatory response genes were upregulated in Mock cells at 24 h (Supplementary Fig S4b).

### Knockdown of ELMO1 increases expression of ECM genes but inhibits cell proliferation genes

**Fig. 2:**
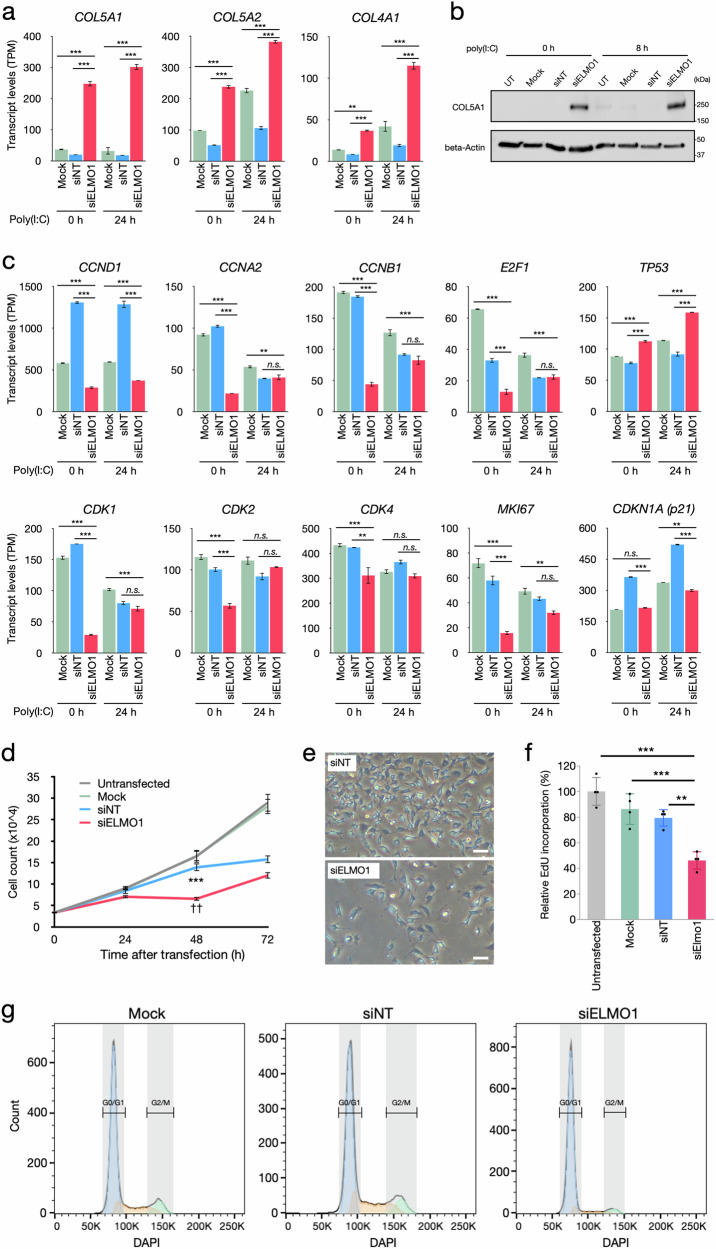
Knockdown of ELMO1 increases ECM and inhibits endothelial cell proliferation. **a** Transcript levels of collagen genes before (0 h) and after (24 h) the poly(I:C) treatment. Data are based on the RNAseq analysis shown in Fig. 1b. Transcript levels of each gene are shown as TPM (transcript per million). Data are mean ± SE (*N* = 3). **p < 0.01, ***p < 0.001. **b** Western blot of COL5A1 before (0 h) and 8 h after the stimulation with poly(I:C). Beta-Actin was detected as a loading control. **c** Transcript levels of selected genes associated with cell cycle before (0 h) and after (24 h) the poly(I:C) treatment. Data are based on the RNAseq analysis shown in Fig. 1c. Transcript levels of each gene are shown as TPM (transcript per million). Data are mean ± SE (*N* = 3). **p < 0.01, ***p < 0.001, *n.s*. not significant. **d** Cell numbers of untreated, Mock, siNT and siELMO1 cells after the transfection of siRNA. 3 × 10^4^ cells were plated on a 6-well plate and siRNA transfection was performed the next day. Cell numbers were determined by hemocytometer at the indicated time point after the transfection. Data are mean ± SE (*N* = 4). ***p < 0.001 vs. Untransfected and Mock, ^††^*p* < 0.01 vs. siNT. **e** Representative images of the siNT and siELMO1 cells at 48 h in (**d**) are shown. Bar = 100 μm. **f** Proliferation of siRNA-transfected cells detected by incorporation of EdU into newly synthesized DNA. Cells were labeled with 5 μM of EdU 44–48 h after the transfection. Data are mean ± SD (*N* = 4). **p < 0.01, ***p < 0.001. **g** Cell cycle analysis at 48 h after the transfection of siRNA. The histograms are representative of three independent experiments.

Quantification of each gene’s expression levels showed that collagen genes, such as *COL5A1*, *COL5A2* and *COL4A1* were significantly higher in siELMO1 than in Mock and siNT (Fig. 2a). *PLOD1*, which encodes Lysyl hydroxylase-1 (LH1), an enzyme essential for procollagen formation, was also higher in siELMO1 (Supplementary Fig S5a). Western blots confirmed the higher expression of COL5A1 and PLOD1(LH1) in siELMO1 than in Mock and siNT (Fig. 2b and Supplementary Fig. S5b).

RNAseq also showed that many cell cycle regulatory genes, including *CCND1*, *CCNA2*, *CCNB1*, *CDK1*, *CDK2*, *CDK4,* and *E2F1*, were significantly lower in siELMO1 than in Mock and siNT (Fig. 2c). A cell cycle maker gene *MKI67* coding for Ki-67 was also significantly lower in siELMO1 treated cells, while *TP53*, a cell cycle suppressor, was higher in siELMO1 treated cells (Fig. 2c). In accordance with the expressions of cell cycle regulatory genes, siELMO1 treated cells showed fewer cells compared to the untransfected, Mock and siNT cells (Fig. 2d, e). Incorporation of EdU was significantly lower in siELMO1 treated cells than the Mock and siNT cells (Fig. 2f). Cell cycle analysis also showed that the cell population in S phase was significantly smaller in siELMO1 than Mock and siNT cells (5.3 ± 2.7% vs. 10.8 ± 3.3% and 17.3 ± 2.7%, respectively, *p* < 0.05) (Fig. 2g). The G2/M population was also smaller in the siELMO1 cells by more than 80% compared to Mock and siNT cells (1.9 ± 1.4% vs. 11.0 ± 1.7% and 9.8 ± 1.4%, *p* < 0.05), whereas the proportion of G0/G1 population was significantly larger in siELMO1 than in Mock and siNT (88.9 ± 3.0% vs. 76.9% ± 3.6% and 68.2 ± 3.0%, *p* < 0.05) (Fig. 2g). These results indicate that ELMO1 is involved in the G1/S transition in EA.hy926 endothelial cells.

### Knockdown of ELMO1 shows restored inflammatory responses in TLR3-stimulated endothelial cells

**Fig. 3:**
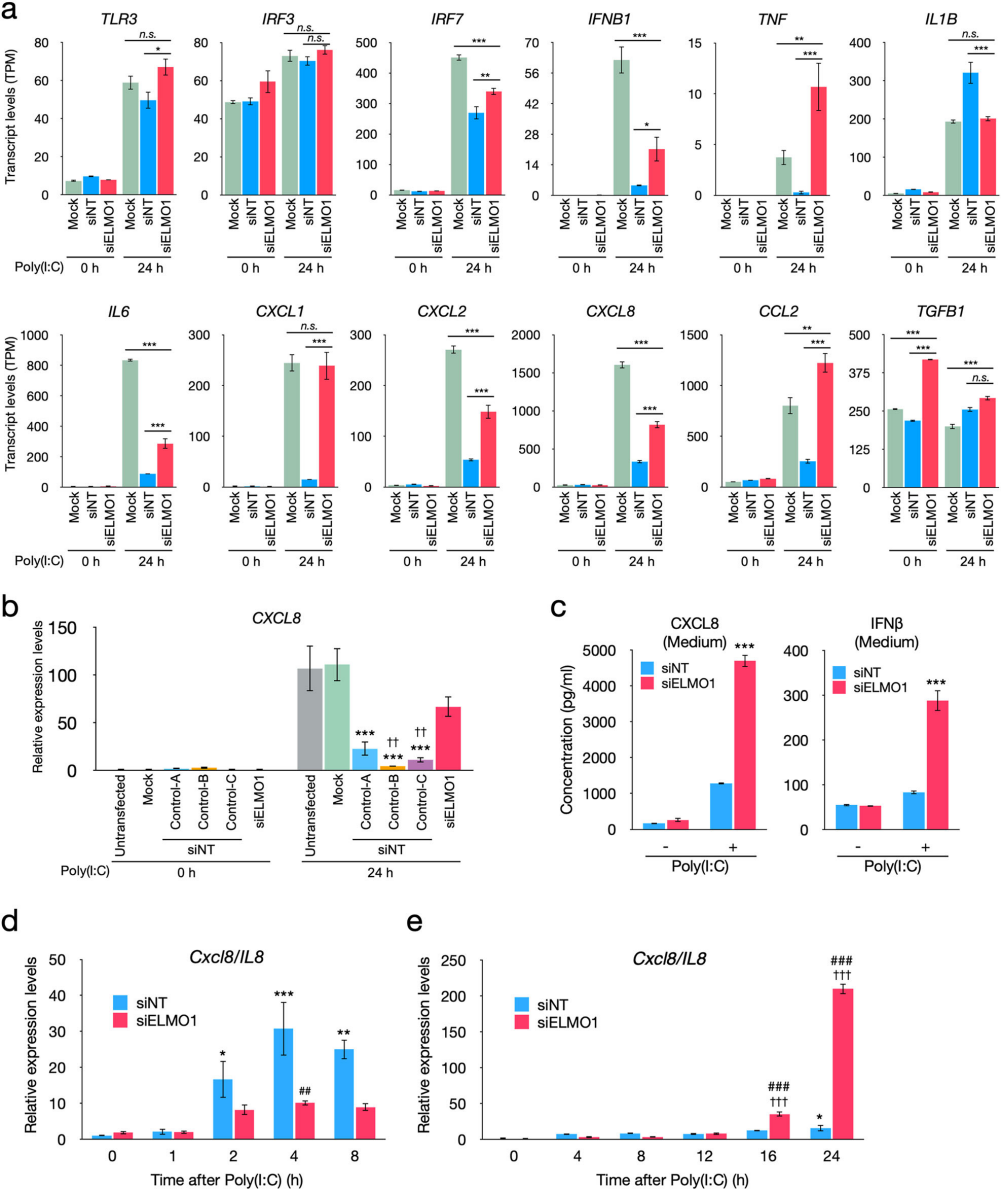
Effects of ELMO1 knockdown on inflammatory response. **a** Transcript levels of selected genes associated with inflammatory responses in Mock, siNT and siELMO1 transfected EA.hy926 cells before (0 h) and after (24 h) the poly(I:C) treatment. Data are based on RNAseq analysis shown in Fig S4. Transcript levels of each gene are shown as TPM. Data are mean ± SE (*N* = 3). *p < 0.05, **p < 0.01, ***p < 0.001, *n.s*. not significant. **b** Effects of three different non-targeting siRNAs (Control siRNA-A, B and C) on the expression of *CXCL8* in EA.hy926 cells before (0 h) and after (24 h) the poly(I:C) treatment. Data are mean ± SE (*N* = 4). ***p < 0.001 vs. Untransfected and Mock, ^††^*p* < 0.01 vs. siELMO1. **c** Effects of ELMO1 knockdown on the protein levels of CXCL8 and IFNβ in the medium detected by ELISA. Cells were transfected with siRNA (siNT or siELMO1) and 48 h later further stimulated with 1 μg/ml of poly(I:C) for 24 h. Data are mean ± SE (*N* = 4). ***p < 0.001 vs. siNT. Effects of ELMO1 knockdown on the mRNA levels of *CXCL8* after the poly(I:C) treatment in the early (**d**) and late (**e**) time points. Transcript levels of *CXCL8* were determined by qRT-PCR. Data are mean ± SE (*N* = 3). *p < 0.05, **p < 0.01, ***p < 0.001 vs. siNT at 0 hour; ^†††^*p* < 0.001 vs. siELMO1 at 0 hour ; ^##^*p* < 0.01, ^###^*p* < 0.001 vs. siNT at the same time point.

RNA sequencing showed poly(I:C) treatment robustly increased the levels of inflammatory gene transcripts, including *TLR3*, *IRF7*, *IFNB1*, *TNF*, *IL6*, *CXCL1*, *CXCL2*, *CXCL8* and *CCL2* at 24 h, but the increase was less in siNT cells, while these genes were significantly higher in siELMO1 cells than in siNT cells at 24 h (Fig. 3a). *IL1B* was also upregulated by poly(I:C), but was higher in siNT than in Mock and siELMO1 (Fig. 3a).

Transfection of siRNA is known to induce low levels of inflammatory responses through TLR3, independently of the siRNA sequence [42, 43]. In the siNT treated cells, transfection of the non-targeting siRNA slightly increased the expression of *CXCL8* compared to untransfected and Mock cells (Supplementary Fig S6). The level of *CXCL8* also showed a small increase in siELMO1 treated cells (Supplementary Fig S6), confirming that transfection of siRNA caused a mild inflammation.

When stimulated with poly(I:C) at 48 h after the transfection of siRNA, transcripts of *CXCL8* robustly increased in the untreated and Mock cells. The upregulation of *CXCL8* was significantly less in siNT cells compared to the untransfected or Mock cells, while restored in siELMO1 (Fig. 3b). All three control non-targeting siRNAs supplied by the manufacturer showed a reduction in the poly(I:C)-induced upregulation of *CXCL8*, indicating that the reduction is likely due to the introduction of foreign nucleic acids, rather than a sequence-dependent off-target effect of the siRNAs (Fig. 3b). In this experimental condition, mock cells were stimulated only once with poly(I:C), but cells treated with siNT and siELMO1 were stimulated twice, the first hit with siRNA and the second hit with poly(I:C) (both stimulate TLR3). Thus, siNT is considered as a negative control for the siELMO1 treated cells. Consistent with the transcript levels, concentrations of CXCL8 and IFNβ proteins in the culture medium were significantly higher in siELMO1 treated cells compared to the siNT cells stimulated with poly(I:C) (Fig. 3c). In the early time points (0–8 h) after the stimulation with poly(I:C), the siNT cells showed higher upregulation of *CXCL8* than siELMO1 treated cells (Fig. 3d). In the late time points (12–24 h), the *CXCL8* level robustly increased in siELMO1 treated cells, reaching to a 10 times higher level than siNT at 24 h (Fig. 3e).

### ELMO1 knockdown protects endothelial cells from poly(I:C)-induced apoptosis

**Fig. 4:**
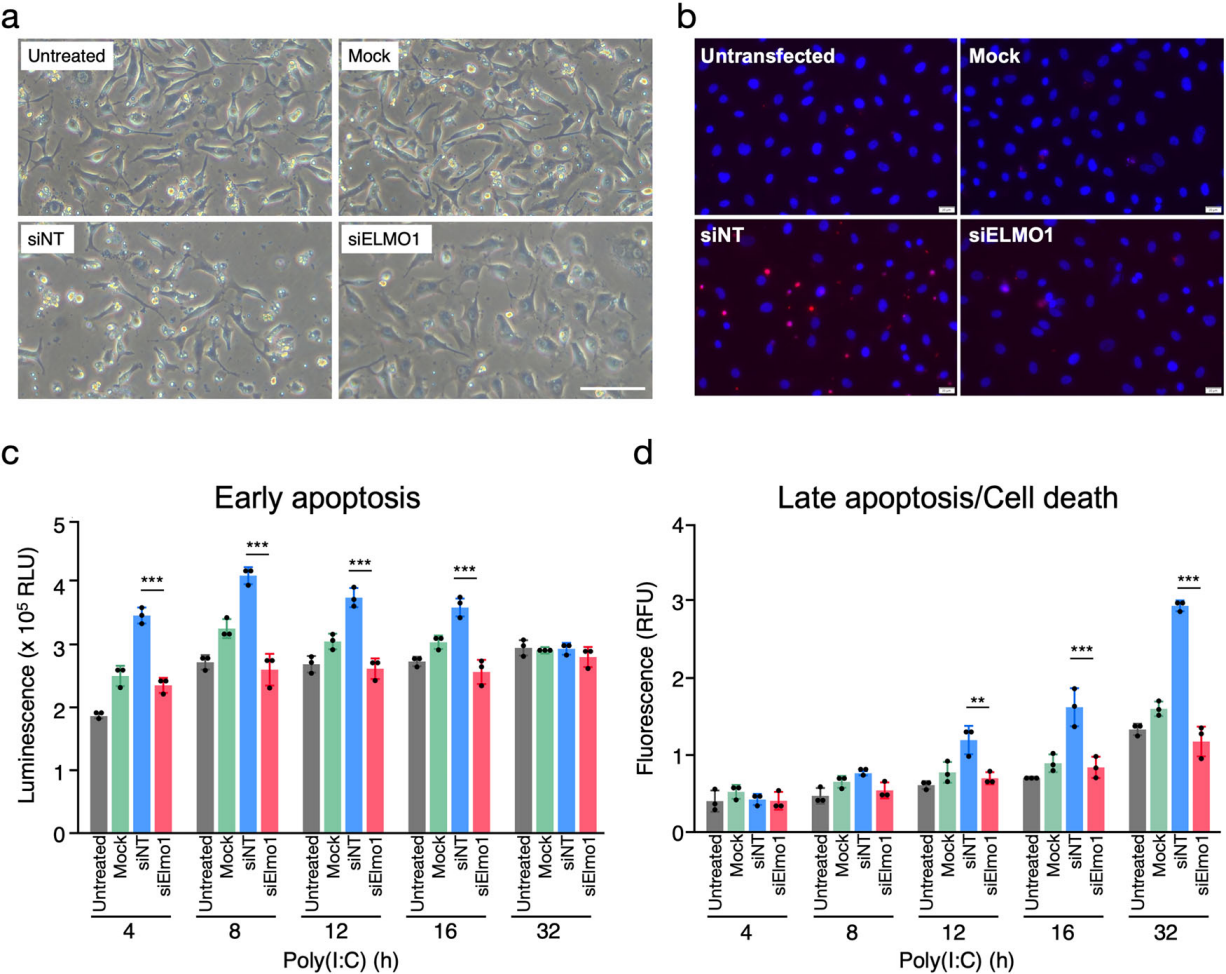
Knockdown of ELMO1 protects endothelial cells from apoptotic cell death induced by poly(I:C). EA.hy926 cells were transfected with siRNA and incubated for 48 h, followed by stimulation with 1 μg/ml poly(I:C) for 24 h. **a** Representative images of untransfected, Mock, siNT and siELMO1 cells at 24 h after the addition of poly(I:C). Bar = 100 μm. **b** Detection of apoptotic cells. At 24 h after the addition of poly(I:C), cells were fixed with 2% PFA, followed by TUNEL staining (Red). Nuclei were visualized with DAPI staining (Blue). Bar = 50 μm. **c**, **d** Detection of early apoptosis and late apoptosis/cell death induced by poly(I:C). Apoptosis/cell death was detected at the indicated time points after the stimulation with poly(I:C). Early apoptosis was detected through the exposure of phosphatidylserine on the outer layer of the membrane, while late apoptosis/cell death was detected by the compromised membrane integrity. Data are mean ± SD (*N* = 3). ***p < 0.001, **p < 0.01.

Twenty-four hours of poly(I:C) stimulation induced massive cell death in the siNT cells (Fig. 4a). The SYTOX Green staining confirmed the increase of membrane-compromised/dead cells in siNT cells compared to untreated and mock cells (Supplementary Fig. S7). The cell death observed in the siNT was not caused by the toxicity of liposome or serum starvation because Mock cells showed much less death than siNT cells (Fig. 4a and Supplementary Fig. S7). The cell death was not due to the sequence-dependent off-target effects of the siNT, since all the non-targeting siRNAs, including siRNA targeting the *ASB4* gene, which is not expressed in EA.hy926 cells (0 TPM in RNAseq data), induced similar levels of cell death after the poly(I:C) treatment among the non-targeting siRNA controls (not shown). A previous study reported that 1 μg/ml poly(I:C) induces only slight cell death in resting HUVECs, whereas pre-treatment with 1 μg/ml poly(I:C) for 24 h followed by re-stimulation with the same concentration of poly(I:C) induced significant apoptosis [44]. Similarly, transfection of siNT activated TLR3, as indicated by upregulation of *CXCL8* after the transfection of siRNAs (Supplementary Fig. S6). The stimulation with poly(I:C) subsequent to the siNT transfection induced apoptosis in the siNT cells, as confirmed by TUNEL staining (Fig. 4b). Strikingly, the induction of apoptosis was significantly inhibited in siELMO1 treated cells (Fig. 4a, b, Supplementary Fig. S7). The siELMO1 treated cells showed more rounded morphology compared to the untreated, mock and siNT cells (Fig. 4a, Supplementary Fig. S7), possibly reflecting the increased production of ECM. After the stimulation with poly(I:C), early apoptotic cells were significantly higher in siNT than in Mock at 4–16 h (Fig. 4c), while late apoptotic/necrotic cells became significantly higher in siNT at 12–32 h (Fig. 4d). Thus, siNT cells show early inflammation peaked at 4 h (Fig. 3d), and subsequent apoptosis peaked at 8 h (Fig. 4c) that led to cell death after 12 h (Fig. 4d). The lower inflammation in siNT cells at 24 h (Fig. 3a) is likely because of cell death. Both early and late apoptosis/cell death were significantly suppressed in siELMO1 cells, and these apoptosis/cell death were at comparable levels to the untransfected cells (Fig. 4c, d). Together, these results demonstrate that ELMO1 mediates TLR3-induced apoptotic cell death in EA.hy926 cells.

### Regulation of cell death by ELMO1 in endothelial cells

**Fig. 5:**
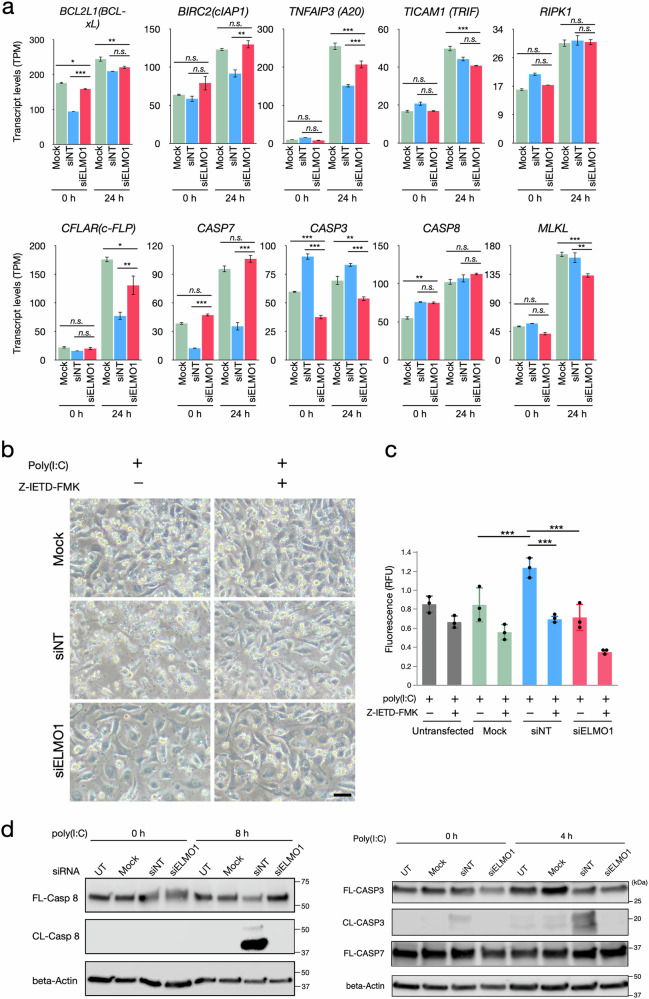
Knockdown of ELMO1 inhibits apoptosis by suppressing caspase-8 activation downstream of TLR3. **a** Transcript levels of selected genes associated with apoptosis before (0 h) and after (24 h) the poly(I:C) treatment. Transcript levels of each gene are shown as TPM (transcript per million). Data are mean ± SE (*N* = 3). *p < 0.05, **p < 0.01, ***p < 0.001, *n.s*. not significant. **b** Representative images of Mock, siNT and siELMO1 cells 24 h after the poly(I:C) stimulation. Cells were treated with or without 20 ng/ml of caspase-8 inhibitor Z-IETD-FMK throughout the experiment. Bar = 20 μm. **c** Cell death in (b) was quantified as in Fig. 4d. Data are mean ± SD (*N* = 3). ***p < 0.001. **d** Western blot of full-length and cleaved caspase-8, -3 and -7 before (0 h) and after (4 h, 8 h) the stimulation with poly(I:C). Beta-Actin was detected as a loading control. Images are representative of three independent experiments.

RNAseq results showed expression levels of apoptosis-inducing genes, such as *BCL2L1*, *BARC2*, *CFLAR* and *CASP7* were lower in siNT cells but restored in siELMO1 cells before and after the stimulation with poly(I:C), suggesting that apoptosis-related molecules are affected in siNT while knockdown of ELMO1 rescued them (Fig. 5a). Activation of TLR3 induces extrinsic apoptosis through the recruitment and cleavage of caspase-8 [30, 45]. In fact, a caspase-8 inhibitor Z‑IETD‑FMK reduced the poly(I:C)-induced cell death in siNT, indicating the involvement of caspase-8 in the cell death process (Fig. 5b, c). Consistent with the low incidence of apoptosis and cell death in the untransfected cells and Mock cells (Fig. 4a–d), poly(I:C) did not cause cleavage of caspase-8 in the untransfected and Mock cells (Fig. 5d). On the other hand, poly(I:C) led to cleavage of caspase-8 while reducing the amount of full-length caspase-8 in siNT (Fig. 5d). This is because siNT cells were preconditioned with TLR3 stimulation by siRNA transfection, but untransfected and Mock cells were not [44]. Knockdown of ELMO1 dramatically blocked the poly(I:C)-induced cleavage of caspase-8 shown in siNT (Fig. 5d). Similarly, the cleavage of caspase-3, an effector caspase downstream of caspase-8, was induced by poly(I:C) in siNT, but was inhibited in the ELMO1 knockdown cells (Fig. 5d). Cleavage of caspase-7 was not detected in this experimental condition (Fig. 5d). To confirm the effects of ELMO1 knockdown by the pooled siRNAs, the effects of individual ELMO1 siRNA #1 and #2 were also tested in the EA.hy926 cells. Both siRNAs decreased the expression of ELMO1 and upregulated COL5A1 (Supplementary Fig. S8a). When stimulated with poly(I:C), both ELMO1 knockdown cells showed protection from cell death, consistent with the pooled siRNAs (Supplementary Fig. S8b). The cleavage of caspase-3 and 8 were also inhibited in both the ELMO1 knockdown cells (Supplementary Fig. S8c). These data indicates that ELMO1 plays a role in the caspase-8 activation pathway in the endothelial cells downstream of TLR3.

## Discussion

In this study, we demonstrated that knockdown of ELMO1 in endothelial cells causes quiescence, protecting the cells from TLR3-induced cell death, and restoring inflammatory responses, suggesting that ELMO1 plays a pivotal role in the decision of proliferation or quiescence, as well as life or death under the TLR3-induced inflammation. Our results show a previously unknown role of ELMO1 in regulating cell fate through modulation of caspase-8 activity downstream of TLR3 signaling. Extensive studies have been performed on the multiple functions of caspase-8 in cell survival, inflammation, and various forms of cell death, highlighting the importance of caspase-8 in cell fate decisions [23–25]. Whether ELMO1 directly interacts with the components of death-inducing complexes, including RIPK1 and caspase-8, needs further elucidation.

In order to downregulate expression of ELMO1, we transfected siRNA, which is a widely accepted and efficient method to reduce target transcripts. However, transfection of oligonucleotide including siRNA can induce unintended, non-specific effects, such as off-target silencing and induction of innate immune response [46]. We believe the possibility of off-target silencing by oligonucleotides was minimized in the current study, because homology search of the three ELMO1 siRNA sequences using the NIH Basic Local Alignment Search Tool (BLAST) did not find overlapping sequences except for ELMO1, and the concentration of siRNA used in the current experiments was 10 nM, which is much lower than the 100 nM that could affect non-specific transcripts [47]. Nevertheless, RNAseq showed that expression levels of many genes were significantly changed in cells transfected with non-targeting siRNA compared to those in mock-transfected cells, indicating that transfection with siRNA oligonucleotides exerts non-specific effects in the experimental system through the TLR activation, retinoic acid-inducible gene-I (RIG-I)/melanoma differentiation-associated protein 5 (MDA5) and/or other unknown mechanisms [48]. While comparing the effects of targeted siRNA (siELMO1) to the control non-targeted siRNA (siNT) is a well-accepted procedure, the analysis requires careful consideration, hence we included Mock transfection without oligonucleotides and untransfected controls in our experiments.

Consistent with a previous study that showed sequential simulation of HUVECs with poly(I:C) induced apoptosis [44], cells transiently transfected with oligonucleotides such as siRNA were sensitive to poly(I:C)-induced cell death. Since clustering of TLR3 in the membrane rafts is important for downstream signaling [49], the first stimulus might sensitize the cells by clustering TLR3, which leads to overactivation of TLR3 and other death receptors, switching from a pro-inflammatory to a cell death pathway on the second stimulus. Another possibility is that the first stimulus promotes assembly of intracellular death-inducing complexes including RIPK1 and caspase-8, sensitizing the cells to the second stimulus.

Poly(I:C) and its derivatives are extensively examined in multiple clinical studies as adjuvants in cancer immunotherapy and vaccines, based on the effects of poly(I:C) that activates innate immune responses and induce apoptosis in cancer cells [50, 51]. The efficacy depends on the type of tumor cells and stages. Our results indicate that reduction of ELMO1 levels in cancer cells may arrest them in a quiescent state and dampen the sensitivity of the cells to poly(I:C)-containing anticancer therapy.

We showed that ELMO1 knockdown causes G1/S arrest in the human endothelial cells. The result is consistent with multiple studies showing that ELMO1 is upregulated in a wide range of tumor cells both in vivo and in vitro, and that knockdown of ELMO1 suppressed cell proliferation in various human cancer epithelial cell lines, including breast cancer, colorectal cancer and liver cancer [52–54]. How ELMO1 mechanistically influences the cell cycle is not fully understood, although the involvement of ELMO1 in cell cycle regulation is in accordance with previous reports that show Rac1 is required for G1/S progression through induction of cyclin D1 [55], and that inhibition of Rac1 causes cell cycle arrest [56, 57]. The crosstalk between cell cycle and cell-extracellular matrix interaction could also play a role. We observed knockdown of ELMO1 significantly upregulated *COL5A1* and *COL5A2*. Collagen V is known to inhibit the cell cycle in various cell types including endothelial cells [58, 59]. Another study also demonstrated that muscle satellite (stem) cells produce collagen V to inhibit cell cycle and maintain the cells in a quiescent state in a cell-autonomous manner [60]. The significant increase of *COL5A1* and *COL5A2* and other ECM components could inhibit cell cycle and induce quiescence in the ELMO1 knockdown cells.

Whether the downregulation of ELMO1 causes a quiescent state or a senescent state needs to be defined [61]. Quiescence is a “reversible” cell cycle arrest, while senescence is an “irreversible” loss of proliferative potential [61]. Whether the cell cycle arrest we observed in the ELMO1 knockdown cells is reversible or irreversible is not clear in the experimental condition. In our study, knockdown of ELMO1 resulted in reduced proliferation, higher production of inflammatory cytokines, and resistance to apoptosis, which overlap with hallmarks of senescence [62]. On the other hand, gene expression patterns of ELMO1 knockdown cells did not necessarily indicate senescence. For example, a senescence marker gene *CDKN2A* coding for p21 was lower in the ELMO1 knockdown cells than in the siNT cells (Fig. 2c). This inconsistency could be because our result reflects short-term effects of ELMO1 knockdown and cells are still under heterogeneous conditions in a transition to a full senescent status. A recent study showed that knockout of Elmo1 increased senescent cells in the colon of mouse colitis models [63]. This study also demonstrated that knockdown of ELMO1 in several intestinal epithelial cell lines promoted cell senescence and upregulated production of cytokines such as IFNβ, IL1β, IL8 and IL6, a phenotype known as Senescence-Associated Secretory Phenotype (SASP) [63]. Another study indicated that Rac1 is involved in senescence directly or indirectly via ROS generation and activation of p53 [64]. Together, high expression of ELMO1 is likely associated with cancer phenotypes, such as enhanced proliferation, migration and epithelial-to-mesenchymal transition (EMT), whereas low level of ELMO1 is associated with quiescent/senescent phenotypes represented by cell cycle arrest and altered ECM composition.

Considering the diverse functions of RAC1, which span from cell movement, cell cycle regulation, gene transcription, production of ROS and stabilization of cell-cell contacts, it is not surprising that suppression of ELMO1 affected many genes and pathways. Moreover, ELMO1-DOCK-RAC1 complex could directly affect gene transcription. A study has shown that Elmo1 interacts with Med31, a regulator of RNA polymerase II, affecting general gene transcription in mouse bone marrow-derived macrophages [65]. Another study demonstrated that DOCK8 interacts with STAT3 and promotes its activation and nuclear translocation, inducing STAT3-dependent gene expression in T cells [66]. RAC1 has also been shown to translocate to the nucleus and affect RNA processing [67]. ELMO1 could regulate transcript levels through these mechanisms as well.

Collectively, our results indicate that ELMO1 is involved in cell cycle, ECM composition, pro-inflammatory responses and cell death, raising a possibility that ELMO1-DOCK-RAC1 system could be a therapeutic target in endothelial dysfunction in cancer and inflammation. Since numerous GEFs and GAPs are known to regulate RAC1, how each molecule differentially regulates and fine-tunes the activation of RAC1 under various pathophysiological conditions needs further investigation.

## Supplementary information

Supplementary Figure S1

Supplementary Figure S2

Supplementary Figure S3

Supplementary Figure S4

Supplementary Figure S5

Supplementary Figure S6

Supplementary Figure S7

Supplementary Figure S8

Supplementary Table S1

Supplementary Table S2

Supplementary Table S3

Supplementary Table S4

Supplementary Table S5

Western Blot original images

## Data Availability

RNA sequencing data are deposited to the NCBI Gene Expression Omnibus (GEO) database (accession number: GSE299038).
