# Supplementary figures and images for: Downregulation of Engulfment and cell motility 1 (Elmo1) induces quiescence and resistance to poly(I:C)-induced apoptosis in endothelial cells
Source: Cell Death Dis. 2025 Dec 20;17(1):100. doi: 10.1038/s41419-025-08341-1 (PMC12847878; doi:10.1038/s41419-025-08341-1)

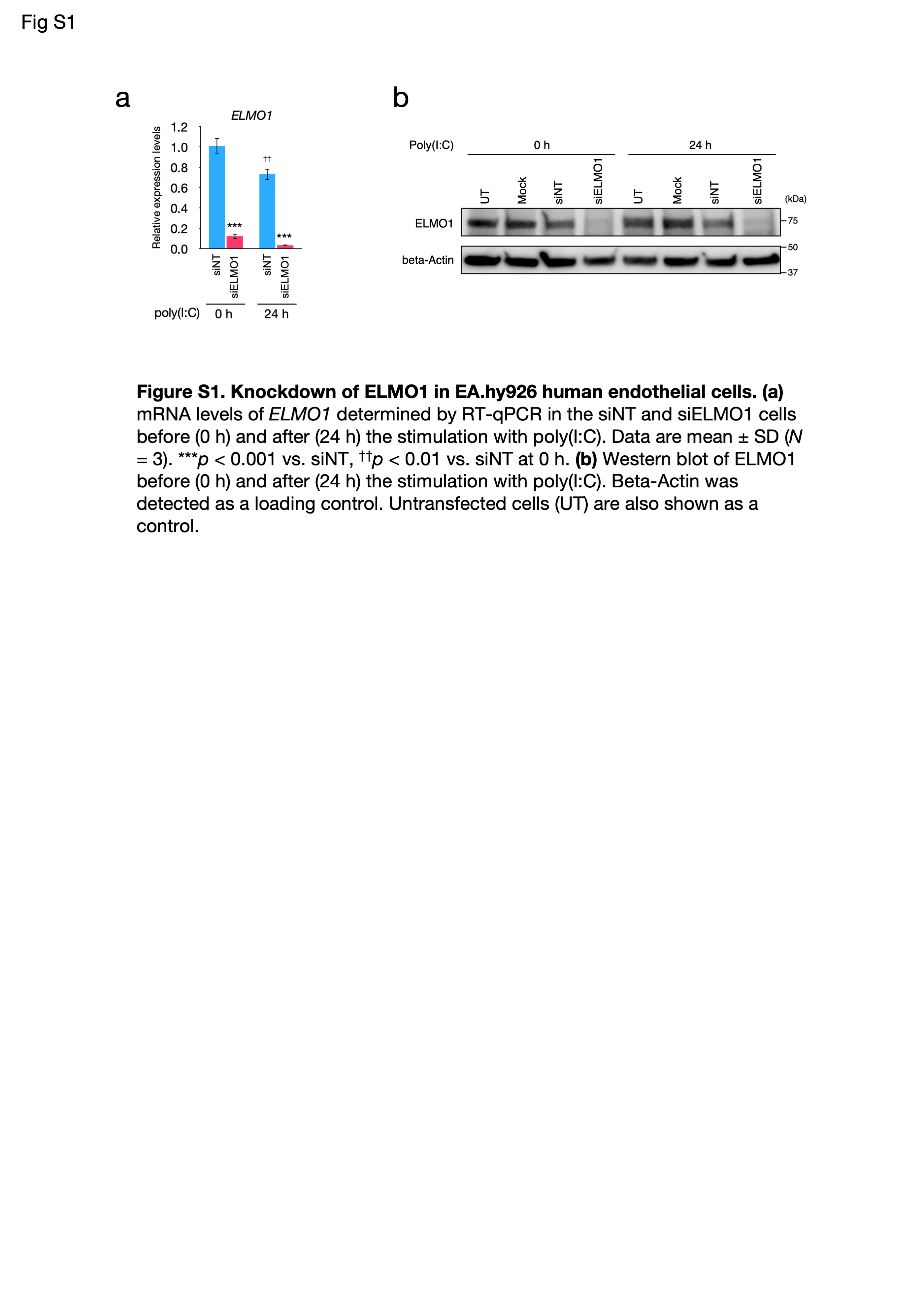

Supplement: Supplementary file 1 — Supplementary Figure S1 [file 41419_2025_8341_MOESM1_ESM.png]

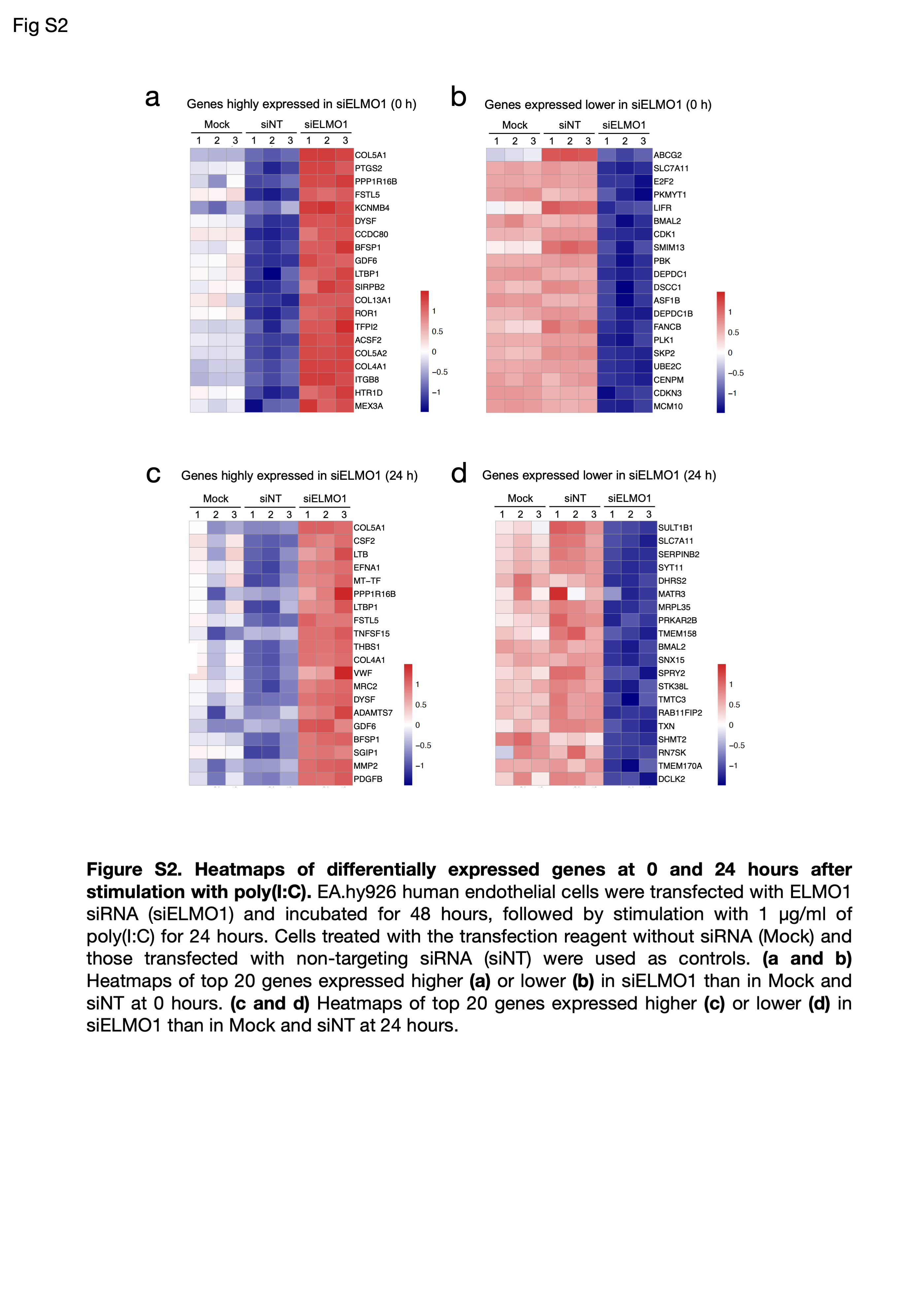

Supplement: Supplementary file 2 — Supplementary Figure S2 [file 41419_2025_8341_MOESM2_ESM.png]

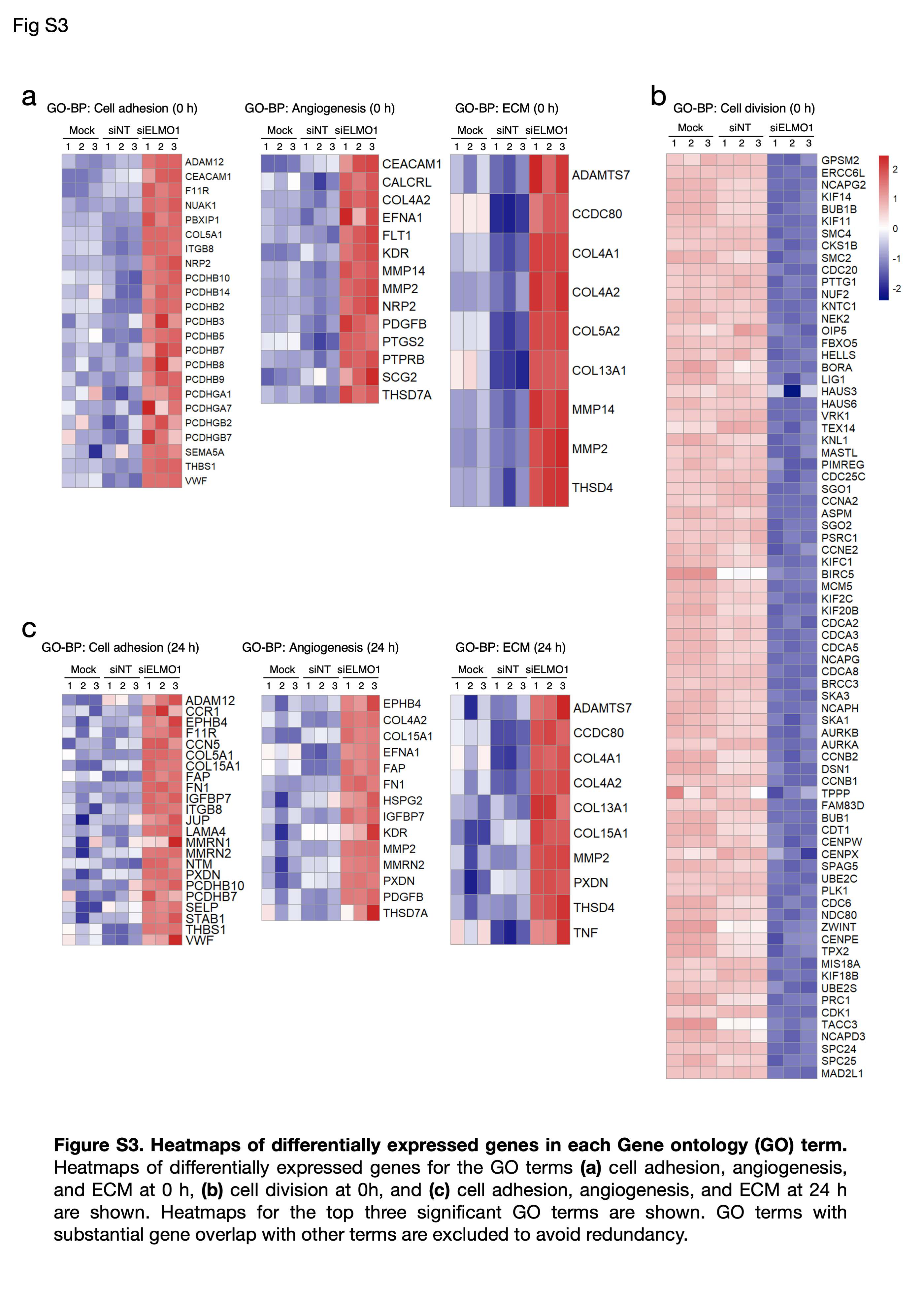

Supplement: Supplementary file 3 — Supplementary Figure S3 [file 41419_2025_8341_MOESM3_ESM.png]

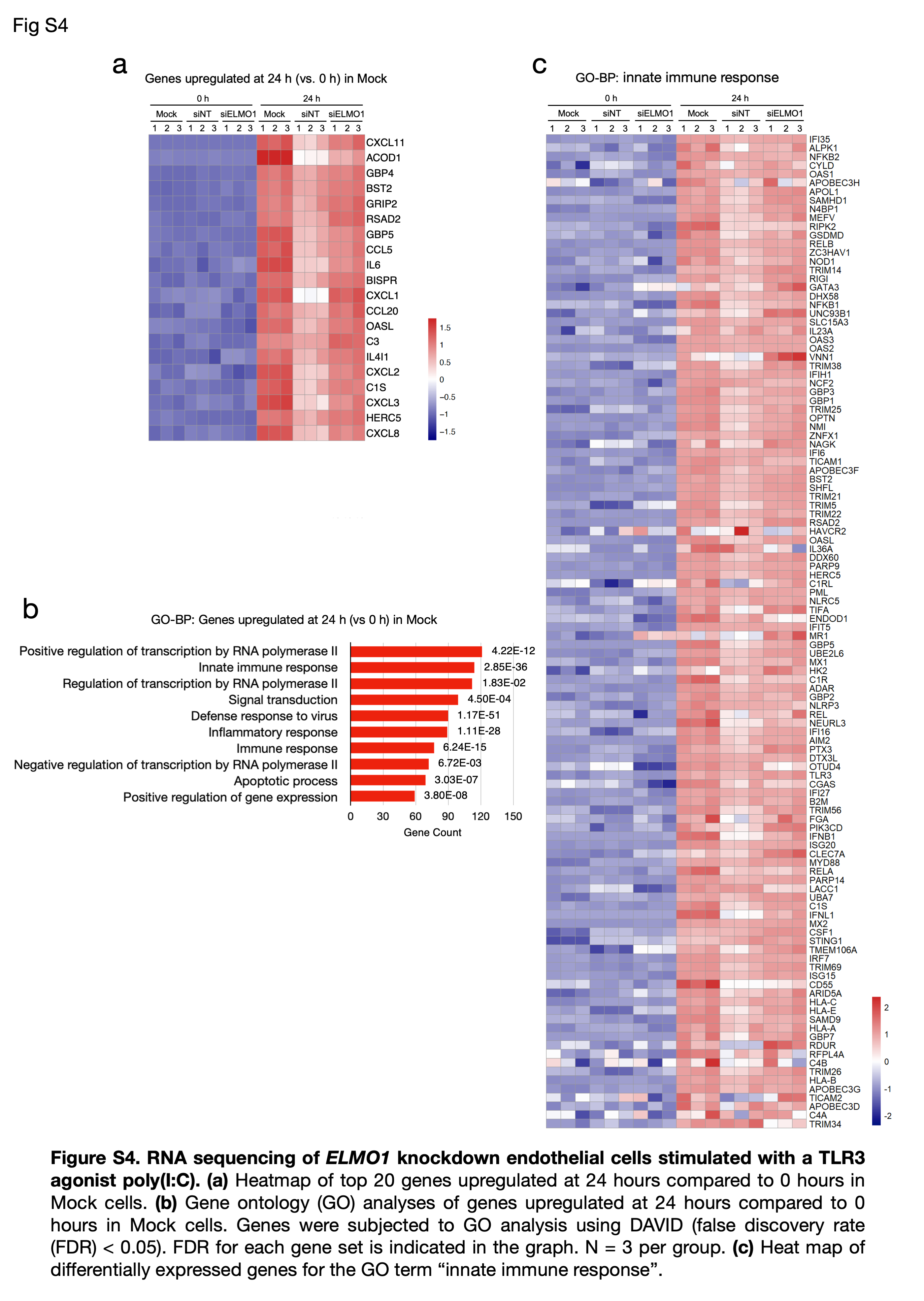

Supplement: Supplementary file 4 — Supplementary Figure S4 [file 41419_2025_8341_MOESM4_ESM.png]

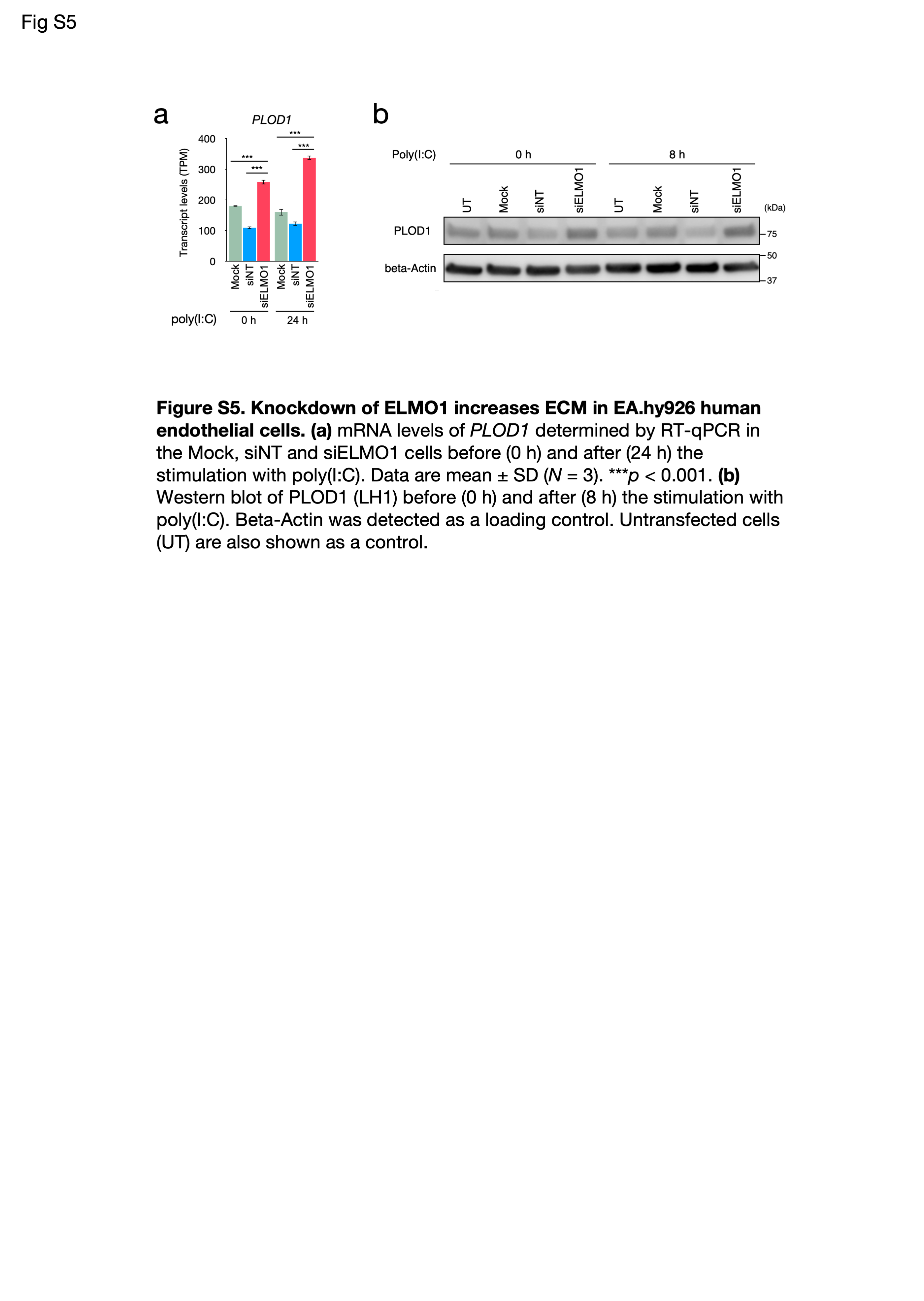

Supplement: Supplementary file 5 — Supplementary Figure S5 [file 41419_2025_8341_MOESM5_ESM.png]

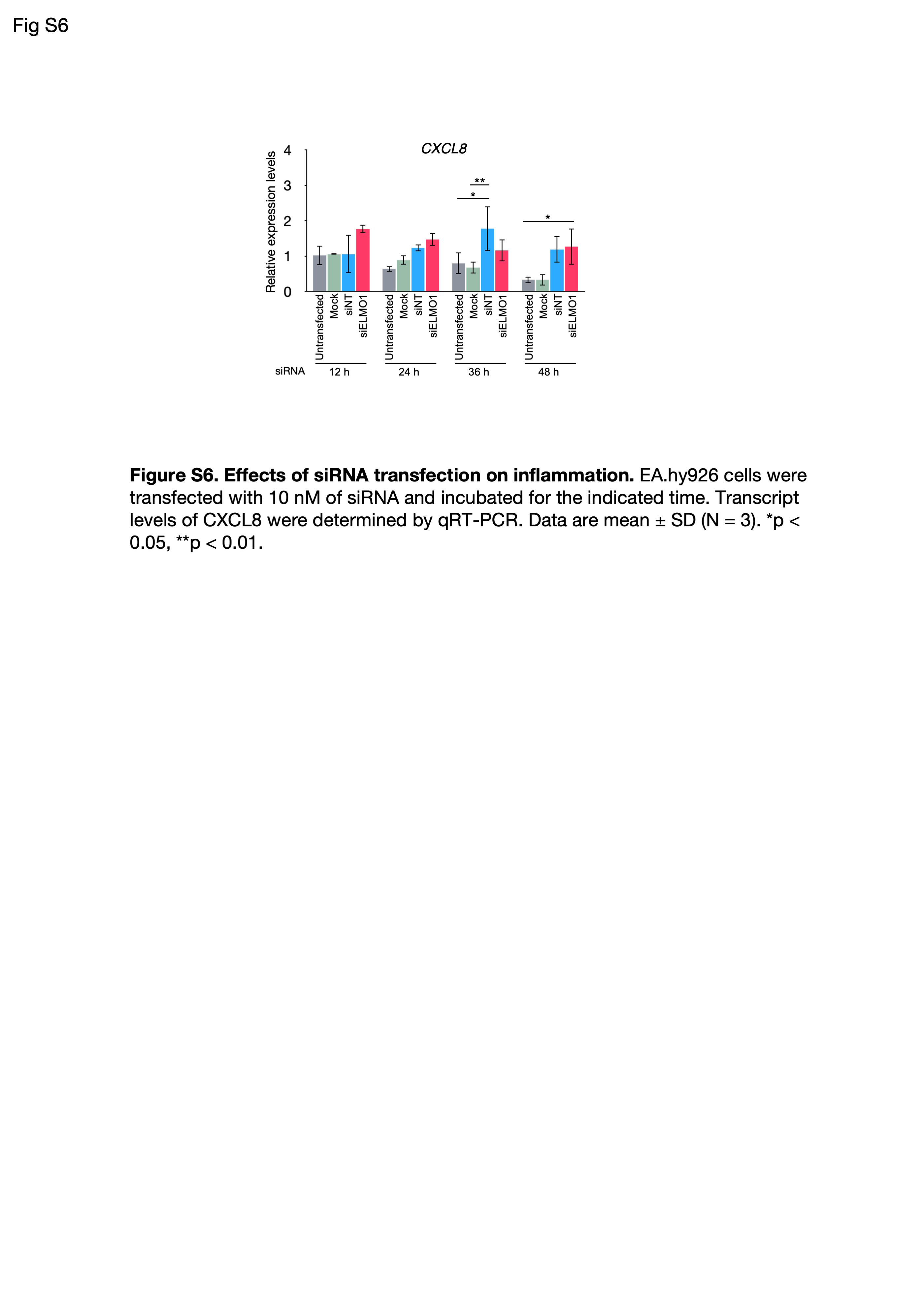

Supplement: Supplementary file 6 — Supplementary Figure S6 [file 41419_2025_8341_MOESM6_ESM.png]

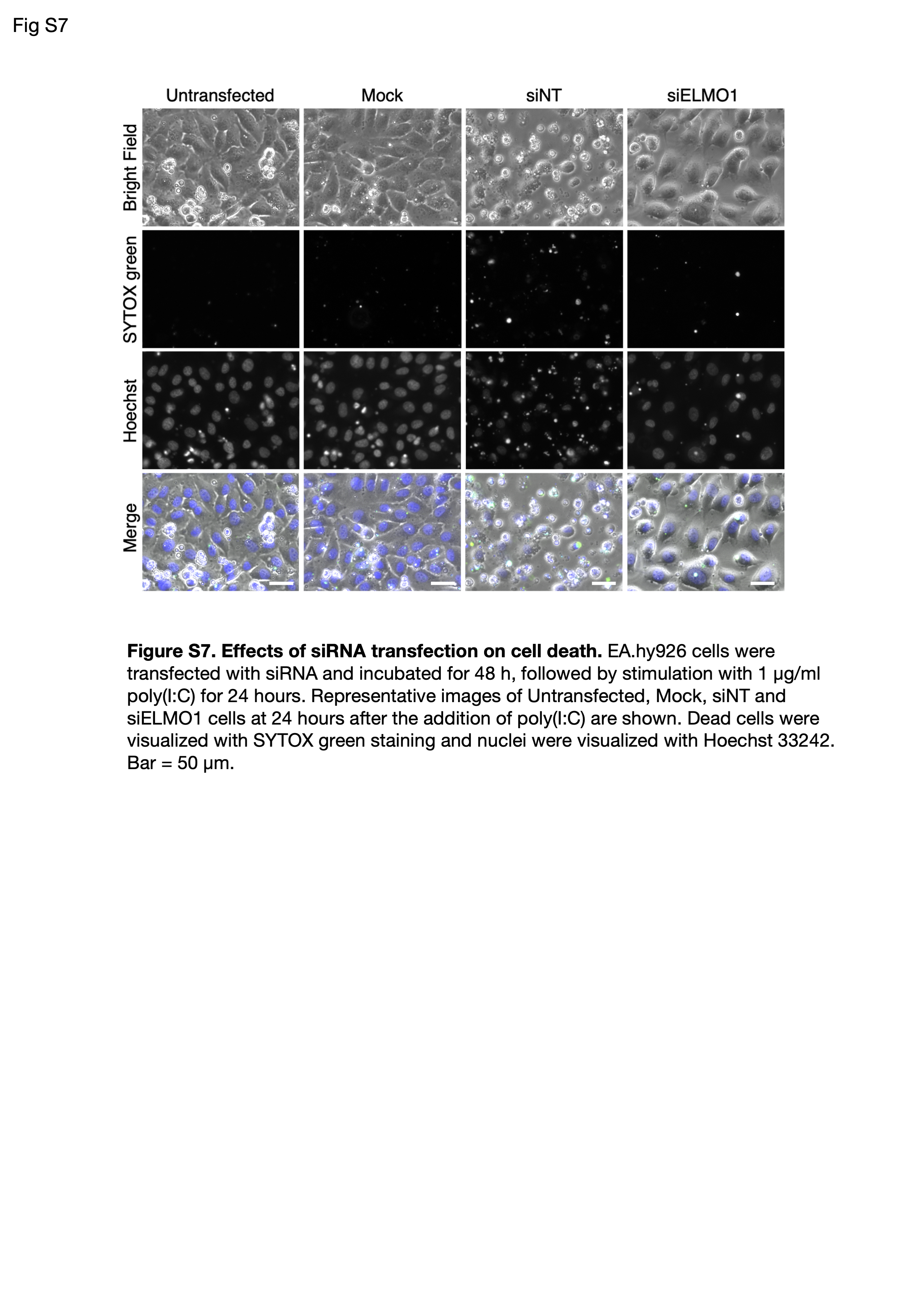

Supplement: Supplementary file 7 — Supplementary Figure S7 [file 41419_2025_8341_MOESM7_ESM.png]

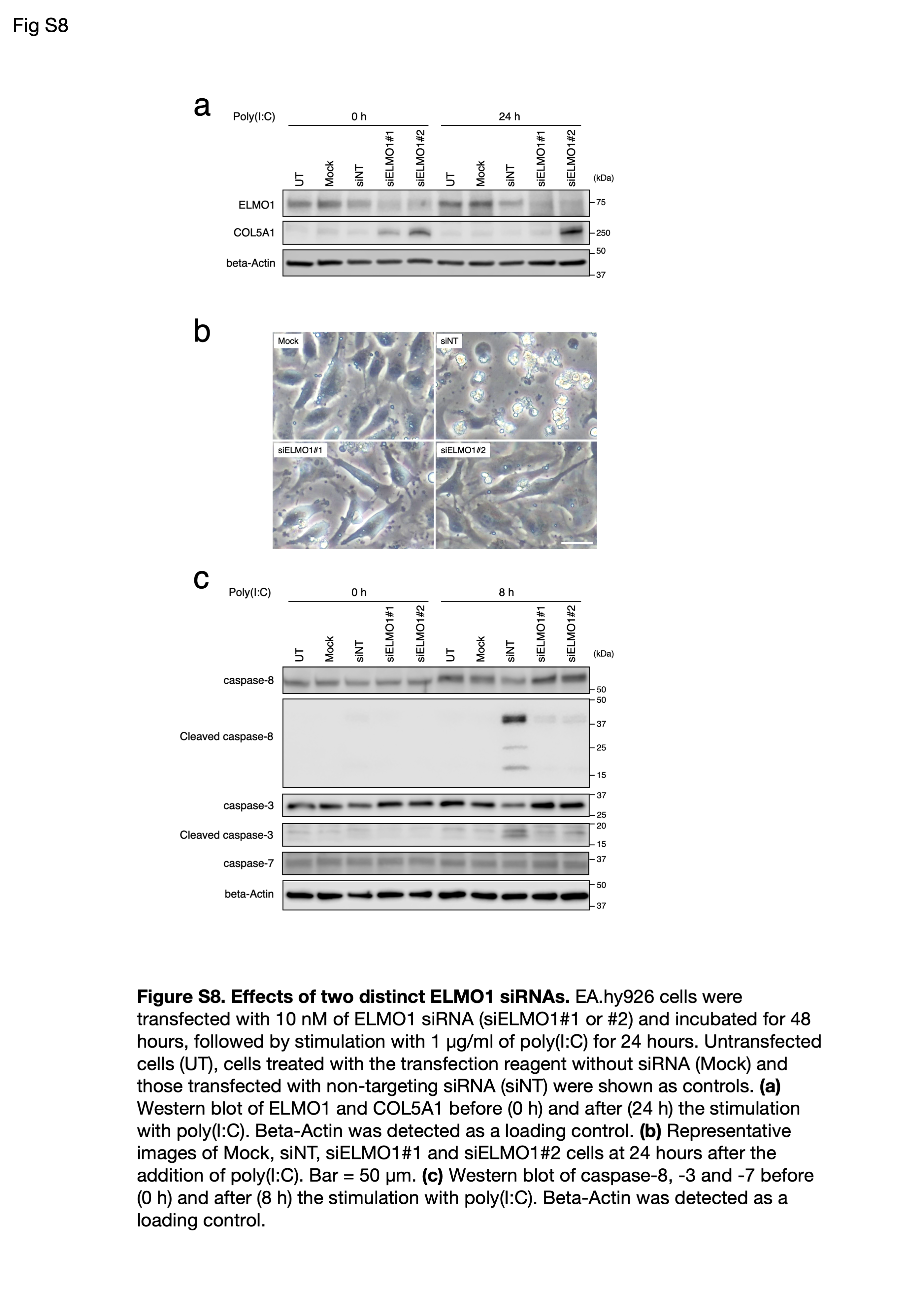

Supplement: Supplementary file 8 — Supplementary Figure S8 [file 41419_2025_8341_MOESM8_ESM.png]
