# Supplementary material for: Downregulation of Engulfment and cell motility 1 (Elmo1) induces quiescence and resistance to poly(I:C)-induced apoptosis in endothelial cells
Source: Cell Death Dis. 2025 Dec 20;17(1):100. doi: 10.1038/s41419-025-08341-1 (PMC12847878; doi:10.1038/s41419-025-08341-1)
Supplement: Supplementary file 9 — Supplementary Table S1 [file 41419_2025_8341_MOESM9_ESM.docx]

| **Table S1. Genes expressed higher in siELMO1 at 0 h** | | | |  |  |  |
| --- | --- | --- | --- | --- | --- | --- |
| Gene | 0H_Mock | 0H_siNT | 0H_siELMO1 | 24H_Mock | 24H_siNT | 24H_siELMO1 |
| COL5A1 | 120.27 | 70.35 | 885.61 | 115.96 | 62.36 | 1091.62 |
| PTGS2 | 17.96 | 5.98 | 73.35 | 233.80 | 35.64 | 121.32 |
| PPP1R16B | 4.44 | 2.05 | 18.71 | 1.87 | 1.44 | 13.60 |
| FSTL5 | 24.67 | 7.34 | 53.82 | 19.14 | 7.75 | 57.03 |
| KCNMB4 | 1.64 | 1.47 | 10.33 | 0.75 | 0.50 | 4.64 |
| DYSF | 50.32 | 20.78 | 141.65 | 49.23 | 27.74 | 148.73 |
| CCDC80 | 274.86 | 87.45 | 581.84 | 201.96 | 128.15 | 433.18 |
| BFSP1 | 9.93 | 4.41 | 26.34 | 8.32 | 4.65 | 23.04 |
| GDF6 | 17.56 | 6.73 | 39.39 | 5.61 | 5.91 | 29.41 |
| LTBP1 | 40.31 | 16.21 | 90.93 | 32.77 | 12.60 | 104.61 |
| SIRPB2 | 14.34 | 6.05 | 32.78 | 19.31 | 14.45 | 48.22 |
| COL13A1 | 12.11 | 4.96 | 25.21 | 5.82 | 5.83 | 19.92 |
| ROR1 | 18.92 | 7.76 | 38.95 | 7.49 | 6.73 | 21.80 |
| TFPI2 | 4.81 | 2.75 | 13.13 | 38.69 | 19.05 | 67.98 |
| ACSF2 | 7.72 | 4.02 | 18.70 | 7.97 | 3.66 | 15.87 |
| COL5A2 | 269.72 | 153.12 | 691.61 | 674.72 | 315.33 | 1115.69 |
| COL4A1 | 35.47 | 23.04 | 102.14 | 118.98 | 53.55 | 321.48 |
| ITGB8 | 22.50 | 15.18 | 66.62 | 11.85 | 13.90 | 49.89 |
| HTR1D | 16.90 | 7.93 | 34.55 | 4.45 | 3.91 | 9.57 |
| MEX3A | 11.03 | 6.10 | 26.23 | 6.59 | 5.25 | 15.34 |
| PDCD6IP-DT | 8.07 | 3.93 | 16.53 | 8.94 | 5.90 | 16.26 |
| THBS1 | 1436.17 | 1192.24 | 4842.05 | 1322.20 | 648.25 | 3964.99 |
| PALMD | 32.03 | 22.92 | 91.51 | 5.91 | 16.00 | 25.92 |
| CALCRL | 11.88 | 7.40 | 28.23 | 7.53 | 5.07 | 15.27 |
| KRBA2 | 10.62 | 6.08 | 22.33 | 7.64 | 6.83 | 13.28 |
| PDGFB | 17.87 | 14.62 | 52.97 | 15.47 | 11.61 | 52.16 |
| HIC1 | 15.94 | 9.43 | 34.11 | 8.24 | 7.10 | 14.46 |
| MR1 | 3.27 | 3.09 | 10.95 | 8.94 | 7.01 | 22.20 |
| RHOBTB1 | 11.87 | 9.21 | 32.44 | 4.83 | 4.92 | 11.94 |
| VWF | 5.58 | 3.77 | 13.03 | 9.80 | 4.63 | 26.88 |
| ST6GAL1 | 30.21 | 18.39 | 63.48 | 39.83 | 22.04 | 63.76 |
| ADAMTS7 | 22.09 | 17.55 | 59.28 | 17.76 | 15.75 | 82.00 |
| MMP2 | 439.28 | 393.38 | 1324.83 | 305.39 | 290.86 | 1334.13 |
| C11orf71 | 5.45 | 3.44 | 11.22 | 6.29 | 4.13 | 6.93 |
| GPR153 | 8.32 | 6.06 | 19.77 | 7.95 | 7.56 | 21.85 |
| UCP2 | 14.44 | 10.06 | 31.93 | 9.46 | 8.03 | 19.55 |
| PHETA1 | 12.54 | 9.49 | 30.08 | 9.56 | 10.11 | 19.02 |
| ABCA1 | 22.40 | 16.05 | 50.55 | 81.99 | 64.20 | 140.01 |
| ABCA8 | 5.99 | 5.27 | 16.33 | 2.22 | 2.46 | 7.02 |
| KDR | 5.18 | 7.03 | 21.70 | 5.86 | 12.09 | 27.32 |
| PCDHB2 | 17.31 | 11.89 | 36.49 | 16.15 | 13.26 | 22.95 |
| ZNF467 | 3.63 | 3.94 | 11.96 | 6.17 | 6.00 | 9.26 |
| YPEL2 | 22.43 | 21.24 | 63.46 | 25.65 | 19.97 | 66.41 |
| TNFSF15 | 26.83 | 21.21 | 63.21 | 22.52 | 26.45 | 190.72 |
| FAM3C2P | 6.18 | 6.22 | 18.45 | 7.78 | 6.93 | 11.50 |
| CUBN | 7.38 | 5.10 | 15.10 | 5.43 | 5.98 | 20.44 |
| BMF | 3.08 | 4.55 | 13.02 | 5.25 | 4.11 | 13.98 |
| DKK3 | 35.62 | 28.45 | 81.21 | 50.59 | 34.95 | 138.01 |
| TMSB4XP4 | 9.27 | 10.19 | 29.07 | 7.15 | 5.33 | 10.18 |
| THSD4 | 54.65 | 46.15 | 130.10 | 49.75 | 50.84 | 127.49 |
| EPB41L5 | 8.36 | 6.04 | 16.83 | 6.41 | 5.53 | 9.87 |
| FLT1 | 16.65 | 14.11 | 39.05 | 19.66 | 17.68 | 27.51 |
| SATB1 | 16.02 | 16.90 | 46.47 | 24.15 | 19.56 | 31.89 |
| FILIP1L | 12.78 | 9.97 | 27.23 | 65.90 | 33.18 | 57.23 |
| ZSWIM4 | 11.60 | 11.40 | 31.08 | 47.96 | 32.79 | 60.23 |
| CPA3 | 3.93 | 5.77 | 15.72 | 2.40 | 2.73 | 9.16 |
| F2R (PAR1) | 218.03 | 163.86 | 437.13 | 82.44 | 100.91 | 276.73 |
| MT-TP | 3.46 | 5.38 | 14.13 | 5.23 | 10.02 | 9.14 |
| CEACAM1 | 5.97 | 9.56 | 24.93 | 115.54 | 89.16 | 191.72 |
| FRAS1 | 4.03 | 7.19 | 18.62 | 4.90 | 9.50 | 29.74 |
| ARHGEF3 | 23.06 | 18.04 | 46.59 | 17.43 | 23.06 | 32.53 |
| NFIA | 22.08 | 20.92 | 54.01 | 9.07 | 13.76 | 21.60 |
| MMP14 | 178.25 | 153.77 | 392.77 | 200.43 | 287.15 | 360.55 |
| HMCN1 | 5.65 | 6.34 | 16.17 | 5.88 | 8.21 | 11.19 |
| DIXDC1 | 43.33 | 41.60 | 105.90 | 31.12 | 34.63 | 50.22 |
| C18orf32 | 15.40 | 14.88 | 37.59 | 18.20 | 17.38 | 27.32 |
| UNC13A | 30.31 | 25.53 | 64.47 | 23.39 | 33.29 | 46.88 |
| TDG | 26.91 | 25.76 | 64.74 | 31.68 | 23.35 | 44.92 |
| KCNJ2 | 28.19 | 24.43 | 61.38 | 16.09 | 20.77 | 27.35 |
| NRP2 | 109.98 | 101.29 | 252.37 | 206.09 | 199.67 | 221.73 |
| MTND1P23 | 3.13 | 4.17 | 10.38 | 3.97 | 4.37 | 5.02 |
| RNF152 | 25.16 | 20.70 | 51.08 | 12.54 | 26.80 | 31.18 |
| FZD2 | 35.38 | 38.41 | 94.56 | 18.55 | 22.38 | 37.03 |
| HYOU1 | 177.34 | 155.64 | 382.67 | 383.91 | 202.93 | 411.03 |
| MT-ND1 | 377.88 | 520.19 | 1255.78 | 727.20 | 852.70 | 890.67 |
| HBP1 | 38.28 | 33.06 | 79.79 | 66.31 | 39.62 | 77.98 |
| KCTD21 | 11.20 | 11.00 | 26.47 | 14.50 | 11.84 | 20.21 |
| SLC39A6 | 75.85 | 80.88 | 190.85 | 94.45 | 89.14 | 175.57 |
| WIPI1 | 34.85 | 32.07 | 75.51 | 49.17 | 35.53 | 59.68 |
| SORBS2 | 6.84 | 6.01 | 13.91 | 1.65 | 2.48 | 7.19 |
| CCDC103 | 7.73 | 6.91 | 15.91 | 6.07 | 6.22 | 10.61 |
| MAML2 | 18.70 | 17.91 | 41.16 | 32.77 | 31.61 | 47.20 |
| WIPF1 | 16.54 | 15.62 | 35.87 | 31.95 | 30.82 | 68.65 |
| MTND2P28 | 128.38 | 156.80 | 357.95 | 152.12 | 208.29 | 153.66 |
| FHL3 | 29.25 | 30.32 | 68.96 | 40.70 | 30.95 | 71.13 |
| BTBD19 | 23.85 | 22.32 | 49.96 | 30.22 | 20.38 | 43.40 |
| PBXIP1 | 55.42 | 50.30 | 111.99 | 48.58 | 53.95 | 97.16 |
| NUAK1 | 30.30 | 31.64 | 70.43 | 24.08 | 38.07 | 48.74 |
| ZNF385A | 13.45 | 16.67 | 37.06 | 18.61 | 19.05 | 35.40 |
| MT-RNR2 | 2751.32 | 3719.43 | 8237.32 | 5560.34 | 5084.44 | 8462.73 |
| F11R | 13.38 | 16.52 | 36.59 | 26.52 | 20.92 | 71.42 |
| MT-ND2 | 845.19 | 1058.52 | 2337.85 | 1119.01 | 1482.99 | 1052.27 |
| COL4A2 | 23.07 | 21.21 | 46.61 | 41.26 | 28.33 | 111.40 |
| FAM3C | 82.36 | 83.69 | 183.61 | 87.83 | 75.19 | 125.59 |
| LIMD2 | 6.48 | 7.85 | 16.84 | 17.66 | 16.52 | 33.83 |
| RNF44 | 47.30 | 55.71 | 119.06 | 65.22 | 70.14 | 101.88 |
| PTPRB | 79.88 | 75.87 | 161.97 | 165.91 | 137.17 | 184.26 |
| PPP1R3C | 8.44 | 10.16 | 21.41 | 2.21 | 4.05 | 5.54 |
| SLC27A1 | 8.52 | 8.19 | 17.22 | 11.45 | 10.83 | 15.15 |
| SLC38A7 | 29.58 | 30.40 | 63.83 | 47.26 | 43.86 | 68.62 |
| ANKRD42 | 16.68 | 16.23 | 33.80 | 17.70 | 24.19 | 27.00 |
| TTC17 | 97.33 | 116.43 | 237.11 | 164.18 | 140.75 | 321.73 |
| ANTXR1 | 191.73 | 213.52 | 433.97 | 108.89 | 155.82 | 319.02 |
| ARID3A | 16.77 | 21.38 | 43.35 | 14.55 | 19.62 | 27.30 |
