# Supplementary material for: Downregulation of Engulfment and cell motility 1 (Elmo1) induces quiescence and resistance to poly(I:C)-induced apoptosis in endothelial cells
Source: Cell Death Dis. 2025 Dec 20;17(1):100. doi: 10.1038/s41419-025-08341-1 (PMC12847878; doi:10.1038/s41419-025-08341-1)
Supplement: Supplementary file 10 — Supplementary Table S2 [file 41419_2025_8341_MOESM10_ESM.docx]

| **Table S2. Genes expressed lower in siELMO1 at 0 h** | | | |  |  |  |
| --- | --- | --- | --- | --- | --- | --- |
| Gene | 0H_Mock | 0H_siNT | 0H_siELMO1 | 24H_Mock | 24H_siNT | 24H_siELMO1 |
| ABCG2 | 22.54 | 84.34 | 10.36 | 15.35 | 21.90 | 9.77 |
| SLC7A11 | 456.39 | 482.42 | 59.84 | 177.69 | 277.61 | 47.05 |
| E2F2 | 12.45 | 12.93 | 1.78 | 8.39 | 6.81 | 4.16 |
| PKMYT1 | 40.11 | 32.02 | 4.75 | 28.43 | 21.42 | 18.26 |
| LIFR | 10.17 | 22.43 | 3.47 | 11.20 | 11.05 | 7.23 |
| BMAL2 | 490.02 | 422.94 | 66.97 | 623.38 | 637.09 | 218.69 |
| CDK1 | 116.13 | 142.24 | 23.23 | 84.65 | 65.29 | 57.08 |
| SMIM13 | 24.59 | 49.96 | 8.20 | 19.96 | 33.39 | 12.26 |
| PBK | 23.66 | 26.07 | 4.32 | 11.92 | 10.91 | 8.48 |
| DEPDC1 | 88.38 | 75.00 | 12.60 | 44.58 | 28.29 | 23.42 |
| DSCC1 | 10.72 | 13.16 | 2.24 | 6.11 | 7.45 | 3.66 |
| ASF1B | 60.98 | 51.49 | 8.88 | 53.99 | 31.80 | 34.49 |
| DEPDC1B | 12.97 | 14.76 | 2.56 | 7.13 | 6.73 | 5.20 |
| FANCB | 17.81 | 27.58 | 4.98 | 12.69 | 13.76 | 10.74 |
| PLK1 | 138.38 | 153.30 | 28.40 | 82.73 | 75.55 | 47.31 |
| SKP2 | 42.04 | 53.00 | 9.82 | 26.08 | 27.85 | 13.55 |
| UBE2C | 114.21 | 119.10 | 23.04 | 85.11 | 58.64 | 55.80 |
| CENPM | 12.81 | 12.70 | 2.47 | 8.70 | 7.43 | 5.51 |
| CDKN3 | 22.34 | 19.80 | 3.86 | 14.99 | 8.09 | 6.28 |
| MCM10 | 56.37 | 49.25 | 9.63 | 43.90 | 33.52 | 26.94 |
| KIF18B | 58.67 | 72.18 | 14.43 | 46.07 | 37.11 | 36.16 |
| SGO1 | 38.64 | 43.52 | 8.76 | 38.56 | 29.63 | 22.35 |
| TMTC3 | 102.49 | 117.06 | 23.60 | 74.94 | 89.84 | 33.04 |
| CDC20 | 162.12 | 164.52 | 33.20 | 108.30 | 81.21 | 65.91 |
| RAD51AP1 | 35.92 | 36.57 | 7.56 | 38.44 | 26.03 | 29.03 |
| ANLN | 272.14 | 304.04 | 62.83 | 164.30 | 145.10 | 108.05 |
| CCNA2 | 103.19 | 123.10 | 25.53 | 65.56 | 48.02 | 48.61 |
| RACGAP1 | 127.99 | 155.09 | 32.24 | 79.30 | 78.20 | 49.55 |
| SPC24 | 27.33 | 28.31 | 5.90 | 13.09 | 13.15 | 9.64 |
| GGCT | 29.72 | 54.73 | 11.41 | 28.29 | 41.23 | 17.10 |
| MRPL35 | 57.16 | 64.11 | 13.37 | 48.20 | 59.28 | 19.95 |
| DDIAS | 36.14 | 49.49 | 10.42 | 30.66 | 28.48 | 23.02 |
| EXO1 | 28.09 | 27.88 | 5.94 | 20.46 | 17.75 | 15.84 |
| ESPL1 | 108.93 | 101.35 | 21.80 | 111.85 | 93.48 | 74.15 |
| POLQ | 51.48 | 58.23 | 12.55 | 54.07 | 43.75 | 41.08 |
| NUF2 | 59.05 | 64.27 | 13.95 | 37.00 | 29.53 | 22.93 |
| CDKN2C | 14.90 | 12.37 | 2.70 | 8.64 | 5.98 | 4.24 |
| XRCC3 | 19.11 | 23.60 | 5.18 | 18.91 | 14.87 | 14.16 |
| CDCA8 | 70.15 | 67.53 | 14.86 | 48.07 | 35.23 | 36.53 |
| FANCD2 | 58.85 | 53.39 | 11.76 | 49.74 | 34.57 | 37.78 |
| SHCBP1 | 97.02 | 96.17 | 21.43 | 69.58 | 45.28 | 47.10 |
| SHMT2 | 202.22 | 200.21 | 44.92 | 329.64 | 218.63 | 80.79 |
| DHRS2 | 14.51 | 15.18 | 3.42 | 14.69 | 12.85 | 3.73 |
| NEK2 | 44.86 | 37.72 | 8.52 | 25.14 | 18.81 | 14.69 |
| FAM111B | 44.63 | 32.50 | 7.35 | 27.09 | 19.29 | 17.20 |
| APOBEC3B | 45.96 | 51.13 | 11.61 | 55.03 | 43.27 | 39.50 |
| MAD2L1 | 75.10 | 76.51 | 17.62 | 47.82 | 38.81 | 34.94 |
| CCNB1 | 155.59 | 160.09 | 36.89 | 112.28 | 79.22 | 70.65 |
| SYT11 | 18.53 | 17.83 | 4.14 | 14.07 | 18.17 | 3.79 |
| NEIL3 | 23.59 | 20.73 | 4.83 | 16.44 | 8.93 | 10.89 |
| NCAPH | 54.70 | 44.01 | 10.40 | 42.02 | 28.85 | 28.41 |
| SKA1 | 49.05 | 41.04 | 9.80 | 44.89 | 28.38 | 30.38 |
| ESCO2 | 13.00 | 13.20 | 3.16 | 13.73 | 8.44 | 11.30 |
| NDC80 | 60.32 | 50.77 | 12.31 | 44.59 | 27.28 | 31.90 |
| CDC25C | 25.78 | 27.72 | 6.73 | 19.60 | 12.52 | 14.39 |
| TRIP13 | 64.32 | 63.21 | 15.40 | 41.53 | 33.50 | 30.54 |
| KIFC1 | 44.69 | 39.04 | 9.54 | 36.59 | 24.15 | 23.73 |
| FAM83D | 118.57 | 140.73 | 34.47 | 95.45 | 62.21 | 60.48 |
| KIF2C | 98.04 | 83.24 | 20.49 | 85.81 | 44.92 | 54.58 |
| RFC3 | 29.01 | 27.82 | 6.87 | 18.82 | 16.19 | 14.85 |
| OSGIN1 | 32.67 | 59.11 | 14.62 | 37.55 | 36.62 | 20.68 |
| RTEL1-TNFRSF6B | 24.42 | 42.75 | 10.61 | 25.16 | 47.47 | 17.22 |
| ERCC6L | 33.69 | 33.55 | 8.36 | 20.01 | 18.29 | 17.24 |
| TTK | 60.43 | 58.15 | 14.60 | 55.00 | 33.12 | 38.05 |
| CKAP2L | 74.36 | 69.99 | 17.60 | 93.65 | 54.75 | 54.87 |
| TK1 | 137.07 | 105.20 | 26.65 | 109.76 | 56.07 | 64.44 |
| SLF1 | 32.17 | 31.53 | 8.04 | 28.70 | 22.49 | 14.04 |
| ASPM | 141.23 | 119.15 | 30.40 | 65.58 | 50.43 | 44.66 |
| PCLAF | 100.34 | 84.60 | 21.67 | 76.68 | 47.83 | 56.09 |
| RRM2 | 380.24 | 214.69 | 55.30 | 249.76 | 149.58 | 153.34 |
| MTFR2 | 12.58 | 16.86 | 4.35 | 12.91 | 10.00 | 8.76 |
| SKA3 | 29.13 | 24.52 | 6.35 | 23.07 | 16.12 | 15.97 |
| CIP2A | 95.50 | 102.42 | 26.74 | 89.60 | 64.22 | 52.49 |
| MCM8 | 76.45 | 95.47 | 24.93 | 69.80 | 67.64 | 45.70 |
| WDR76 | 23.26 | 18.62 | 4.87 | 17.51 | 14.73 | 10.14 |
| TOP2A | 533.10 | 477.30 | 125.04 | 280.86 | 195.11 | 237.75 |
| NCAPG | 124.90 | 104.80 | 27.59 | 84.25 | 56.86 | 54.97 |
| RAB8B | 106.36 | 179.22 | 47.30 | 177.53 | 181.69 | 118.07 |
| MELK | 66.62 | 56.39 | 14.90 | 68.45 | 43.39 | 42.76 |
| HJURP | 48.36 | 45.62 | 12.07 | 43.48 | 26.03 | 26.56 |
| PLK4 | 36.90 | 32.04 | 8.49 | 34.74 | 20.49 | 20.70 |
| CDCA2 | 49.29 | 46.65 | 12.38 | 36.77 | 25.66 | 29.62 |
| HASPIN | 18.41 | 18.30 | 4.87 | 21.99 | 14.95 | 14.83 |
| MKI67 | 371.48 | 320.83 | 85.44 | 278.07 | 241.82 | 175.40 |
| SPAG5 | 99.12 | 82.34 | 21.95 | 66.98 | 44.43 | 40.68 |
| PTTG1 | 59.09 | 64.78 | 17.29 | 49.58 | 38.99 | 29.44 |
| EME1 | 12.43 | 10.19 | 2.73 | 10.20 | 6.79 | 7.37 |
| H2AZ1 | 260.00 | 285.84 | 77.37 | 168.98 | 150.06 | 111.26 |
| GNB4 | 117.16 | 180.06 | 48.74 | 180.57 | 214.49 | 118.37 |
| ERI1 | 74.06 | 129.54 | 35.19 | 27.38 | 52.86 | 29.48 |
| SPC25 | 13.60 | 10.97 | 2.98 | 7.29 | 4.94 | 5.59 |
| SNX15 | 31.48 | 27.88 | 7.58 | 32.70 | 34.21 | 11.86 |
| AURKB | 60.16 | 49.95 | 13.59 | 52.23 | 32.42 | 35.11 |
| ATAD2 | 120.08 | 103.98 | 28.60 | 94.25 | 76.23 | 60.91 |
| SGO2 | 57.99 | 61.75 | 17.05 | 37.47 | 29.81 | 23.46 |
| GTSE1 | 63.62 | 49.96 | 13.82 | 52.10 | 29.07 | 31.72 |
| FANCA | 45.56 | 48.89 | 13.53 | 42.95 | 35.13 | 39.68 |
| TSPAN12 | 16.06 | 21.84 | 6.06 | 8.35 | 16.18 | 5.78 |
| TRAIP | 10.22 | 9.67 | 2.69 | 10.23 | 6.93 | 7.98 |
| TXN | 231.68 | 348.24 | 97.19 | 214.71 | 275.30 | 101.46 |
| ZGRF1 | 20.08 | 16.55 | 4.62 | 21.25 | 13.86 | 14.42 |
| KIF23 | 131.51 | 148.24 | 41.42 | 83.70 | 68.42 | 68.28 |
| BUB1 | 108.84 | 85.70 | 24.08 | 103.05 | 63.47 | 66.87 |
| RAD54B | 15.03 | 18.70 | 5.26 | 11.48 | 11.62 | 9.63 |
| RPL39L | 14.28 | 10.88 | 3.07 | 11.26 | 8.70 | 6.11 |
| AGO2 | 126.50 | 213.32 | 60.14 | 68.58 | 106.68 | 44.22 |
| CDCA5 | 79.60 | 67.91 | 19.18 | 74.90 | 49.17 | 51.41 |
| AURKA | 135.34 | 160.31 | 45.40 | 97.92 | 84.16 | 74.03 |
| ZNF367 | 37.57 | 36.84 | 10.44 | 35.30 | 21.45 | 21.29 |
| CLSPN | 114.37 | 97.29 | 27.77 | 134.06 | 97.76 | 81.83 |
| DBF4 | 51.27 | 69.46 | 19.96 | 40.67 | 38.55 | 24.80 |
| BUB1B | 85.34 | 66.39 | 19.12 | 47.34 | 37.21 | 38.01 |
| RFC5 | 48.79 | 53.00 | 15.28 | 31.98 | 31.71 | 21.94 |
| NCAPG2 | 126.50 | 103.51 | 29.86 | 76.94 | 63.05 | 59.22 |
| CBX3 | 230.54 | 260.44 | 75.25 | 203.75 | 200.63 | 105.42 |
| TUBB4B | 679.89 | 858.69 | 250.30 | 558.31 | 533.97 | 509.99 |
| MCM4 | 191.85 | 170.10 | 49.62 | 146.82 | 107.74 | 106.45 |
| FIGNL1 | 44.52 | 44.18 | 12.93 | 32.81 | 30.97 | 23.10 |
| KIF20A | 157.91 | 116.85 | 34.24 | 86.90 | 50.24 | 50.02 |
| DNA2 | 27.87 | 31.20 | 9.15 | 20.73 | 17.58 | 15.73 |
| SMC4 | 571.57 | 577.50 | 169.52 | 350.52 | 306.52 | 247.74 |
| CENPK | 34.67 | 36.18 | 10.67 | 30.58 | 23.38 | 25.19 |
| PRIM2 | 44.56 | 38.50 | 11.38 | 47.90 | 34.79 | 26.41 |
| KIF15 | 46.38 | 32.72 | 9.69 | 26.27 | 16.47 | 16.49 |
| PSRC1 | 14.20 | 14.59 | 4.32 | 9.05 | 8.05 | 6.59 |
| CENPQ | 13.34 | 19.19 | 5.71 | 16.47 | 13.81 | 10.88 |
| UBE2S | 243.38 | 235.18 | 70.10 | 186.61 | 169.23 | 102.01 |
| GINS2 | 36.69 | 24.94 | 7.44 | 24.51 | 17.32 | 16.08 |
| ACYP1 | 10.74 | 14.40 | 4.30 | 8.20 | 11.88 | 5.54 |
| MIS18A | 20.61 | 22.04 | 6.59 | 17.90 | 14.39 | 12.49 |
| CDCA3 | 26.58 | 22.77 | 6.81 | 17.81 | 11.60 | 12.65 |
| ALG10 | 19.28 | 18.50 | 5.57 | 16.33 | 12.33 | 9.81 |
| KIF11 | 168.08 | 152.55 | 46.13 | 126.17 | 83.45 | 87.37 |
| DBF4B | 22.17 | 24.89 | 7.54 | 45.80 | 26.61 | 30.26 |
| KIF14 | 72.07 | 59.55 | 18.12 | 56.66 | 36.56 | 36.40 |
| MTBP | 17.58 | 14.47 | 4.40 | 18.90 | 11.67 | 10.58 |
| CENPU | 35.74 | 31.67 | 9.64 | 25.92 | 17.18 | 19.78 |
| WDR62 | 34.28 | 29.71 | 9.05 | 31.18 | 20.76 | 21.13 |
| SUV39H1 | 26.49 | 31.84 | 9.71 | 20.69 | 23.22 | 15.96 |
| FEN1 | 147.97 | 157.50 | 48.09 | 121.95 | 101.91 | 100.46 |
| DTL | 75.84 | 54.27 | 16.57 | 76.90 | 46.45 | 56.19 |
| TICRR | 35.67 | 36.23 | 11.07 | 39.56 | 36.08 | 32.75 |
| DLGAP5 | 146.12 | 114.48 | 34.99 | 79.64 | 56.37 | 59.03 |
| KIF4A | 104.58 | 84.02 | 25.69 | 67.03 | 46.74 | 44.28 |
| CDT1 | 56.60 | 40.66 | 12.48 | 58.86 | 33.20 | 29.82 |
| BCL2L12 | 17.39 | 25.46 | 7.82 | 14.40 | 16.25 | 12.69 |
| RFC4 | 31.31 | 26.05 | 8.01 | 20.30 | 17.67 | 13.54 |
| CIT | 100.49 | 90.68 | 27.90 | 73.44 | 59.30 | 49.65 |
| CSTF2 | 65.88 | 85.51 | 26.36 | 63.56 | 82.03 | 34.49 |
| SASS6 | 15.90 | 18.98 | 5.86 | 20.69 | 11.39 | 13.83 |
| ORC6 | 42.52 | 33.20 | 10.28 | 48.37 | 34.02 | 30.86 |
| TCF19 | 36.84 | 17.68 | 5.49 | 25.28 | 18.03 | 16.69 |
| ENSG00000284946 | 28.72 | 26.55 | 8.25 | 21.74 | 17.14 | 23.05 |
| PIMREG | 22.62 | 21.08 | 6.56 | 12.09 | 10.40 | 10.98 |
| ARHGAP11A | 174.28 | 190.62 | 59.55 | 112.51 | 99.96 | 85.83 |
| FBXO5 | 36.78 | 35.87 | 11.25 | 33.79 | 24.27 | 20.13 |
| TROAP | 40.18 | 31.67 | 9.93 | 42.61 | 23.68 | 20.15 |
| CHTF18 | 42.57 | 36.56 | 11.47 | 38.07 | 26.82 | 28.67 |
| ATAD5 | 32.51 | 32.14 | 10.08 | 28.09 | 21.45 | 21.23 |
| CENPW | 16.59 | 14.43 | 4.53 | 9.73 | 7.44 | 7.24 |
| RTTN | 17.77 | 20.18 | 6.33 | 18.10 | 13.85 | 9.81 |
| CEP55 | 116.72 | 90.75 | 28.49 | 70.39 | 56.68 | 48.15 |
| C1orf112 | 22.27 | 22.31 | 7.03 | 21.58 | 14.45 | 14.94 |
| CCNE2 | 14.70 | 12.83 | 4.05 | 12.47 | 9.29 | 8.59 |
| CDC45 | 31.67 | 24.59 | 7.79 | 25.50 | 16.36 | 16.36 |
| CCNB2 | 58.81 | 42.10 | 13.36 | 30.86 | 19.76 | 20.96 |
| MCM2 | 153.35 | 122.31 | 38.98 | 122.27 | 82.07 | 71.16 |
| RAD18 | 46.48 | 62.41 | 19.91 | 34.29 | 38.60 | 28.28 |
| C18orf54 | 34.60 | 35.34 | 11.30 | 27.85 | 25.13 | 20.32 |
| VRK1 | 50.43 | 55.96 | 17.94 | 42.47 | 37.48 | 33.92 |
| NQO1 | 606.14 | 703.72 | 225.93 | 374.62 | 346.58 | 159.70 |
| RAD51 | 13.82 | 14.54 | 4.68 | 10.64 | 9.49 | 9.38 |
| PRC1 | 147.60 | 98.06 | 31.62 | 95.95 | 61.89 | 53.84 |
| H2AX | 179.60 | 183.36 | 59.37 | 135.62 | 102.74 | 90.61 |
| LRR1 | 27.90 | 34.36 | 11.13 | 26.47 | 25.98 | 17.93 |
| PARPBP | 42.10 | 43.65 | 14.18 | 26.81 | 22.86 | 19.40 |
| CHAC2 | 14.60 | 18.55 | 6.03 | 11.71 | 11.70 | 7.87 |
| RECQL4 | 39.75 | 35.32 | 11.49 | 33.08 | 24.80 | 26.37 |
| SMS | 204.63 | 252.63 | 82.17 | 159.25 | 216.22 | 102.06 |
| GLUD1 | 269.56 | 342.46 | 111.54 | 228.20 | 231.36 | 128.53 |
| MORF4L2 | 788.40 | 945.67 | 308.07 | 741.09 | 785.62 | 359.11 |
| TRIM16L | 22.17 | 30.03 | 9.81 | 25.12 | 18.57 | 15.52 |
| POLA2 | 40.04 | 25.74 | 8.41 | 44.62 | 25.13 | 35.98 |
| TEDC2 | 10.22 | 8.03 | 2.64 | 7.38 | 6.80 | 5.50 |
| SERPINB2 | 121.46 | 170.32 | 56.06 | 605.45 | 899.48 | 156.34 |
| BRIP1 | 60.26 | 55.34 | 18.26 | 136.17 | 69.69 | 109.09 |
| BLM | 41.42 | 34.90 | 11.54 | 27.69 | 20.15 | 21.93 |
| TXNRD1 | 1477.55 | 2018.34 | 667.16 | 1295.44 | 1407.12 | 609.30 |
| DHFR | 84.57 | 44.94 | 14.87 | 42.83 | 26.96 | 28.10 |
| OAS3 | 51.11 | 72.45 | 24.06 | 1765.41 | 1313.82 | 1588.61 |
| FANCI | 125.70 | 100.83 | 33.72 | 131.56 | 79.63 | 94.14 |
| GINS1 | 77.95 | 78.49 | 26.31 | 89.80 | 56.99 | 75.58 |
| KNL1 | 88.77 | 71.33 | 23.95 | 53.36 | 41.89 | 43.18 |
| IQGAP3 | 47.04 | 35.53 | 11.93 | 36.08 | 20.27 | 25.56 |
| NMRAL2P | 10.96 | 11.52 | 3.87 | 24.05 | 12.59 | 7.66 |
| GMNN | 22.65 | 25.77 | 8.67 | 23.35 | 19.63 | 15.72 |
| SNRNP40 | 63.56 | 65.99 | 22.29 | 59.22 | 53.96 | 31.12 |
| RAD54L | 17.27 | 14.18 | 4.83 | 20.49 | 12.64 | 16.84 |
| KIF22 | 80.54 | 80.71 | 27.55 | 65.56 | 49.50 | 45.75 |
| TOPBP1 | 91.79 | 97.35 | 33.33 | 67.42 | 70.13 | 43.56 |
| ORC1 | 28.65 | 18.08 | 6.21 | 28.43 | 18.09 | 16.39 |
| C4orf46 | 45.12 | 40.68 | 13.99 | 36.79 | 26.19 | 23.83 |
| QSER1 | 139.48 | 170.22 | 58.74 | 88.72 | 126.97 | 64.61 |
| DGAT2 | 19.43 | 24.23 | 8.37 | 5.84 | 15.56 | 8.26 |
| FLVCR1 | 19.58 | 18.21 | 6.30 | 14.11 | 15.70 | 7.06 |
| CENPJ | 16.50 | 19.15 | 6.66 | 12.07 | 10.65 | 8.38 |
| FOXM1 | 209.25 | 173.47 | 60.49 | 169.23 | 115.02 | 100.85 |
| BRCA2 | 39.08 | 31.82 | 11.11 | 49.65 | 35.72 | 32.59 |
| TMEM106C | 90.50 | 76.88 | 26.92 | 60.39 | 52.82 | 36.66 |
| HELLS | 62.11 | 65.58 | 22.98 | 51.13 | 42.87 | 42.83 |
| ECT2 | 240.48 | 267.35 | 94.29 | 237.82 | 188.14 | 155.01 |
| MAGEA12 | 47.55 | 47.50 | 16.86 | 47.69 | 47.26 | 26.09 |
| POLE2 | 20.41 | 15.55 | 5.53 | 11.42 | 8.24 | 7.46 |
| GAS2L3 | 59.35 | 64.45 | 22.94 | 45.46 | 33.67 | 24.08 |
| NUSAP1 | 83.51 | 64.19 | 22.86 | 54.45 | 27.02 | 38.86 |
| HAUS3 | 32.88 | 36.71 | 13.12 | 25.63 | 26.13 | 22.87 |
| GINS4 | 34.57 | 21.51 | 7.69 | 19.42 | 13.08 | 13.73 |
| NR2C2AP | 15.15 | 16.14 | 5.78 | 13.46 | 14.31 | 9.25 |
| KIF20B | 92.18 | 71.31 | 25.56 | 76.92 | 44.81 | 55.72 |
| TMPO | 300.89 | 279.60 | 100.36 | 268.01 | 206.56 | 175.13 |
| H1-0 | 273.95 | 370.36 | 133.06 | 643.52 | 458.02 | 274.90 |
| LRRC58 | 202.21 | 219.74 | 79.01 | 135.06 | 197.31 | 86.22 |
| CYFIP1 | 252.77 | 285.52 | 103.63 | 246.65 | 262.51 | 145.00 |
| KNTC1 | 70.23 | 66.38 | 24.15 | 58.55 | 45.07 | 38.68 |
| ATP6V0E2 | 48.02 | 52.84 | 19.38 | 27.69 | 42.36 | 16.31 |
| CHAF1A | 80.89 | 54.94 | 20.19 | 75.59 | 55.78 | 45.88 |
| TEDC1 | 29.70 | 34.88 | 12.83 | 24.08 | 25.83 | 20.14 |
| VAMP3 | 128.25 | 137.68 | 51.03 | 113.71 | 125.03 | 68.54 |
| DCK | 50.93 | 54.71 | 20.31 | 38.91 | 41.45 | 22.68 |
| BARD1 | 18.05 | 17.73 | 6.59 | 24.02 | 17.04 | 17.29 |
| DNAJC9 | 59.12 | 65.15 | 24.23 | 49.36 | 44.96 | 37.92 |
| TMOD3 | 194.19 | 237.52 | 88.63 | 220.09 | 225.50 | 139.63 |
| SAPCD2 | 110.40 | 77.69 | 29.03 | 50.20 | 55.86 | 38.03 |
| CENPE | 140.20 | 97.93 | 36.63 | 109.44 | 72.95 | 64.12 |
| CENPA | 22.26 | 11.54 | 4.32 | 15.58 | 8.49 | 10.00 |
| CKS1B | 81.30 | 86.09 | 32.49 | 101.77 | 67.24 | 68.25 |
| FANCM | 29.21 | 35.46 | 13.44 | 26.07 | 24.99 | 25.19 |
| RMI2 | 26.44 | 21.80 | 8.27 | 41.71 | 24.31 | 31.62 |
| NCAPD3 | 120.73 | 100.45 | 38.12 | 81.88 | 74.18 | 67.51 |
| E2F8 | 23.79 | 11.52 | 4.37 | 20.57 | 10.66 | 9.36 |
| C5orf34 | 13.86 | 11.71 | 4.45 | 14.55 | 9.20 | 10.23 |
| WDHD1 | 73.29 | 55.38 | 21.05 | 51.37 | 37.65 | 40.79 |
| MCM5 | 112.02 | 96.39 | 36.67 | 98.44 | 72.16 | 59.21 |
| C12orf75 | 62.62 | 53.53 | 20.48 | 44.34 | 49.92 | 28.67 |
| BRCC3 | 34.37 | 34.71 | 13.29 | 27.86 | 29.65 | 18.54 |
| GCLM | 139.68 | 148.70 | 57.14 | 154.90 | 145.08 | 87.49 |
| E2F1 | 72.01 | 38.86 | 14.94 | 43.53 | 26.02 | 25.91 |
| EMP1 | 494.46 | 566.01 | 217.75 | 388.27 | 326.93 | 210.52 |
| MCM7 | 240.00 | 200.06 | 77.07 | 199.93 | 144.93 | 129.87 |
| KIF18A | 58.59 | 49.12 | 18.95 | 48.14 | 29.29 | 36.54 |
| GEN1 | 30.44 | 35.90 | 13.87 | 30.26 | 25.96 | 24.57 |
| STIL | 64.87 | 57.84 | 22.39 | 81.40 | 47.27 | 60.11 |
| DIAPH3 | 55.92 | 43.48 | 16.86 | 38.66 | 23.16 | 29.82 |
| POC1A | 22.54 | 18.14 | 7.06 | 17.12 | 12.17 | 12.53 |
| EPS15 | 89.72 | 91.37 | 35.60 | 113.24 | 90.42 | 54.23 |
| LMNB1 | 191.76 | 174.94 | 68.41 | 121.29 | 117.90 | 87.09 |
| CKAP2 | 99.35 | 93.11 | 36.60 | 93.17 | 61.79 | 62.15 |
| DSN1 | 76.75 | 59.70 | 23.50 | 64.29 | 47.12 | 43.53 |
| NUDT1 | 13.77 | 15.88 | 6.27 | 10.58 | 11.04 | 5.91 |
| GLYR1 | 118.56 | 141.66 | 56.55 | 112.38 | 120.81 | 69.96 |
| LASP1 | 513.53 | 593.88 | 238.44 | 328.85 | 449.16 | 242.28 |
| CENPI | 75.66 | 47.63 | 19.13 | 36.55 | 27.18 | 30.52 |
| CENPX | 26.39 | 32.20 | 12.96 | 19.00 | 22.88 | 16.92 |
| TPX2 | 470.45 | 350.91 | 141.37 | 338.44 | 234.83 | 238.48 |
| USP1 | 126.00 | 119.64 | 48.52 | 119.58 | 87.88 | 75.02 |
| PRTFDC1 | 10.90 | 12.65 | 5.15 | 8.43 | 5.83 | 6.53 |
| RNASEH2A | 42.75 | 29.49 | 12.00 | 32.97 | 24.02 | 20.98 |
| CBR3 | 15.05 | 14.37 | 5.85 | 26.21 | 15.45 | 13.71 |
| ENSG00000290318 | 24.37 | 21.85 | 8.97 | 11.75 | 16.78 | 14.25 |
| NUP107 | 132.75 | 136.16 | 55.99 | 110.18 | 90.80 | 90.36 |
| MASTL | 39.66 | 39.81 | 16.38 | 117.62 | 64.91 | 89.25 |
| DTYMK | 31.30 | 28.45 | 11.71 | 18.71 | 18.28 | 14.23 |
| ESYT2 | 270.40 | 300.96 | 124.11 | 219.30 | 182.43 | 135.19 |
| ENSG00000284906 | 19.50 | 14.82 | 6.14 | 23.32 | 12.89 | 16.14 |
| ENSG00000284969 | 21.54 | 25.22 | 10.49 | 14.07 | 21.78 | 8.63 |
| GSTCD | 25.05 | 26.23 | 10.94 | 21.04 | 16.64 | 16.69 |
| LSM5 | 35.74 | 39.31 | 16.41 | 29.88 | 24.61 | 20.28 |
| CDC6 | 82.08 | 63.89 | 26.74 | 89.45 | 44.97 | 55.20 |
| HMGB2 | 141.98 | 124.10 | 52.04 | 141.07 | 75.75 | 91.35 |
| HMMR | 105.03 | 74.23 | 31.16 | 80.39 | 41.52 | 52.02 |
| EID2 | 19.25 | 20.41 | 8.58 | 15.33 | 17.14 | 9.77 |
| XRCC2 | 32.42 | 18.78 | 7.90 | 20.96 | 16.71 | 19.80 |
| UHRF1 | 226.03 | 189.47 | 79.94 | 162.38 | 138.64 | 115.68 |
| CENPH | 16.54 | 11.34 | 4.79 | 16.89 | 7.40 | 10.06 |
| DCTN3 | 23.51 | 24.91 | 10.53 | 17.10 | 20.81 | 12.89 |
| PCNA | 431.48 | 366.51 | 155.72 | 368.40 | 306.58 | 275.78 |
| FTO | 49.44 | 44.85 | 19.09 | 43.81 | 41.13 | 26.15 |
| MMS22L | 52.43 | 38.90 | 16.62 | 44.03 | 30.49 | 27.37 |
| PRR11 | 219.57 | 180.64 | 77.18 | 105.06 | 77.32 | 119.01 |
| POLE | 52.78 | 50.44 | 21.65 | 40.01 | 38.07 | 36.78 |
| MCM6 | 136.07 | 111.13 | 47.82 | 89.92 | 64.45 | 54.62 |
| FGD4 | 115.38 | 127.01 | 54.84 | 93.51 | 75.94 | 41.60 |
| POLA1 | 48.43 | 44.82 | 19.37 | 36.01 | 34.25 | 26.73 |
| CCDC138 | 10.49 | 11.93 | 5.20 | 7.58 | 6.57 | 6.07 |
| ZWINT | 77.36 | 45.32 | 19.94 | 57.49 | 38.67 | 39.02 |
| SEC61A1 | 340.86 | 336.23 | 148.09 | 454.34 | 416.75 | 202.31 |
| ENSG00000256663 | 18.11 | 14.97 | 6.62 | 13.14 | 10.91 | 9.11 |
| STK38L | 103.12 | 113.22 | 50.05 | 77.21 | 89.97 | 32.60 |
| NCAPH2 | 43.70 | 36.98 | 16.38 | 48.05 | 38.91 | 34.14 |
| PPIH | 24.94 | 25.17 | 11.15 | 22.46 | 17.71 | 18.80 |
| MYBL2 | 90.87 | 57.11 | 25.32 | 66.85 | 36.22 | 54.09 |
| STT3B | 412.31 | 388.20 | 172.57 | 440.96 | 437.45 | 311.36 |
| TONSL | 33.18 | 24.68 | 10.98 | 28.20 | 23.73 | 23.74 |
| DCLRE1B | 24.33 | 18.85 | 8.40 | 20.46 | 17.24 | 13.08 |
| TMEM158 | 94.76 | 96.22 | 42.90 | 122.93 | 170.92 | 58.32 |
| TACC3 | 162.02 | 84.63 | 37.77 | 123.60 | 80.90 | 79.70 |
| DOCK7 | 63.36 | 66.52 | 29.78 | 41.40 | 51.86 | 26.94 |
| RRM1 | 209.87 | 146.41 | 65.57 | 126.56 | 96.82 | 98.70 |
| PI4K2B | 55.88 | 50.72 | 22.74 | 145.48 | 88.53 | 84.28 |
| LIN9 | 18.84 | 18.97 | 8.52 | 15.19 | 12.55 | 11.35 |
| CENPP | 15.71 | 14.04 | 6.32 | 11.10 | 10.42 | 10.50 |
| SHMT1 | 26.30 | 22.23 | 10.01 | 16.28 | 15.25 | 12.06 |
| CEBPG | 55.93 | 52.22 | 23.52 | 84.34 | 63.00 | 34.18 |
| DDX39A | 94.78 | 103.32 | 46.57 | 79.93 | 80.36 | 53.24 |
| USP13 | 48.44 | 49.09 | 22.15 | 31.75 | 45.76 | 22.70 |
| ASNS | 33.66 | 29.62 | 13.46 | 70.23 | 24.92 | 13.50 |
| BORA | 10.42 | 8.11 | 3.69 | 11.57 | 6.53 | 6.69 |
| PLEKHB2 | 193.32 | 124.33 | 56.98 | 208.43 | 229.20 | 103.16 |
| PSAT1 | 77.10 | 66.67 | 30.70 | 101.37 | 46.38 | 28.82 |
| BIRC5 | 144.63 | 63.00 | 29.02 | 69.47 | 39.98 | 53.63 |
| HMGB3 | 74.10 | 50.40 | 23.35 | 30.31 | 23.36 | 15.83 |
| GPSM2 | 34.20 | 35.31 | 16.44 | 15.09 | 15.32 | 11.50 |
| WRAP53 | 30.19 | 29.66 | 13.84 | 25.47 | 24.27 | 21.51 |
| DEK | 219.56 | 201.86 | 94.34 | 220.23 | 168.69 | 152.16 |
| ALG10B | 24.79 | 19.63 | 9.19 | 17.15 | 14.27 | 11.26 |
| FGF5 | 123.38 | 111.07 | 52.13 | 427.56 | 275.51 | 184.11 |
| H1-10 | 109.94 | 96.32 | 45.31 | 117.28 | 81.00 | 71.88 |
| UBE2T | 32.72 | 30.37 | 14.33 | 29.26 | 21.00 | 18.73 |
| HAUS6 | 74.30 | 71.48 | 33.77 | 55.16 | 54.71 | 43.13 |
| PHGDH | 51.87 | 31.41 | 14.88 | 63.68 | 21.92 | 19.39 |
| PM20D2 | 92.65 | 77.32 | 36.72 | 53.07 | 43.15 | 34.09 |
| CEP152 | 16.33 | 16.76 | 7.99 | 22.76 | 14.30 | 12.59 |
| ATP6AP2 | 148.93 | 152.83 | 73.02 | 123.10 | 128.71 | 95.14 |
| PRIM1 | 56.39 | 36.59 | 17.49 | 32.54 | 22.55 | 25.47 |
| ENSG00000289194 | 11.93 | 7.19 | 3.45 | 7.85 | 4.45 | 5.10 |
| LIG1 | 50.41 | 43.50 | 20.89 | 40.42 | 36.54 | 31.99 |
| MRE11 | 58.63 | 54.87 | 26.49 | 44.82 | 38.79 | 37.38 |
| NEMP1 | 85.90 | 69.97 | 33.81 | 146.11 | 76.03 | 87.29 |
| RMI1 | 27.94 | 26.64 | 12.94 | 31.41 | 20.36 | 23.19 |
| FAM111A | 111.83 | 95.23 | 46.34 | 241.37 | 150.40 | 193.99 |
| GGH | 95.77 | 92.89 | 45.31 | 69.93 | 65.46 | 55.24 |
| MIR100HG | 32.39 | 33.00 | 16.12 | 75.13 | 50.43 | 28.91 |
| CENPN | 97.85 | 76.37 | 37.60 | 87.21 | 61.33 | 67.84 |
| CENPO | 44.50 | 40.50 | 20.00 | 36.26 | 33.27 | 34.76 |
| CHAF1B | 35.47 | 22.52 | 11.13 | 30.51 | 19.00 | 18.72 |
| SMC2 | 211.87 | 181.28 | 90.05 | 131.90 | 107.32 | 98.66 |
