# Supplementary material for: Downregulation of Engulfment and cell motility 1 (Elmo1) induces quiescence and resistance to poly(I:C)-induced apoptosis in endothelial cells
Source: Cell Death Dis. 2025 Dec 20;17(1):100. doi: 10.1038/s41419-025-08341-1 (PMC12847878; doi:10.1038/s41419-025-08341-1)
Supplement: Supplementary file 11 — Supplementary Table S3 [file 41419_2025_8341_MOESM11_ESM.docx]

| **Table S3. Genes expressed higher in siELMO1 at 24 h** | | | |  |  |  |
| --- | --- | --- | --- | --- | --- | --- |
| Gene | 0H_Mock | 0H_siNT | 0H_siELMO1 | 24H_Mock | 24H_siNT | 24H_siELMO1 |
| COL5A1 | 120.27 | 70.35 | 885.61 | 115.96 | 62.36 | 1091.62 |
| CSF2 | 0.48 | 1.33 | 0.75 | 8.52 | 1.76 | 28.78 |
| LTB | 0.11 | 0.19 | 0.36 | 4.23 | 1.02 | 12.42 |
| EFNA1 | 0.33 | 0.26 | 1.50 | 6.43 | 1.65 | 18.93 |
| MT-TF | 0.62 | 0.61 | 0.93 | 10.11 | 2.69 | 30.12 |
| PPP1R16B | 4.44 | 2.05 | 18.71 | 1.87 | 1.44 | 13.60 |
| LTBP1 | 40.31 | 16.21 | 90.93 | 32.77 | 12.60 | 104.61 |
| FSTL5 | 24.67 | 7.34 | 53.82 | 19.14 | 7.75 | 57.03 |
| TNFSF15 | 26.83 | 21.21 | 63.21 | 22.52 | 26.45 | 190.72 |
| THBS1 | 1436.17 | 1192.24 | 4842.05 | 1322.20 | 648.25 | 3964.99 |
| COL4A1 | 35.47 | 23.04 | 102.14 | 118.98 | 53.55 | 321.48 |
| VWF | 5.58 | 3.77 | 13.03 | 9.80 | 4.63 | 26.88 |
| MRC2 | 6.65 | 2.92 | 11.61 | 5.87 | 2.56 | 13.91 |
| DYSF | 50.32 | 20.78 | 141.65 | 49.23 | 27.74 | 148.73 |
| ADAMTS7 | 22.09 | 17.55 | 59.28 | 17.76 | 15.75 | 82.00 |
| GDF6 | 17.56 | 6.73 | 39.39 | 5.61 | 5.91 | 29.41 |
| BFSP1 | 9.93 | 4.41 | 26.34 | 8.32 | 4.65 | 23.04 |
| SGIP1 | 4.28 | 2.83 | 7.21 | 6.83 | 2.98 | 13.88 |
| MMP2 | 439.28 | 393.38 | 1324.83 | 305.39 | 290.86 | 1334.13 |
| PDGFB | 17.87 | 14.62 | 52.97 | 15.47 | 11.61 | 52.16 |
| EDN1 | 95.11 | 51.77 | 190.26 | 76.68 | 39.39 | 174.25 |
| EFEMP1 | 1464.34 | 909.13 | 2726.32 | 1143.78 | 606.10 | 2669.11 |
| LINC02915 | 38.23 | 14.84 | 56.95 | 19.97 | 13.25 | 57.92 |
| LRRC32 | 1.20 | 0.55 | 2.24 | 3.57 | 2.20 | 9.25 |
| DKK3 | 35.62 | 28.45 | 81.21 | 50.59 | 34.95 | 138.01 |
| COL4A2 | 23.07 | 21.21 | 46.61 | 41.26 | 28.33 | 111.40 |
| DNAH8 | 2.98 | 2.17 | 8.28 | 1.30 | 1.68 | 6.42 |
| ITGB8 | 22.50 | 15.18 | 66.62 | 11.85 | 13.90 | 49.89 |
| MOV10L1 | 1.14 | 0.53 | 3.55 | 1.12 | 1.05 | 3.69 |
| FAP | 53.63 | 33.44 | 81.49 | 32.70 | 19.76 | 68.84 |
| CUBN | 7.38 | 5.10 | 15.10 | 5.43 | 5.98 | 20.44 |
| COL13A1 | 12.11 | 4.96 | 25.21 | 5.82 | 5.83 | 19.92 |
| F11R | 13.38 | 16.52 | 36.59 | 26.52 | 20.92 | 71.42 |
| SH2D3C | 2.59 | 1.23 | 8.66 | 1.95 | 2.18 | 7.43 |
| BMF | 3.08 | 4.55 | 13.02 | 5.25 | 4.11 | 13.98 |
| CCDC80 | 274.86 | 87.45 | 581.84 | 201.96 | 128.15 | 433.18 |
| CPA3 | 3.93 | 5.77 | 15.72 | 2.40 | 2.73 | 9.16 |
| VSIG1 | 4.18 | 7.17 | 7.53 | 2.22 | 2.26 | 7.59 |
| SIRPB2 | 14.34 | 6.05 | 32.78 | 19.31 | 14.45 | 48.22 |
| ANGPTL2 | 9.29 | 8.47 | 17.31 | 18.65 | 11.76 | 39.22 |
| YPEL2 | 22.43 | 21.24 | 63.46 | 25.65 | 19.97 | 66.41 |
| ATOH8 | 5.91 | 10.07 | 11.28 | 3.82 | 3.49 | 11.55 |
| STXBP5-AS1 | 2.17 | 1.31 | 4.29 | 1.83 | 1.57 | 5.15 |
| ROR1 | 18.92 | 7.76 | 38.95 | 7.49 | 6.73 | 21.80 |
| MR1 | 3.27 | 3.09 | 10.95 | 8.94 | 7.01 | 22.20 |
| ENSG00000260604 | 12.08 | 5.76 | 21.33 | 2.90 | 2.93 | 9.23 |
| FRAS1 | 4.03 | 7.19 | 18.62 | 4.90 | 9.50 | 29.74 |
| EXOC3L1 | 0.49 | 0.38 | 1.68 | 2.32 | 2.12 | 6.61 |
| PLXNA4 | 67.41 | 46.00 | 123.92 | 16.83 | 18.20 | 56.25 |
| NTM | 13.14 | 5.08 | 17.71 | 17.41 | 12.00 | 36.41 |
| CHSY3 | 2.23 | 1.37 | 5.57 | 3.82 | 2.54 | 7.65 |
| PALM | 2.12 | 1.25 | 4.80 | 2.13 | 1.63 | 4.88 |
| MEX3A | 11.03 | 6.10 | 26.23 | 6.59 | 5.25 | 15.34 |
| DENND2B | 11.09 | 15.92 | 27.88 | 8.45 | 9.76 | 28.29 |
| SORBS2 | 6.84 | 6.01 | 13.91 | 1.65 | 2.48 | 7.19 |
| GPR153 | 8.32 | 6.06 | 19.77 | 7.95 | 7.56 | 21.85 |
| JUP | 41.00 | 100.72 | 115.28 | 14.82 | 21.69 | 62.55 |
| PTGFRN | 21.02 | 16.02 | 38.82 | 10.19 | 11.83 | 34.08 |
| CLEC7A | 0.28 | 0.13 | 1.01 | 6.65 | 5.82 | 16.72 |
| CITED4 | 38.85 | 30.44 | 62.01 | 60.95 | 48.35 | 138.44 |
| ABCA8 | 5.99 | 5.27 | 16.33 | 2.22 | 2.46 | 7.02 |
| MMRN1 | 24.58 | 14.80 | 41.93 | 18.08 | 16.13 | 45.79 |
| ERV3-1 | 1.66 | 1.55 | 3.29 | 1.21 | 1.19 | 3.34 |
| NID1 | 20.13 | 33.26 | 58.35 | 30.04 | 35.62 | 99.15 |
| STAB1 | 103.08 | 96.92 | 179.05 | 68.80 | 85.81 | 237.54 |
| GAS6 | 7.44 | 3.60 | 9.76 | 8.63 | 6.32 | 17.49 |
| TUBA4A | 2.09 | 1.93 | 1.92 | 1.19 | 1.08 | 2.98 |
| F2R | 218.03 | 163.86 | 437.13 | 82.44 | 100.91 | 276.73 |
| TXNDC5 | 301.33 | 256.20 | 552.42 | 165.02 | 170.09 | 465.42 |
| LINC02709 | 1.78 | 2.91 | 4.58 | 1.19 | 1.53 | 4.17 |
| PLOD1 | 214.93 | 139.05 | 324.65 | 206.72 | 157.12 | 425.95 |
| ENSG00000286508 | 1.55 | 1.45 | 2.75 | 2.00 | 1.80 | 4.72 |
| SELP | 2.55 | 4.36 | 7.93 | 1.89 | 4.35 | 11.33 |
| KCNG1 | 2.95 | 2.65 | 5.79 | 2.80 | 2.33 | 6.03 |
| SERINC2 | 5.14 | 7.64 | 10.69 | 3.85 | 3.10 | 7.92 |
| PRSS23 | 1329.48 | 1478.27 | 2303.63 | 1269.71 | 1007.59 | 2559.36 |
| STARD5 | 0.55 | 1.67 | 0.57 | 1.64 | 2.01 | 5.09 |
| RASGRF1 | 1.21 | 1.53 | 1.47 | 2.68 | 2.36 | 5.92 |
| THSD4 | 54.65 | 46.15 | 130.10 | 49.75 | 50.84 | 127.49 |
| VIM-AS1 | 5.66 | 6.64 | 7.11 | 2.91 | 2.68 | 6.65 |
| MMRN2 | 145.96 | 153.98 | 303.16 | 69.83 | 107.12 | 264.56 |
| FAM234A | 48.23 | 39.71 | 81.98 | 49.73 | 40.50 | 99.92 |
| HTR1D | 16.90 | 7.93 | 34.55 | 4.45 | 3.91 | 9.57 |
| UCP2 | 14.44 | 10.06 | 31.93 | 9.46 | 8.03 | 19.55 |
| FBN1 | 86.61 | 133.80 | 226.33 | 118.56 | 136.51 | 331.95 |
| RHOBTB1 | 11.87 | 9.21 | 32.44 | 4.83 | 4.92 | 11.94 |
| CABLES1 | 59.17 | 27.75 | 82.21 | 12.94 | 13.64 | 33.06 |
| SLC37A2 | 19.89 | 17.64 | 37.39 | 11.09 | 9.36 | 22.28 |
| FN1 | 502.28 | 548.76 | 1032.18 | 886.59 | 945.25 | 2243.50 |
| SLC22A4 | 4.49 | 3.15 | 9.15 | 5.85 | 5.17 | 12.25 |
| GALNT1 | 202.90 | 152.56 | 384.41 | 134.83 | 138.83 | 326.12 |
| LAMA4 | 799.58 | 410.49 | 1369.98 | 668.21 | 591.98 | 1379.92 |
| USHBP1 | 0.57 | 1.91 | 1.88 | 1.62 | 1.84 | 4.28 |
| FAM43A | 35.47 | 53.11 | 77.93 | 43.69 | 55.88 | 130.00 |
| IGFBP7 | 199.69 | 195.48 | 328.85 | 174.50 | 156.26 | 363.32 |
| KDR | 5.18 | 7.03 | 21.70 | 5.86 | 12.09 | 27.32 |
| RAET1E | 0.64 | 3.29 | 0.60 | 1.07 | 2.17 | 4.91 |
| RALA | 75.69 | 88.05 | 147.53 | 82.06 | 73.25 | 165.16 |
| LINC01013 | 2.31 | 1.33 | 1.79 | 1.98 | 1.98 | 4.44 |
| SVIL | 117.78 | 93.39 | 169.22 | 68.54 | 72.12 | 162.01 |
| WIPF1 | 16.54 | 15.62 | 35.87 | 31.95 | 30.82 | 68.65 |
| EBI3 | 4.02 | 5.61 | 6.96 | 18.31 | 18.07 | 40.21 |
| PCYOX1L | 7.70 | 6.22 | 12.33 | 4.39 | 4.25 | 9.43 |
| SEZ6L2 | 79.37 | 73.03 | 141.71 | 109.54 | 100.56 | 221.88 |
| GPR68 | 1.49 | 0.94 | 2.66 | 3.44 | 3.42 | 7.50 |
| ENSG00000287774 | 1.68 | 3.05 | 3.37 | 4.82 | 4.89 | 10.62 |
| PLA2R1 | 18.27 | 23.13 | 39.72 | 12.74 | 18.51 | 39.85 |
| DTX4 | 3.54 | 3.51 | 5.17 | 3.41 | 5.25 | 11.14 |
| HSPG2 | 328.58 | 392.26 | 734.75 | 317.87 | 535.38 | 1128.46 |
| P3H1 | 84.34 | 70.57 | 132.72 | 73.05 | 69.38 | 146.20 |
| VASN | 1.37 | 1.68 | 1.71 | 1.11 | 1.36 | 2.85 |
| EPHB4 | 106.97 | 82.68 | 149.36 | 66.97 | 71.57 | 149.85 |
| CAVIN2 | 94.64 | 105.42 | 112.98 | 62.37 | 61.12 | 127.28 |
| ARRB1 | 35.88 | 43.88 | 84.43 | 25.19 | 36.66 | 75.57 |
| TTLL1 | 3.43 | 2.84 | 7.46 | 3.01 | 3.06 | 6.27 |
| CXCR4 | 1.96 | 1.79 | 5.38 | 1.26 | 2.85 | 5.85 |
| TANC1 | 33.98 | 46.69 | 69.10 | 24.15 | 28.70 | 58.86 |
| TNFRSF9 | 0.04 | 0.15 | 0.13 | 3.91 | 3.91 | 8.02 |
| ANTXR1 | 191.73 | 213.52 | 433.97 | 108.89 | 155.82 | 319.02 |
| TSPAN15 | 20.23 | 22.98 | 43.01 | 7.17 | 11.68 | 23.84 |
| PLPP4 | 2.25 | 2.20 | 4.91 | 1.33 | 1.33 | 2.69 |
| PXDN | 42.95 | 64.61 | 112.67 | 47.58 | 67.13 | 134.67 |
