# Supplementary material for: Downregulation of Engulfment and cell motility 1 (Elmo1) induces quiescence and resistance to poly(I:C)-induced apoptosis in endothelial cells
Source: Cell Death Dis. 2025 Dec 20;17(1):100. doi: 10.1038/s41419-025-08341-1 (PMC12847878; doi:10.1038/s41419-025-08341-1)
Supplement: Supplementary file 12 — Supplementary Table S4 [file 41419_2025_8341_MOESM12_ESM.docx]

| **Table S4. Genes expressed lower in siELMO1 at 24 h** | | | |  |  |  |
| --- | --- | --- | --- | --- | --- | --- |
| Gene | 0H_Mock | 0H_siNT | 0H_siELMO1 | 24H_Mock | 24H_siNT | 24H_siELMO1 |
| SULT1B1 | 104.74 | 20.45 | 41.03 | 90.12 | 190.04 | 29.67 |
| SLC7A11 | 456.39 | 482.42 | 59.84 | 177.69 | 277.61 | 47.06 |
| SERPINB2 | 121.46 | 170.32 | 56.06 | 605.45 | 899.47 | 156.34 |
| RNA5-8SN5 | 1.95 | 5.06 | 1.33 | 8.09 | 10.10 | 1.98 |
| 5_8S_rRNA | 1.95 | 5.06 | 1.33 | 8.09 | 10.10 | 1.98 |
| RNA5-8SN1 | 1.97 | 5.13 | 1.34 | 8.12 | 10.15 | 2.00 |
| RNA5-8SN3 | 2.08 | 5.28 | 1.39 | 8.39 | 10.39 | 2.08 |
| RNA5-8SN2 | 2.08 | 5.28 | 1.39 | 8.39 | 10.39 | 2.08 |
| SYT11 | 18.52 | 17.83 | 4.14 | 14.07 | 18.17 | 3.79 |
| ADIRF-AS1 | 9.92 | 7.80 | 3.74 | 7.26 | 11.25 | 2.66 |
| STEAP2 | 12.71 | 18.96 | 11.29 | 9.65 | 17.51 | 4.56 |
| DHRS2 | 14.51 | 15.18 | 3.42 | 14.69 | 12.85 | 3.72 |
| MATR3 | 192.42 | 156.10 | 164.40 | 140.89 | 172.45 | 57.88 |
| MRPL35 | 57.16 | 64.11 | 13.38 | 48.19 | 59.28 | 19.94 |
| PRKAR2B | 69.41 | 46.73 | 25.49 | 25.53 | 35.37 | 12.01 |
| TMEM158 | 94.76 | 96.22 | 42.90 | 122.93 | 170.92 | 58.32 |
| BMAL2 | 490.02 | 422.94 | 66.97 | 623.38 | 637.09 | 218.69 |
| SNX15 | 31.48 | 27.88 | 7.58 | 32.70 | 34.21 | 11.86 |
| SPRY2 | 26.85 | 37.79 | 27.51 | 53.59 | 72.69 | 25.37 |
| SOCS2 | 8.73 | 12.52 | 8.05 | 8.51 | 10.11 | 3.63 |
| STK38L | 103.12 | 113.22 | 50.05 | 77.21 | 89.97 | 32.60 |
| TMTC3 | 102.49 | 117.06 | 23.60 | 74.94 | 89.84 | 33.04 |
| RAB11FIP2 | 44.80 | 81.01 | 23.44 | 54.11 | 63.19 | 23.25 |
| TXN | 231.68 | 348.24 | 97.19 | 214.71 | 275.30 | 101.45 |
| SHMT2 | 202.22 | 200.21 | 44.92 | 329.64 | 218.64 | 80.79 |
| RN7SK | 9.33 | 13.12 | 6.50 | 22.51 | 24.67 | 9.55 |
| TMEM170A | 24.21 | 45.85 | 12.52 | 49.17 | 49.77 | 20.52 |
| DCLK2 | 7.28 | 8.59 | 3.66 | 10.06 | 11.40 | 4.86 |
| TXNRD1 | 1477.55 | 2018.33 | 667.16 | 1295.44 | 1407.12 | 609.30 |
| DKK1 | 149.51 | 139.96 | 123.02 | 179.99 | 183.53 | 81.13 |
| SH2D5 | 6.95 | 11.48 | 3.45 | 12.29 | 12.36 | 5.49 |
| PLEKHB2 | 193.32 | 124.33 | 56.98 | 208.43 | 229.20 | 103.16 |
| MORF4L2 | 788.40 | 945.67 | 308.06 | 741.09 | 785.63 | 359.12 |
| NQO1 | 606.14 | 703.72 | 225.93 | 374.62 | 346.58 | 159.70 |
| STEAP1 | 35.46 | 35.32 | 26.52 | 48.43 | 48.20 | 23.16 |
| NEXN | 5.62 | 1.65 | 6.04 | 16.45 | 12.43 | 6.02 |
| SEC61A1 | 340.86 | 336.23 | 148.09 | 454.34 | 416.75 | 202.31 |
| LURAP1L | 6.60 | 9.97 | 8.58 | 13.66 | 11.45 | 5.61 |
