# Supplementary material for: Downregulation of Engulfment and cell motility 1 (Elmo1) induces quiescence and resistance to poly(I:C)-induced apoptosis in endothelial cells
Source: Cell Death Dis. 2025 Dec 20;17(1):100. doi: 10.1038/s41419-025-08341-1 (PMC12847878; doi:10.1038/s41419-025-08341-1)
Supplement: Supplementary file 13 — Supplementary Table S5 [file 41419_2025_8341_MOESM13_ESM.docx]

| **Table S5. Genes upregulated at 24 h in Mock** | | | |  |  |  |
| --- | --- | --- | --- | --- | --- | --- |
| Gene | 0H_Mock | 0H_siNT | 0H_siELMO1 | 24H_Mock | 24H_siNT | 24H_siELMO1 |
| CXCL11 | 0.01 | 0.00 | 0.00 | 74.46 | 14.80 | 51.02 |
| ACOD1 | 0.01 | 0.02 | 0.00 | 57.24 | 3.28 | 10.51 |
| GBP4 | 0.04 | 0.09 | 0.17 | 230.81 | 92.75 | 185.76 |
| BST2 | 0.14 | 0.29 | 0.45 | 173.69 | 79.66 | 171.10 |
| GRIP2 | 0.03 | 0.08 | 0.05 | 29.55 | 17.96 | 35.30 |
| RSAD2 | 0.32 | 0.17 | 0.51 | 358.48 | 110.04 | 513.45 |
| GBP5 | 0.10 | 0.10 | 0.00 | 97.50 | 20.33 | 46.07 |
| CCL5 | 0.23 | 0.17 | 0.21 | 176.28 | 33.47 | 83.22 |
| IL6 | 1.40 | 1.42 | 2.63 | 427.98 | 44.85 | 143.05 |
| BISPR | 0.14 | 0.25 | 0.28 | 37.60 | 14.36 | 32.05 |
| CXCL1 | 0.62 | 0.79 | 0.30 | 126.82 | 7.39 | 120.20 |
| CCL20 | 0.25 | 0.87 | 0.24 | 44.87 | 18.23 | 49.81 |
| OASL | 7.19 | 5.62 | 3.41 | 1196.01 | 286.45 | 936.51 |
| C3 | 0.33 | 0.72 | 0.40 | 48.03 | 32.17 | 78.15 |
| IL4I1 | 0.82 | 0.68 | 1.72 | 79.03 | 33.92 | 69.29 |
| CXCL2 | 1.39 | 2.36 | 0.92 | 128.48 | 24.36 | 67.81 |
| C1S | 0.40 | 0.35 | 0.37 | 34.76 | 10.34 | 22.09 |
| CXCL3 | 0.54 | 0.89 | 0.75 | 44.18 | 8.42 | 22.00 |
| HERC5 | 1.76 | 1.36 | 1.24 | 135.79 | 61.99 | 129.13 |
| CXCL8 | 15.65 | 20.67 | 17.78 | 1152.43 | 237.31 | 589.79 |
| NUAK2 | 0.75 | 1.88 | 1.27 | 36.72 | 17.60 | 43.08 |
| SOD2 | 79.33 | 74.27 | 61.80 | 3787.60 | 1143.87 | 2775.31 |
| LGALS9 | 0.91 | 0.72 | 1.44 | 39.06 | 26.99 | 71.32 |
| CSF3 | 4.11 | 2.74 | 2.52 | 154.16 | 14.49 | 107.01 |
| ENSG00000283265 | 0.81 | 0.81 | 0.80 | 23.46 | 9.69 | 19.84 |
| KLF4 | 5.38 | 10.65 | 8.91 | 154.73 | 37.01 | 101.18 |
| RIGI | 45.53 | 50.17 | 79.37 | 1240.56 | 640.28 | 1295.31 |
| APOL1 | 10.03 | 10.97 | 14.03 | 205.20 | 116.44 | 244.98 |
| NFKBIZ | 13.71 | 12.21 | 15.67 | 271.22 | 41.55 | 186.67 |
| CEACAM1 | 5.97 | 9.56 | 24.93 | 115.54 | 89.16 | 191.72 |
| CCL2 | 16.15 | 23.38 | 27.82 | 292.62 | 91.51 | 436.11 |
| CEBPD | 3.03 | 2.54 | 4.61 | 47.65 | 8.51 | 47.99 |
| ENSG00000291143 | 1.72 | 3.05 | 2.25 | 26.89 | 13.88 | 31.55 |
| TNFAIP2 | 20.99 | 31.96 | 26.01 | 311.89 | 102.21 | 372.05 |
| IL7R | 44.59 | 37.12 | 53.19 | 637.55 | 240.25 | 531.81 |
| PTGS2 | 17.96 | 5.98 | 73.35 | 233.80 | 35.64 | 121.32 |
| ZC3H12A | 3.10 | 4.32 | 3.27 | 39.52 | 8.48 | 52.97 |
| FST | 11.59 | 7.83 | 13.14 | 127.42 | 31.19 | 81.94 |
| CLDN1 | 11.11 | 13.69 | 7.59 | 92.65 | 68.11 | 153.33 |
| TFPI2 | 4.81 | 2.75 | 13.13 | 38.69 | 19.05 | 67.98 |
| SLC2A13 | 7.35 | 7.33 | 9.17 | 42.92 | 12.20 | 38.10 |
| NKX3-1 | 10.41 | 8.80 | 8.30 | 57.36 | 20.46 | 52.56 |
| GRB10 | 118.64 | 51.43 | 147.15 | 620.00 | 128.62 | 439.38 |
| PLCG2 | 11.62 | 11.06 | 12.23 | 54.85 | 21.18 | 60.69 |
| P2RX4 | 5.79 | 5.70 | 7.77 | 26.61 | 10.12 | 20.92 |
| TIFA | 11.00 | 11.02 | 9.84 | 45.89 | 22.93 | 49.71 |
| SERPINE1 | 3298.19 | 1188.63 | 5735.13 | 13284.93 | 6535.02 | 15829.94 |
| CD34 | 20.55 | 18.26 | 34.85 | 79.23 | 42.16 | 130.76 |
| ABCA1 | 22.40 | 16.05 | 50.55 | 81.99 | 64.20 | 140.01 |
| BBC3 | 23.44 | 15.99 | 27.65 | 78.99 | 35.40 | 75.67 |
| MYLK | 41.17 | 25.29 | 52.12 | 138.08 | 91.07 | 188.51 |
| ZFP36L2 | 84.46 | 120.36 | 108.86 | 281.56 | 122.64 | 308.18 |
| SERPINE2 | 19.25 | 13.98 | 27.73 | 62.44 | 25.15 | 65.17 |
| RELN | 31.80 | 18.03 | 34.23 | 102.77 | 43.00 | 131.70 |
| OGFR | 120.46 | 26.50 | 105.38 | 358.09 | 99.23 | 327.65 |
| PXK | 19.37 | 21.73 | 24.52 | 57.47 | 33.16 | 79.93 |
| CCN1 | 310.62 | 182.29 | 408.13 | 906.84 | 325.06 | 658.13 |
| C5orf15 | 83.50 | 104.57 | 149.73 | 233.87 | 155.75 | 322.57 |
| FRMD6-AS2 | 11.42 | 9.78 | 13.98 | 30.88 | 8.24 | 47.33 |
| CASP7 | 36.86 | 12.55 | 46.76 | 99.43 | 36.54 | 104.31 |
| MBD5 | 7.92 | 6.02 | 12.89 | 20.71 | 11.55 | 23.79 |
| S1PR1 | 84.91 | 47.23 | 113.44 | 220.47 | 115.36 | 250.65 |
| COL5A2 | 269.72 | 153.12 | 691.61 | 674.72 | 315.33 | 1115.69 |
| PDP1 | 98.85 | 56.48 | 131.25 | 232.55 | 84.25 | 255.26 |
| PLCB4 | 245.63 | 71.79 | 180.22 | 563.40 | 132.80 | 476.49 |
| PLAGL1 | 24.68 | 21.27 | 41.98 | 55.52 | 37.14 | 75.67 |
| MAP3K7CL | 14.90 | 10.47 | 20.58 | 32.34 | 11.37 | 53.99 |
| HYOU1 | 177.34 | 155.64 | 382.67 | 383.91 | 202.93 | 411.03 |
| HAVCR1 | 28.36 | 36.61 | 58.10 | 61.04 | 38.62 | 77.88 |
| SDC4 | 135.34 | 73.90 | 85.97 | 290.77 | 132.58 | 348.88 |
| COL17A1 | 61.23 | 24.15 | 29.32 | 130.21 | 45.76 | 105.48 |
| AMTN | 402.27 | 173.06 | 634.77 | 813.67 | 296.42 | 783.35 |
