# Supplementary material for: Downregulation of Engulfment and cell motility 1 (Elmo1) induces quiescence and resistance to poly(I:C)-induced apoptosis in endothelial cells
Source: Cell Death Dis. 2025 Dec 20;17(1):100. doi: 10.1038/s41419-025-08341-1 (PMC12847878; doi:10.1038/s41419-025-08341-1)

Original western blot: Fig. 2b

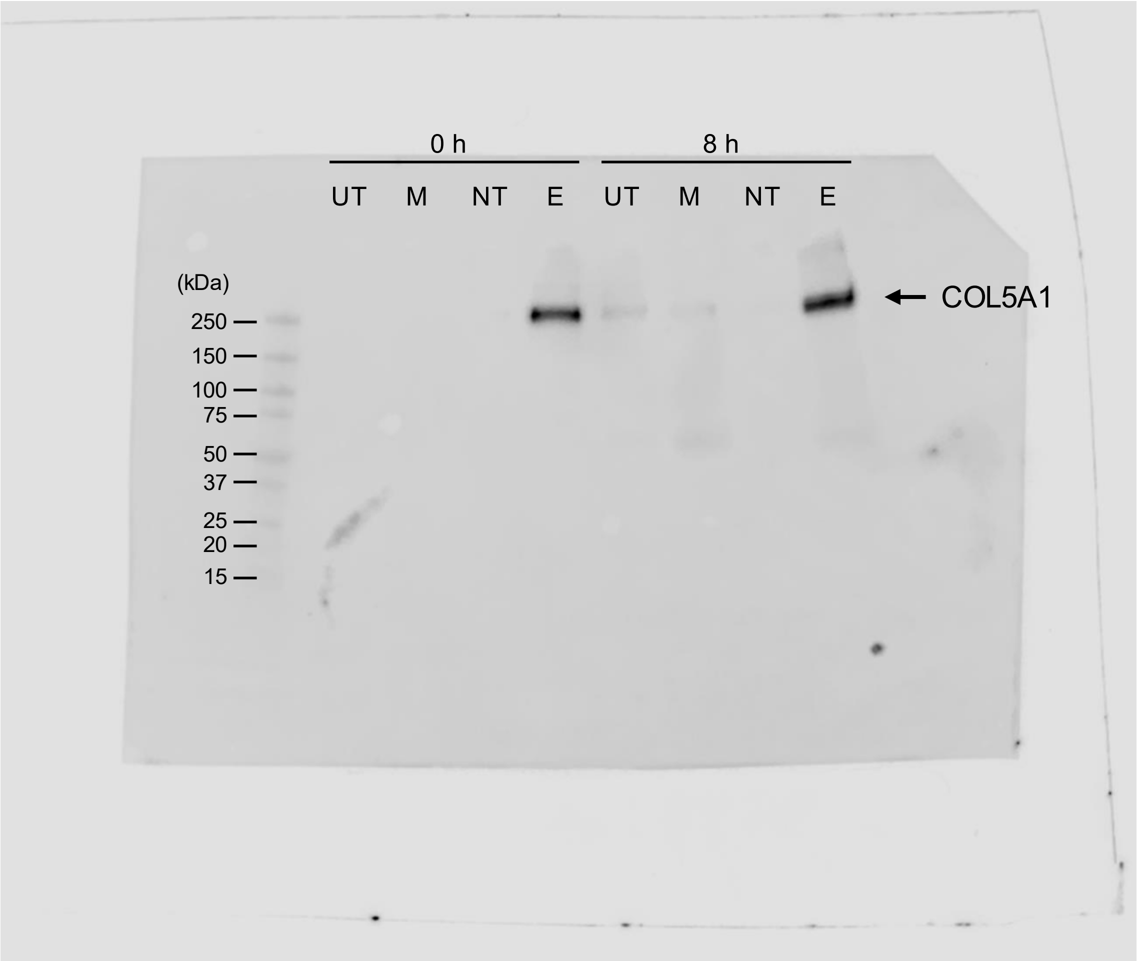

Protein standards: #1610374 (Bio-Rad)

Original western blot: Fig. 2b and 5d

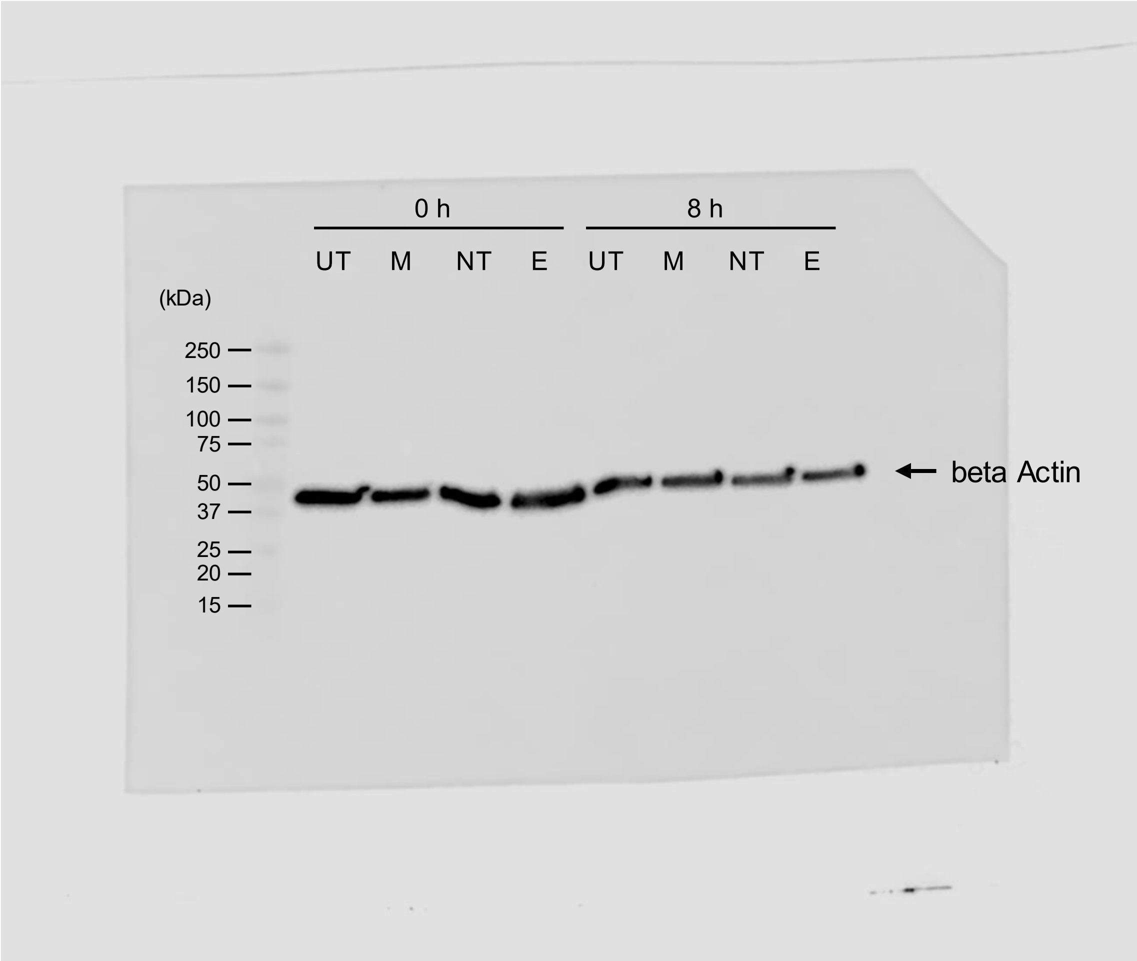

Protein standards: #1610374 (Bio-Rad)

Original western blot: Fig. 5d

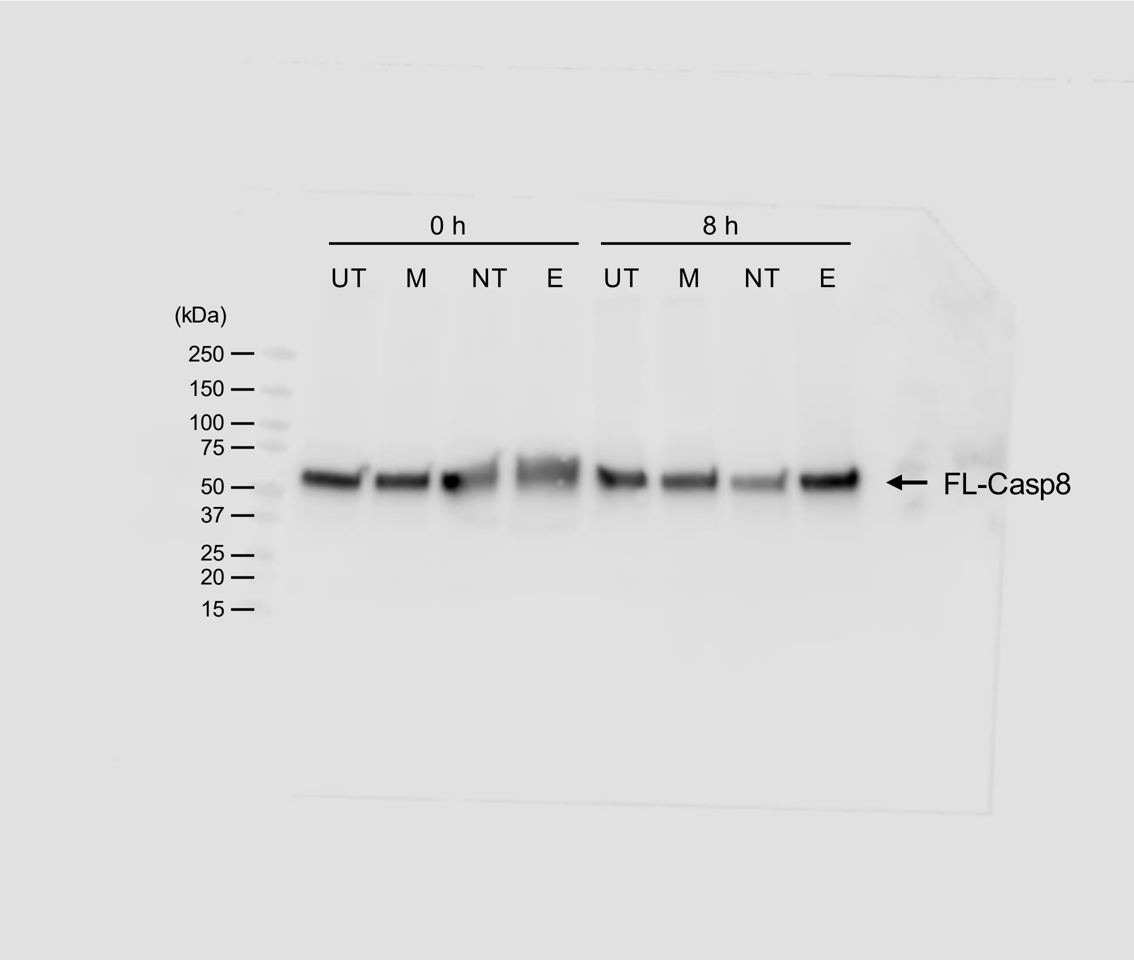

Protein standards: #1610374 (Bio-Rad)

Original western blot: Fig. 5d

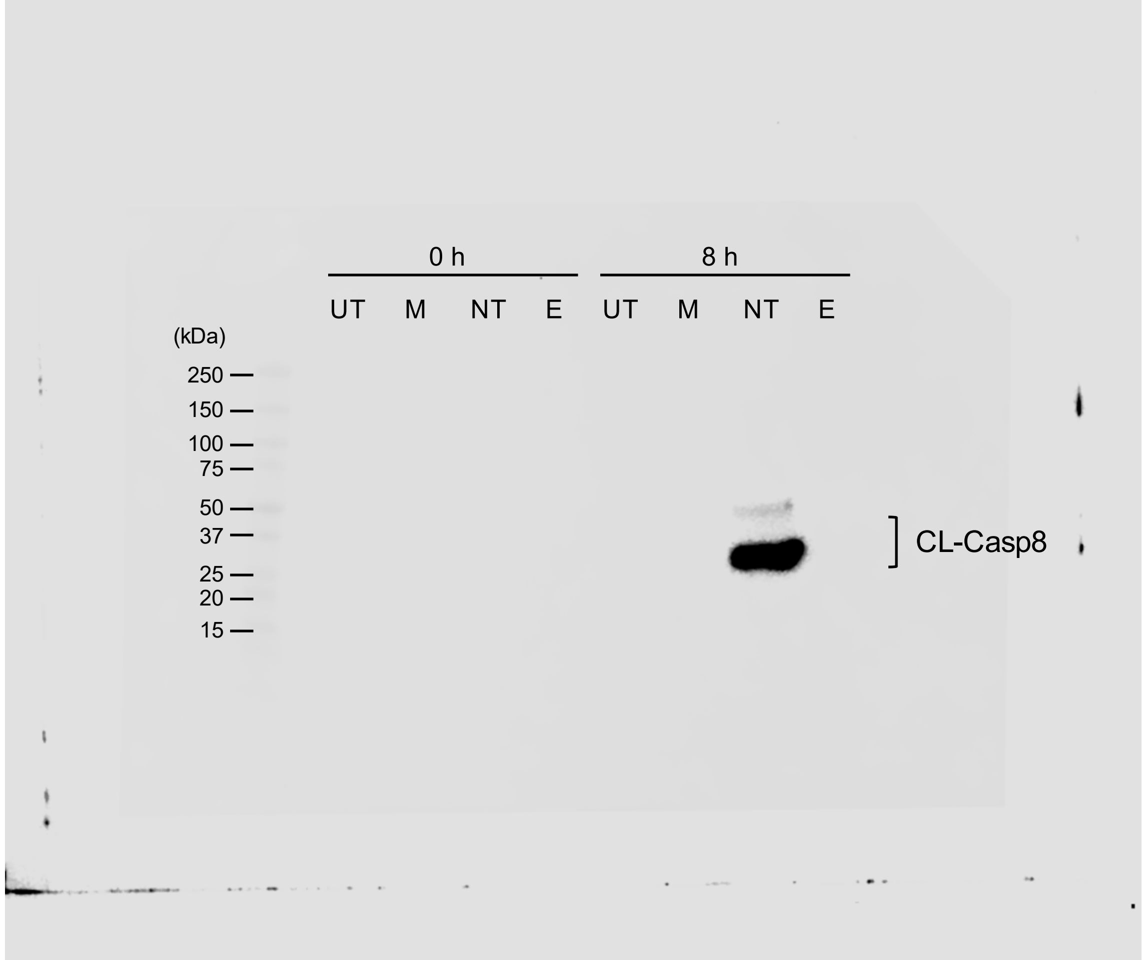

Protein standards: #1610374 (Bio-Rad)

Original western blot: Fig 5d

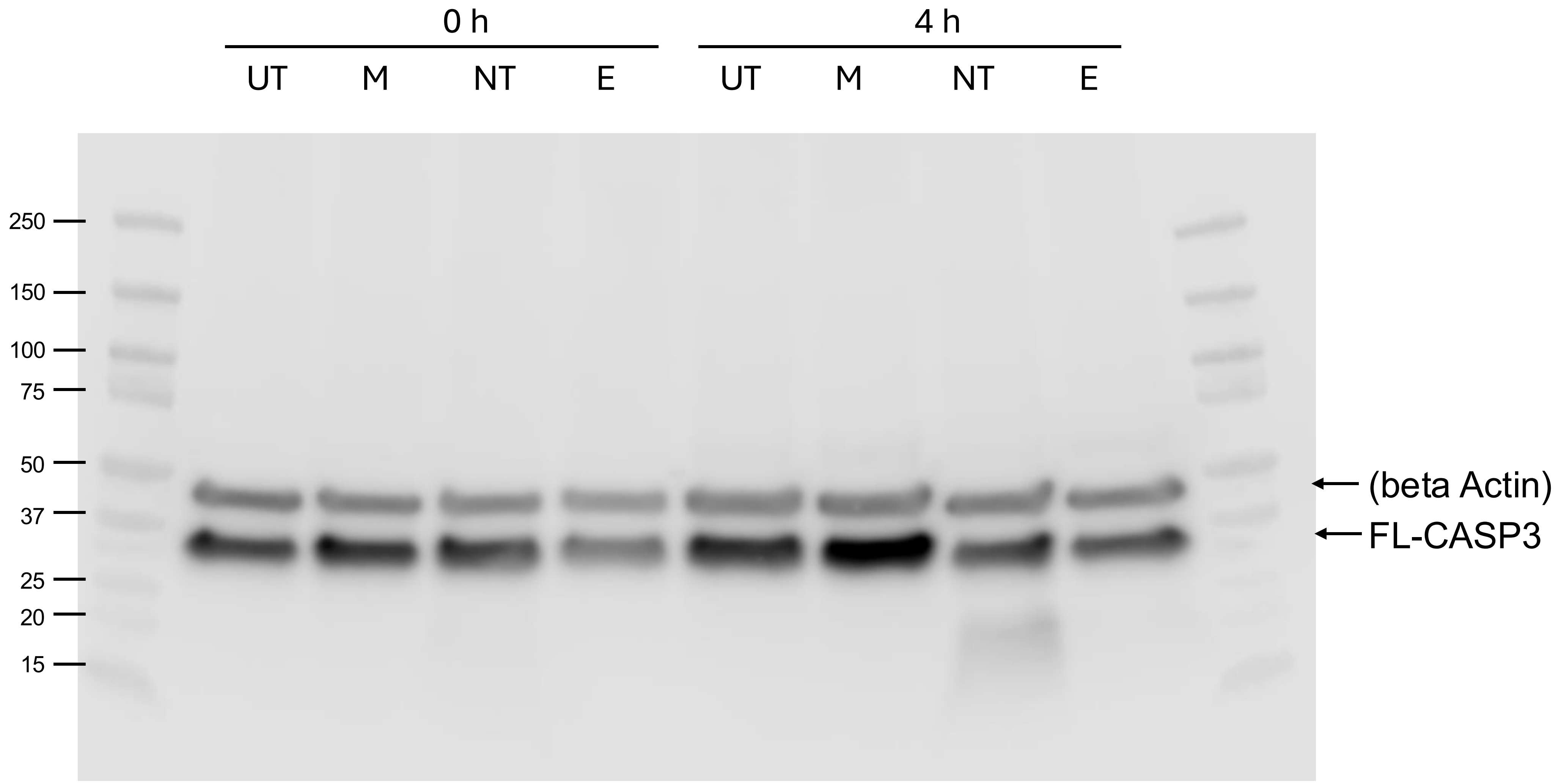

Protein standards: #1610374 (Bio-Rad)

Original western blot: Fig 5d

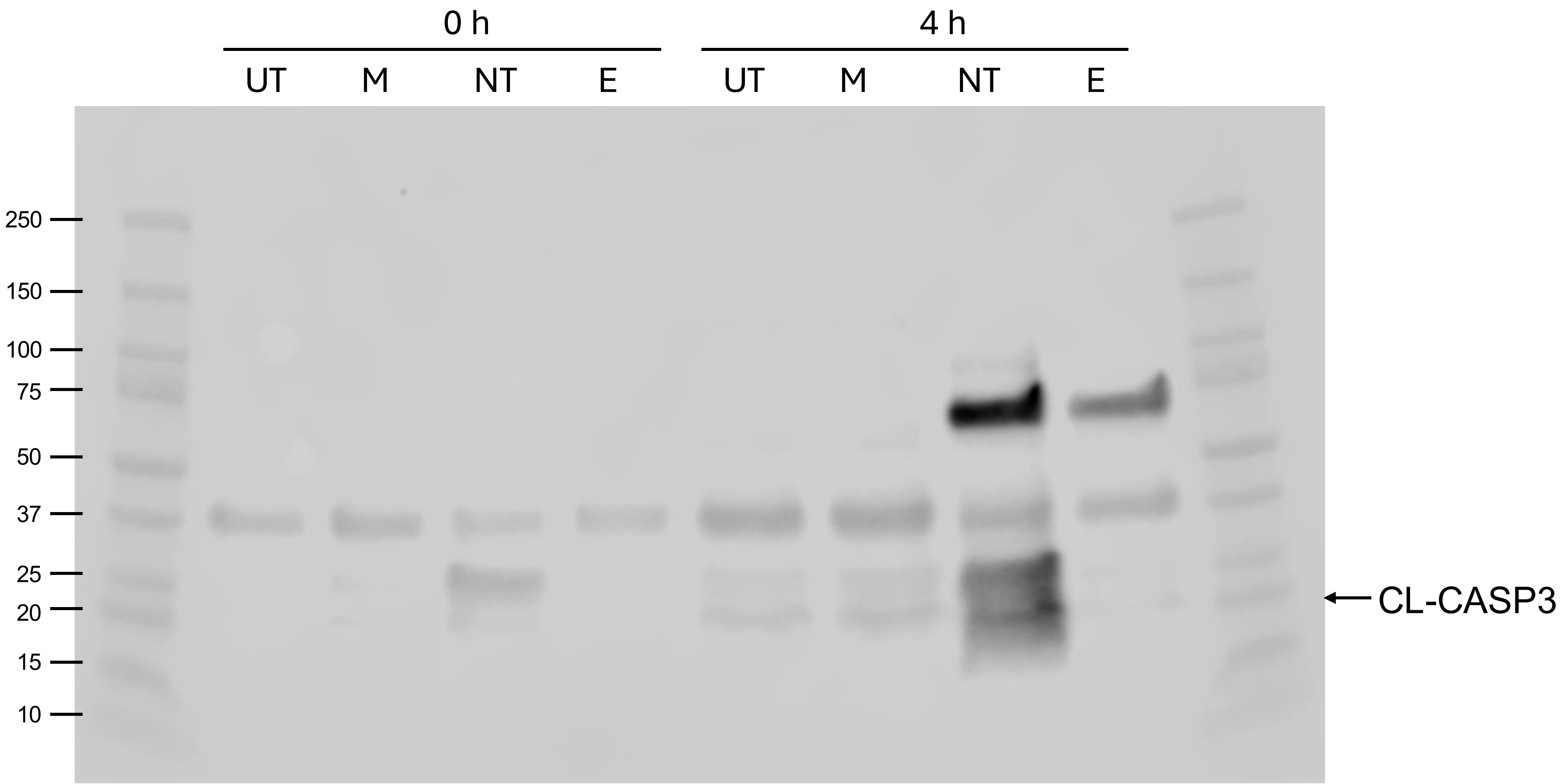

Protein standards: #1610374 (Bio-Rad)

Original western blot: Fig 5d

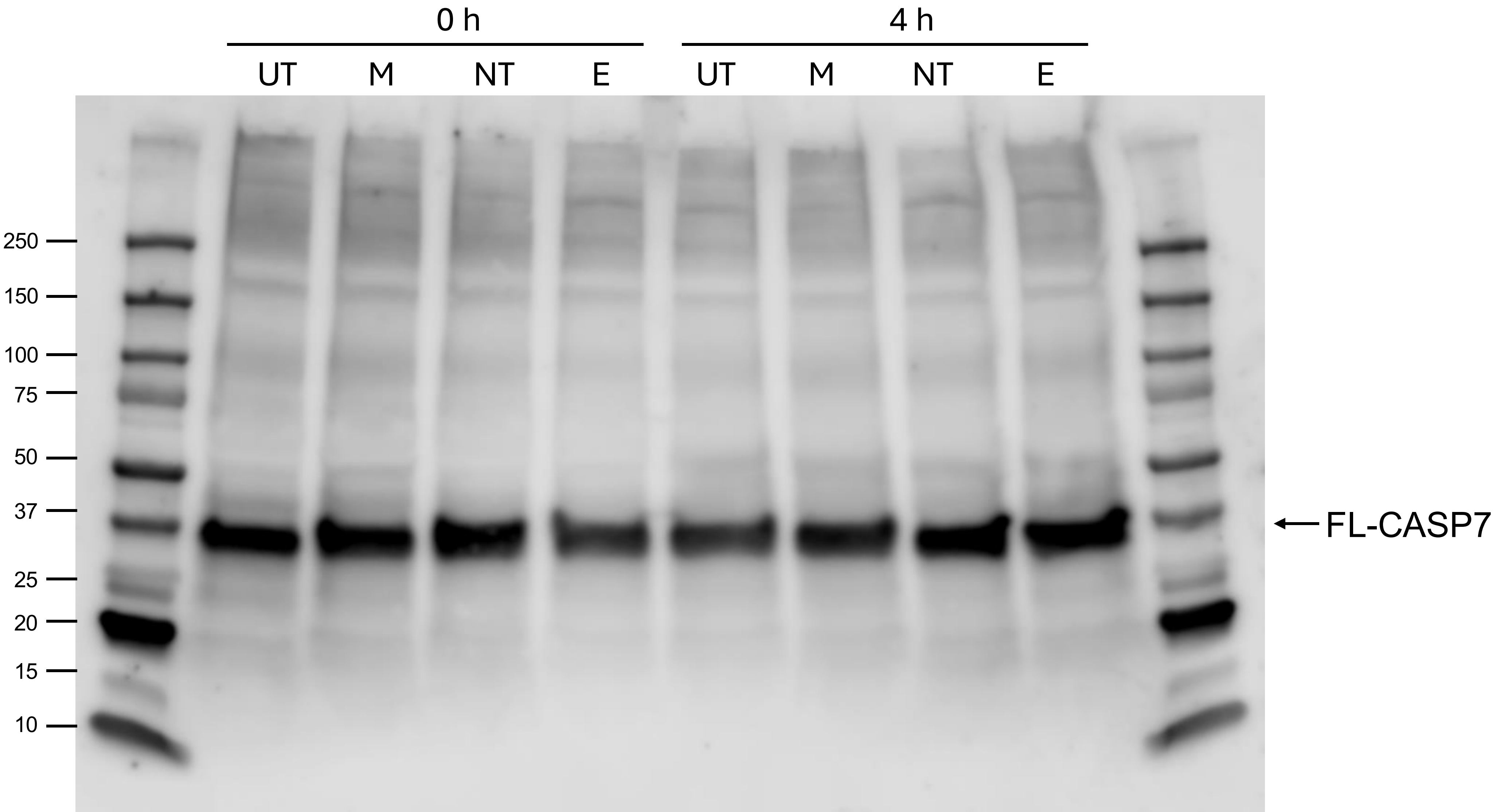

Protein standards: #1610374 (Bio-Rad)

Original western blot: Fig 5d

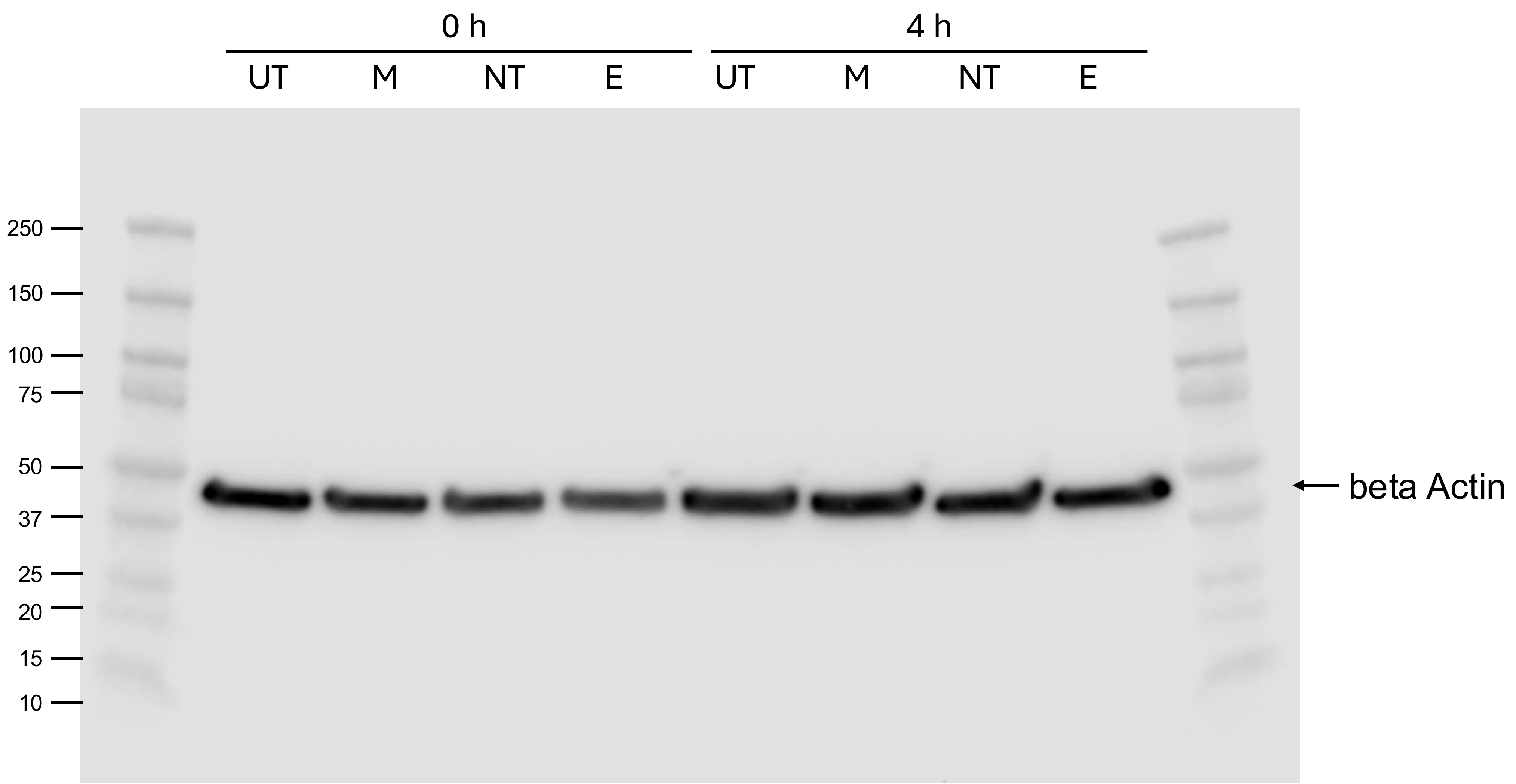

Protein standards: #1610374 (Bio-Rad)

Original western blot: Fig S1b

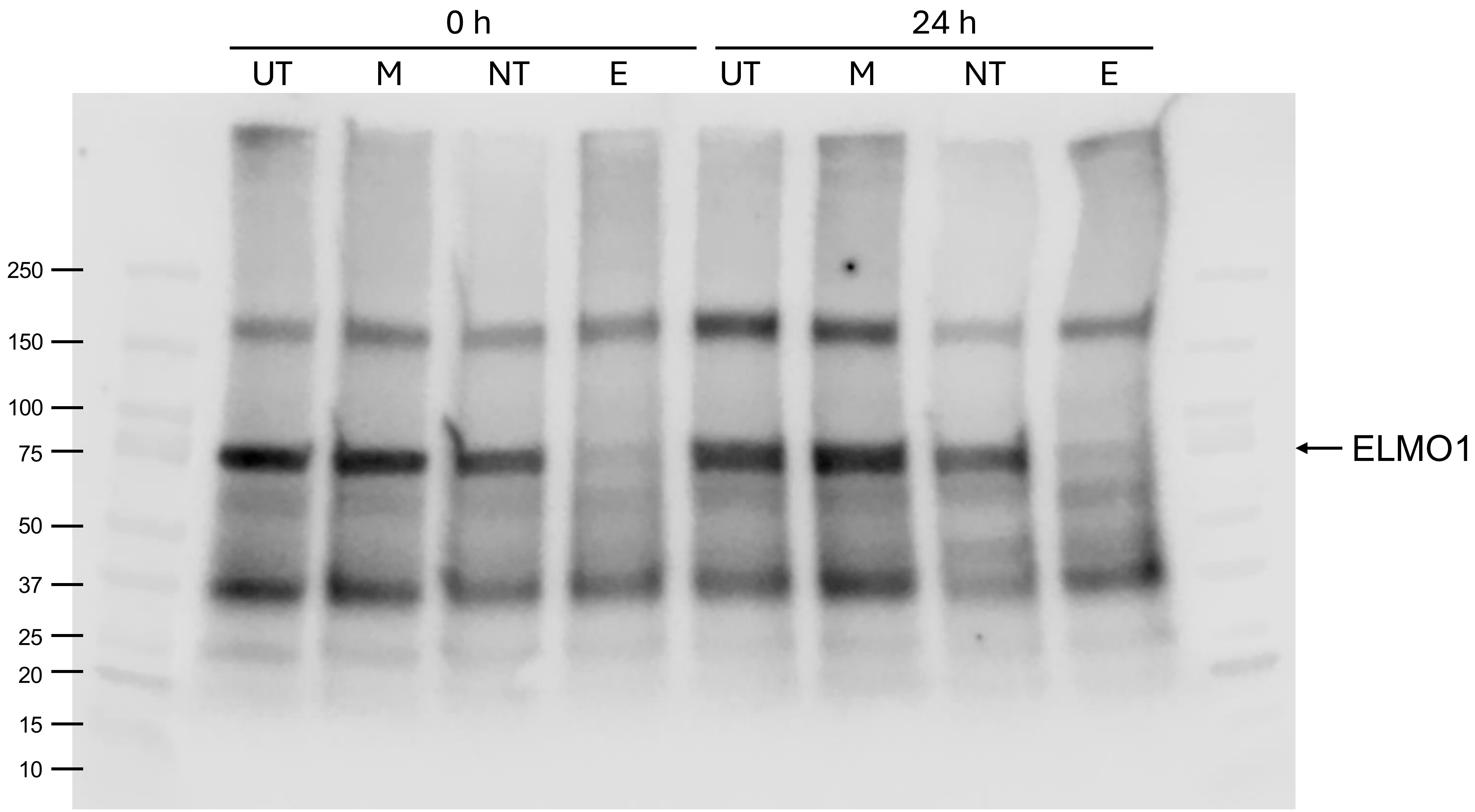

Protein standards: #1610374 (Bio-Rad)

Original western blot: Fig S1b

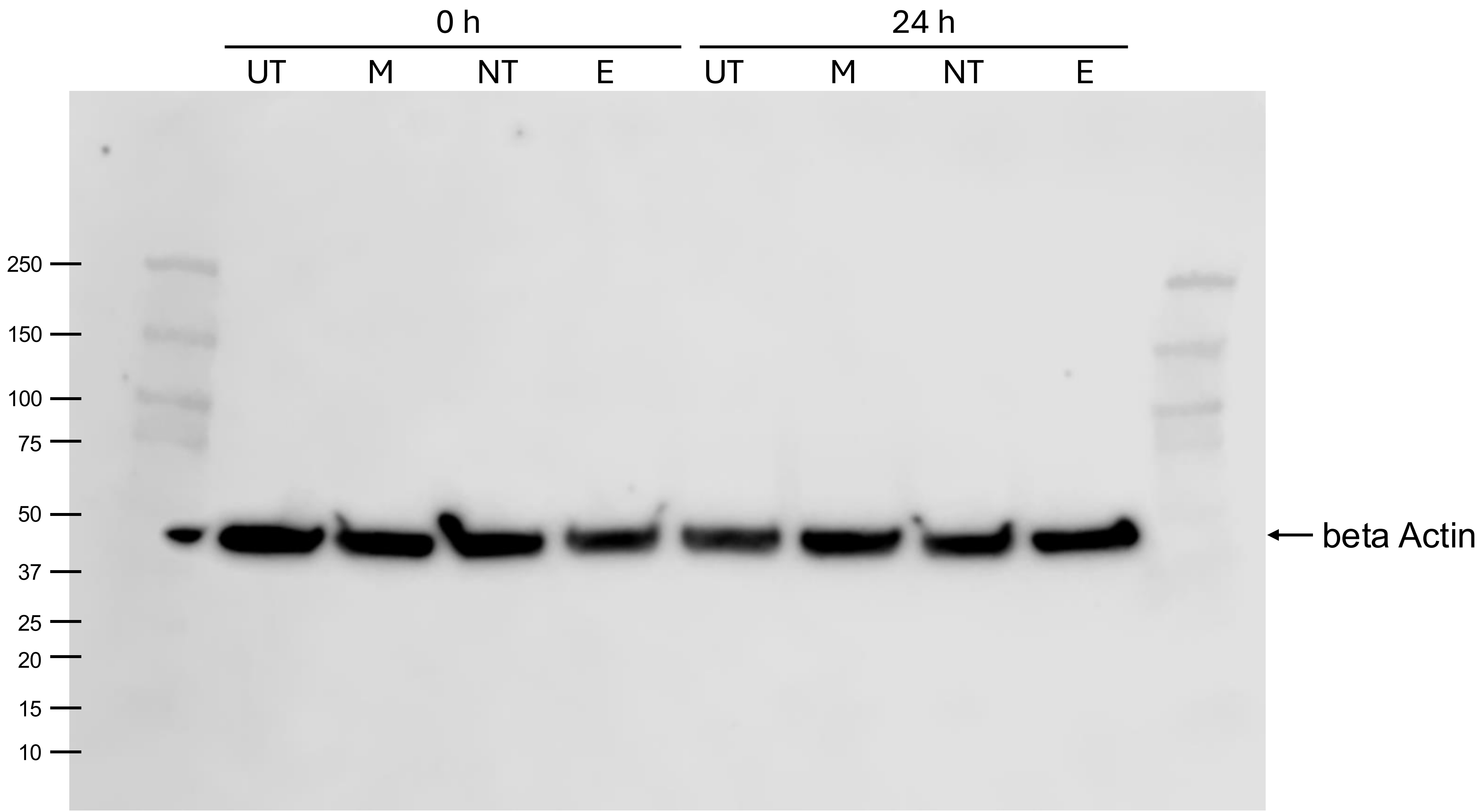

Protein standards: #1610374 (Bio-Rad)

Original western blot: Fig S5b

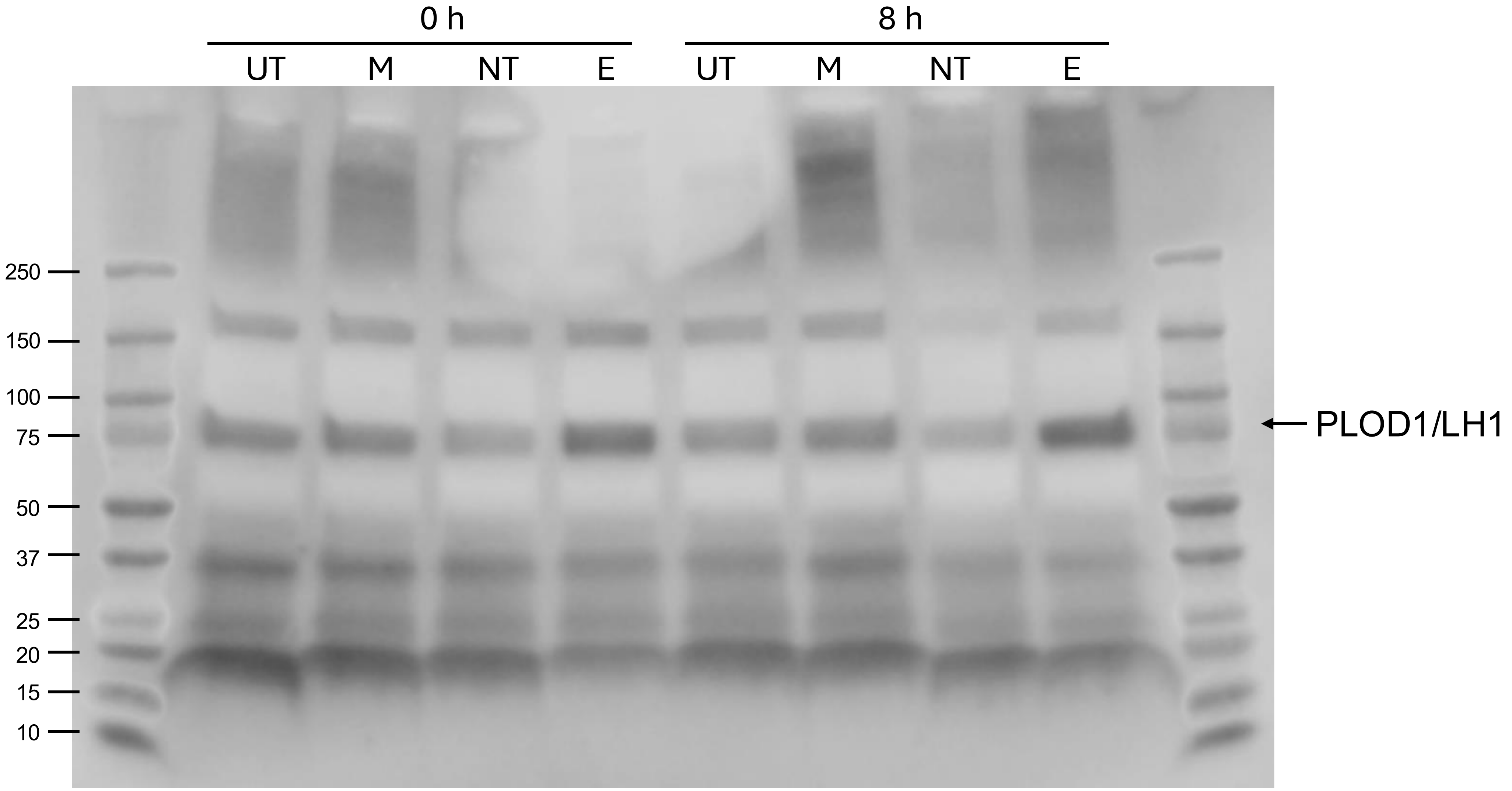

Protein standards: #1610374 (Bio-Rad)

Original western blot: Fig S5b

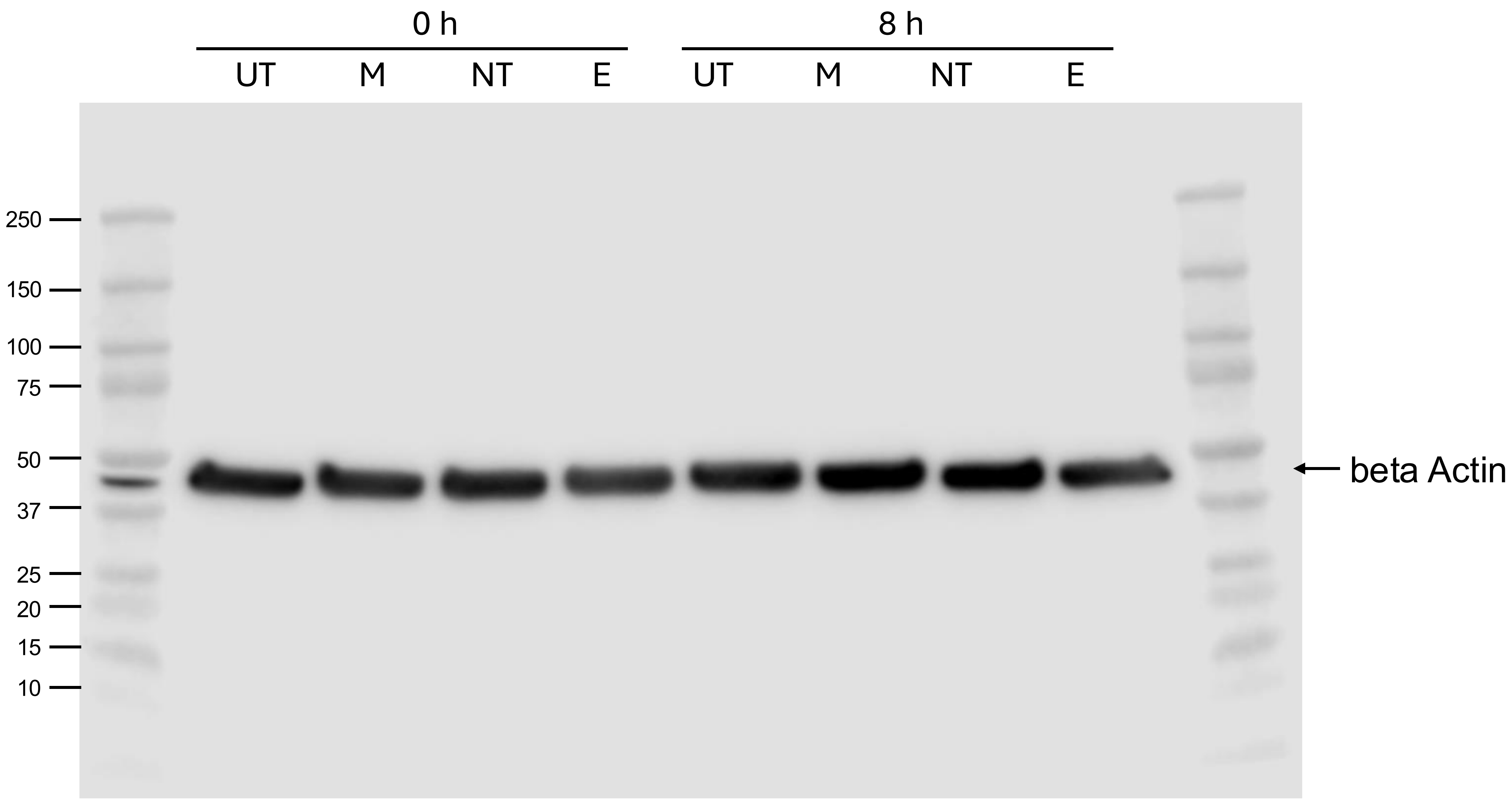

Protein standards: #1610374 (Bio-Rad)

Original western blot: Fig S8a

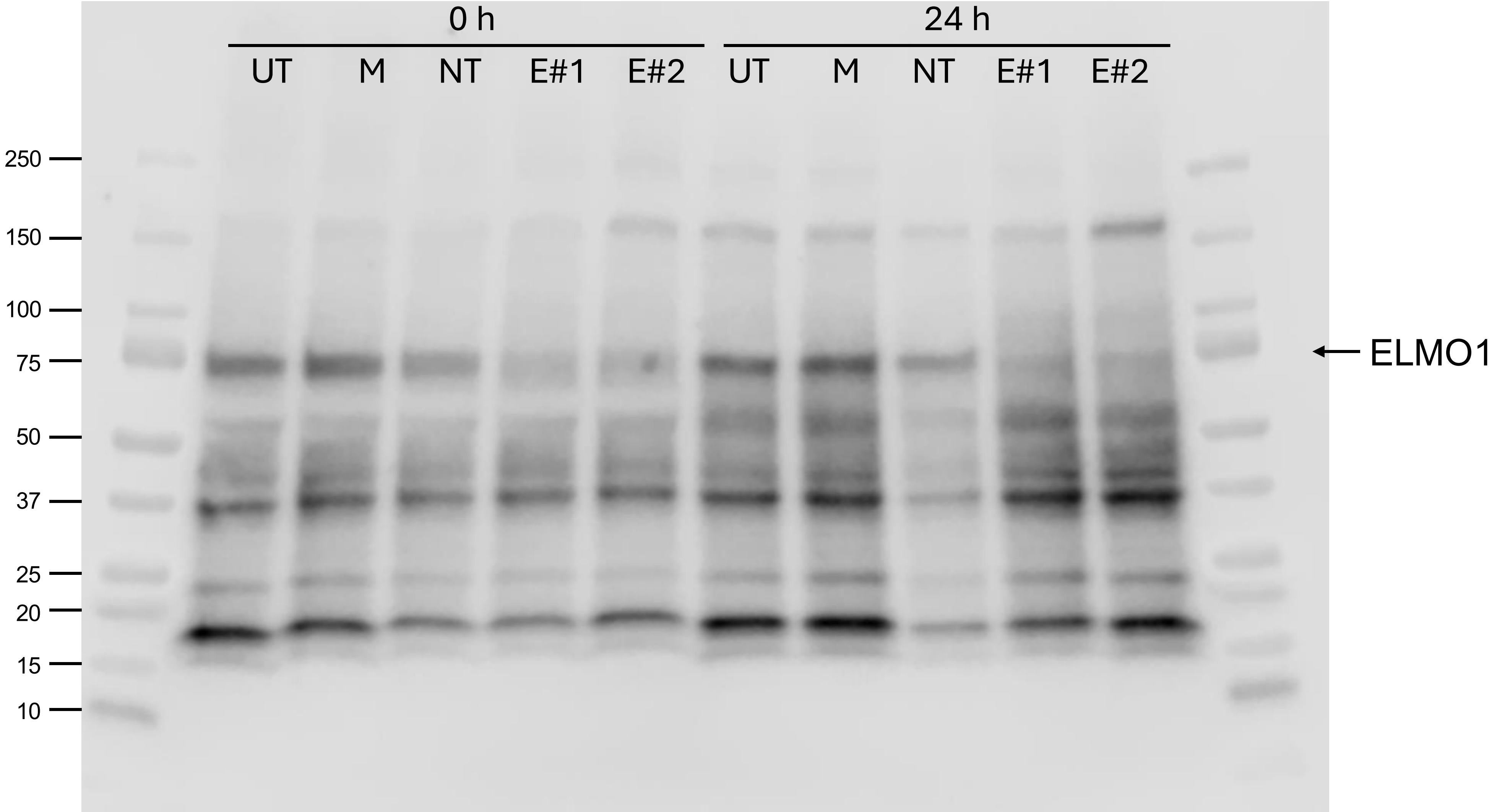

Protein standards: #1610374 (Bio-Rad)

Original western blot: Fig S8a

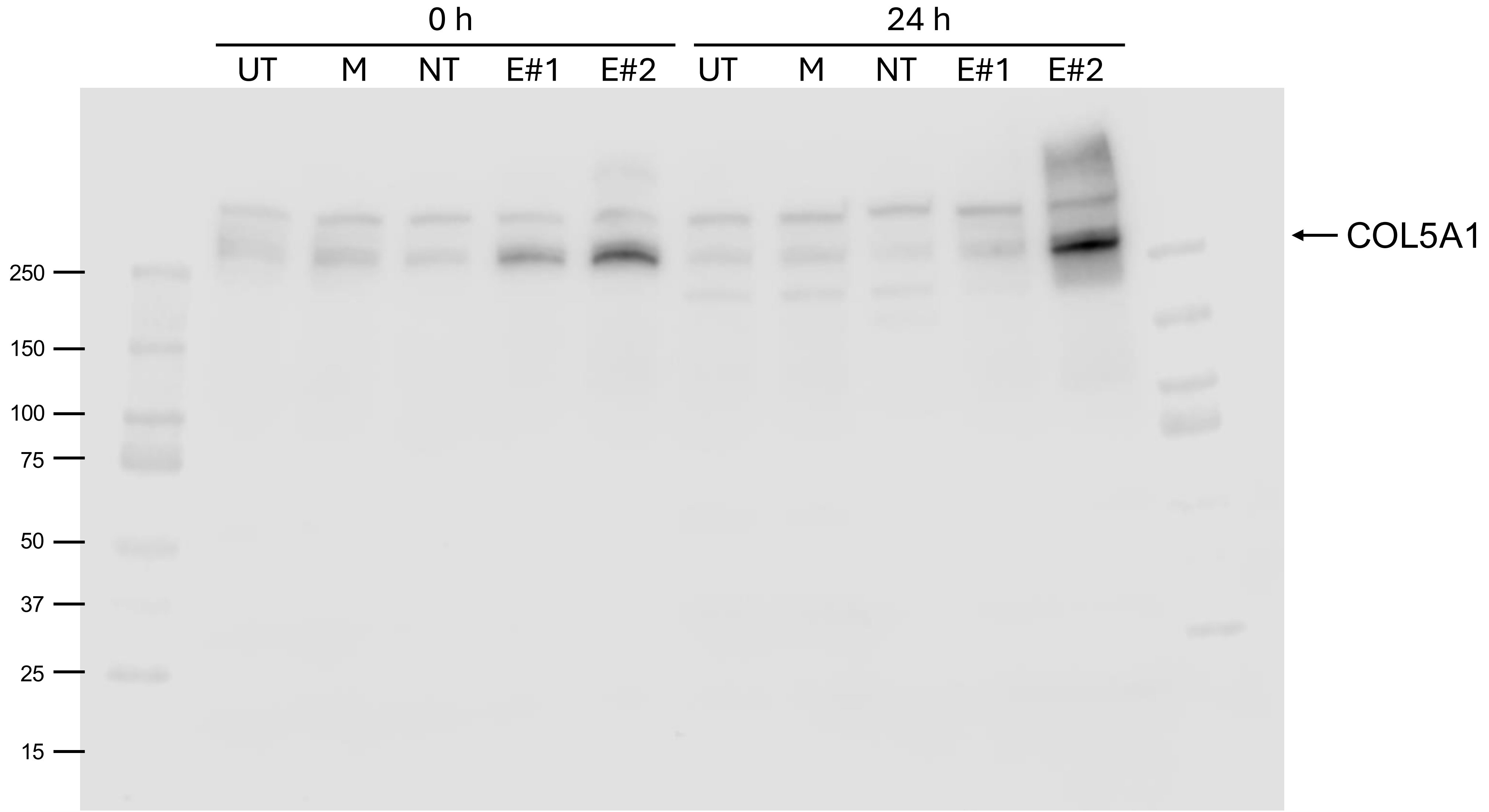

Protein standards: #1610374 (Bio-Rad)

Original western blot: Fig S8a

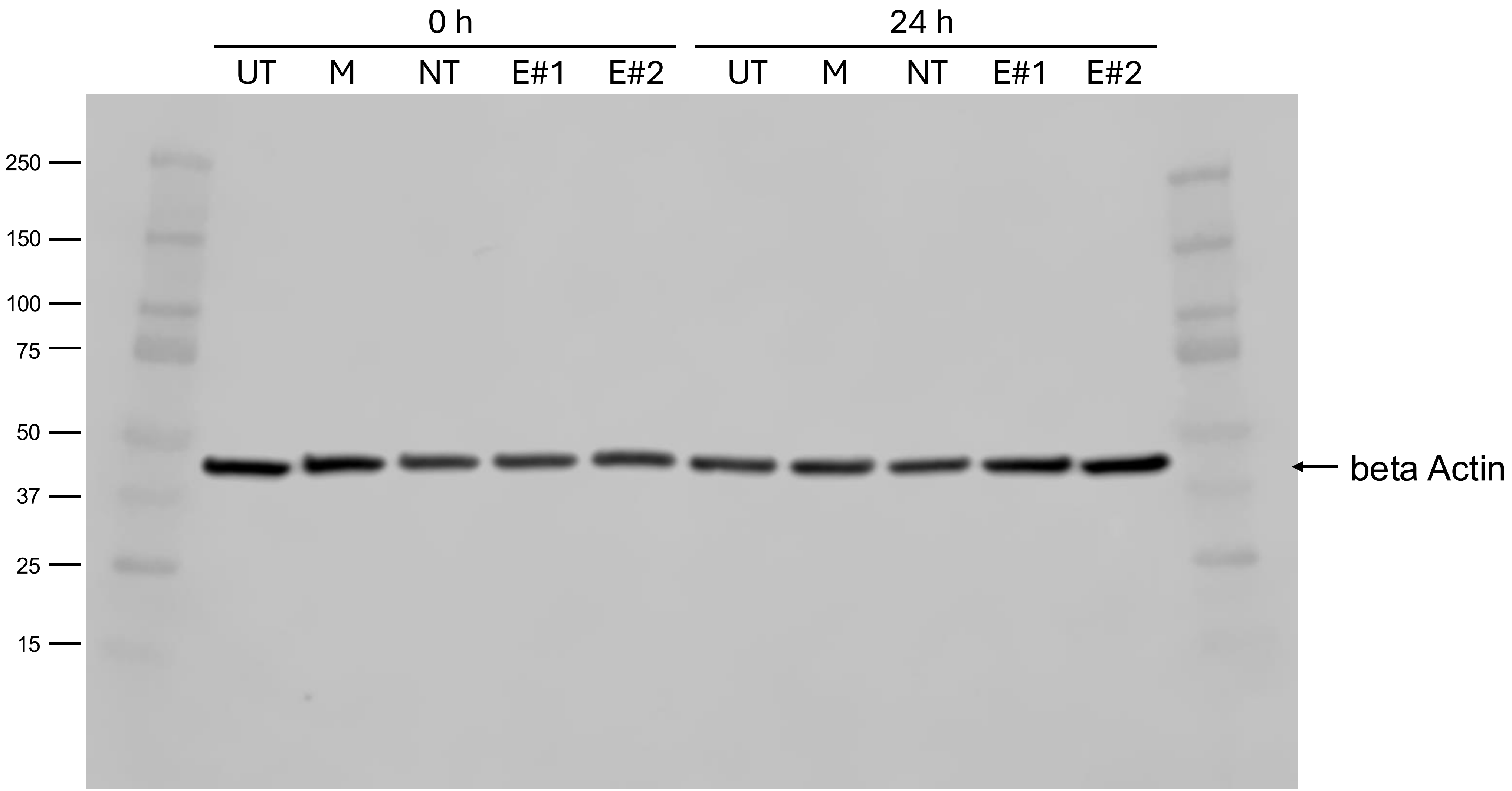

Protein standards: #1610374 (Bio-Rad)

Original western blot: Fig S8c

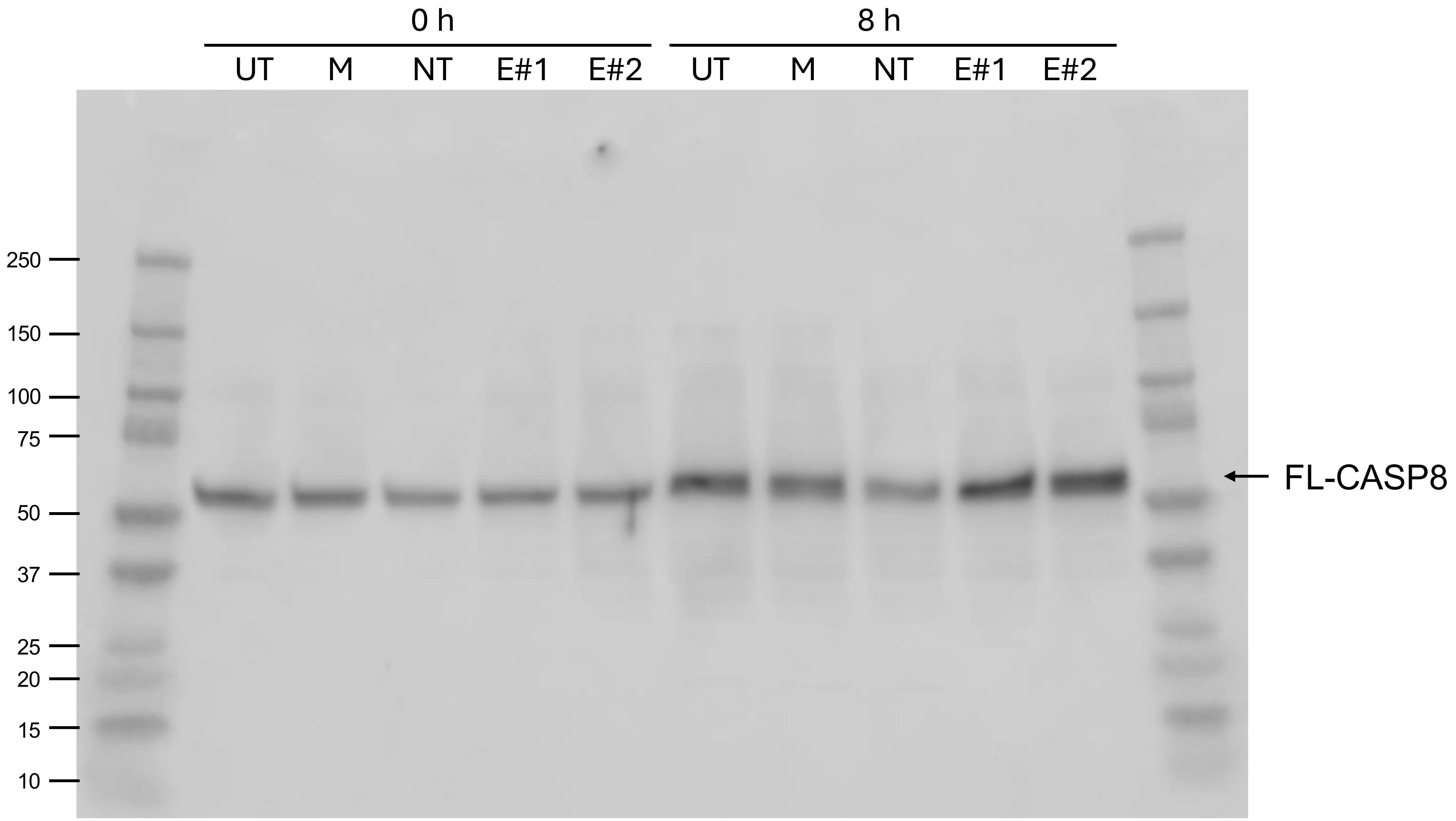

Protein standards: #1610374 (Bio-Rad)

Original western blot: Fig S8c

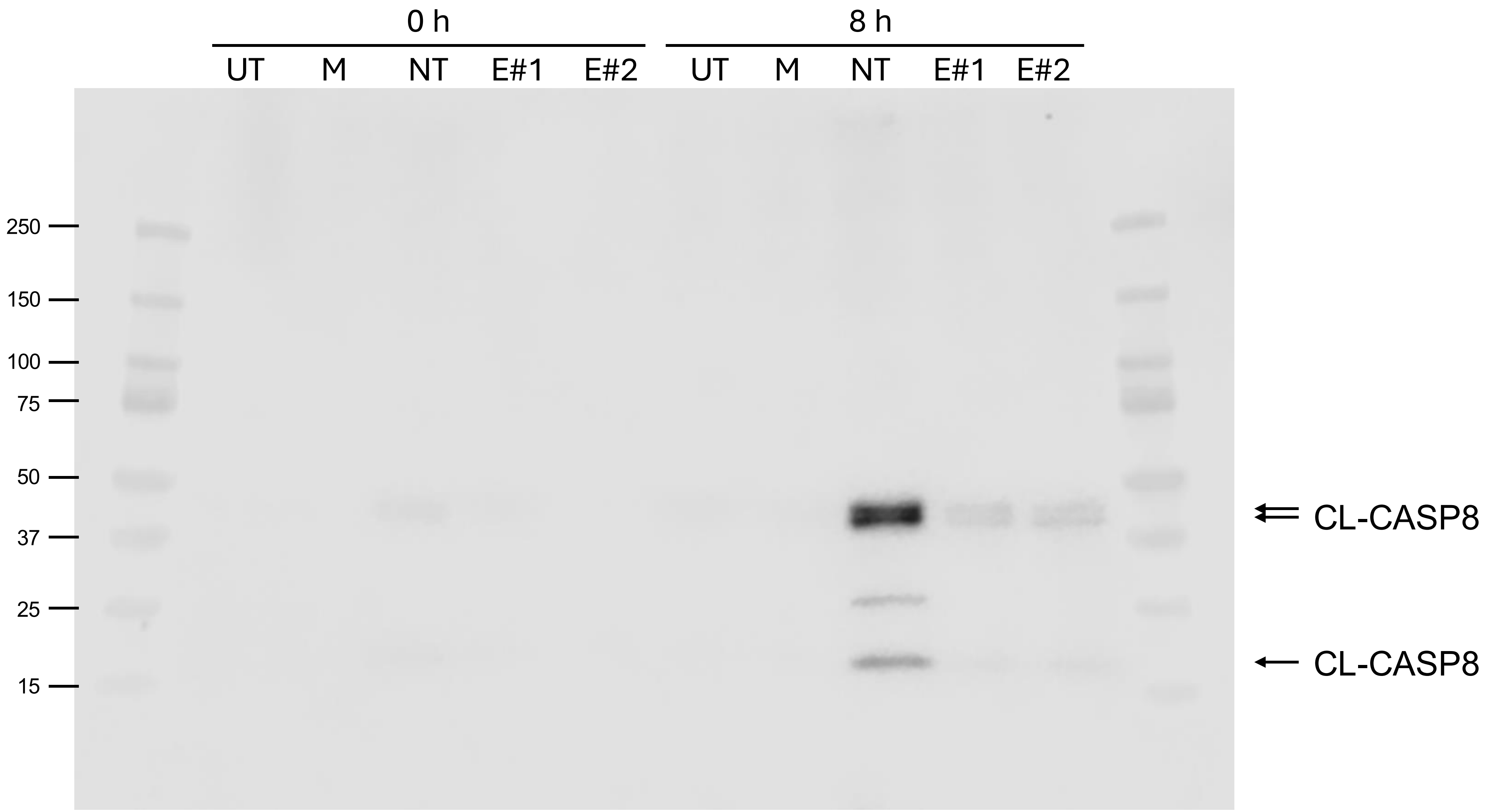

Protein standards: #1610374 (Bio-Rad)

Original western blot: Fig S8c

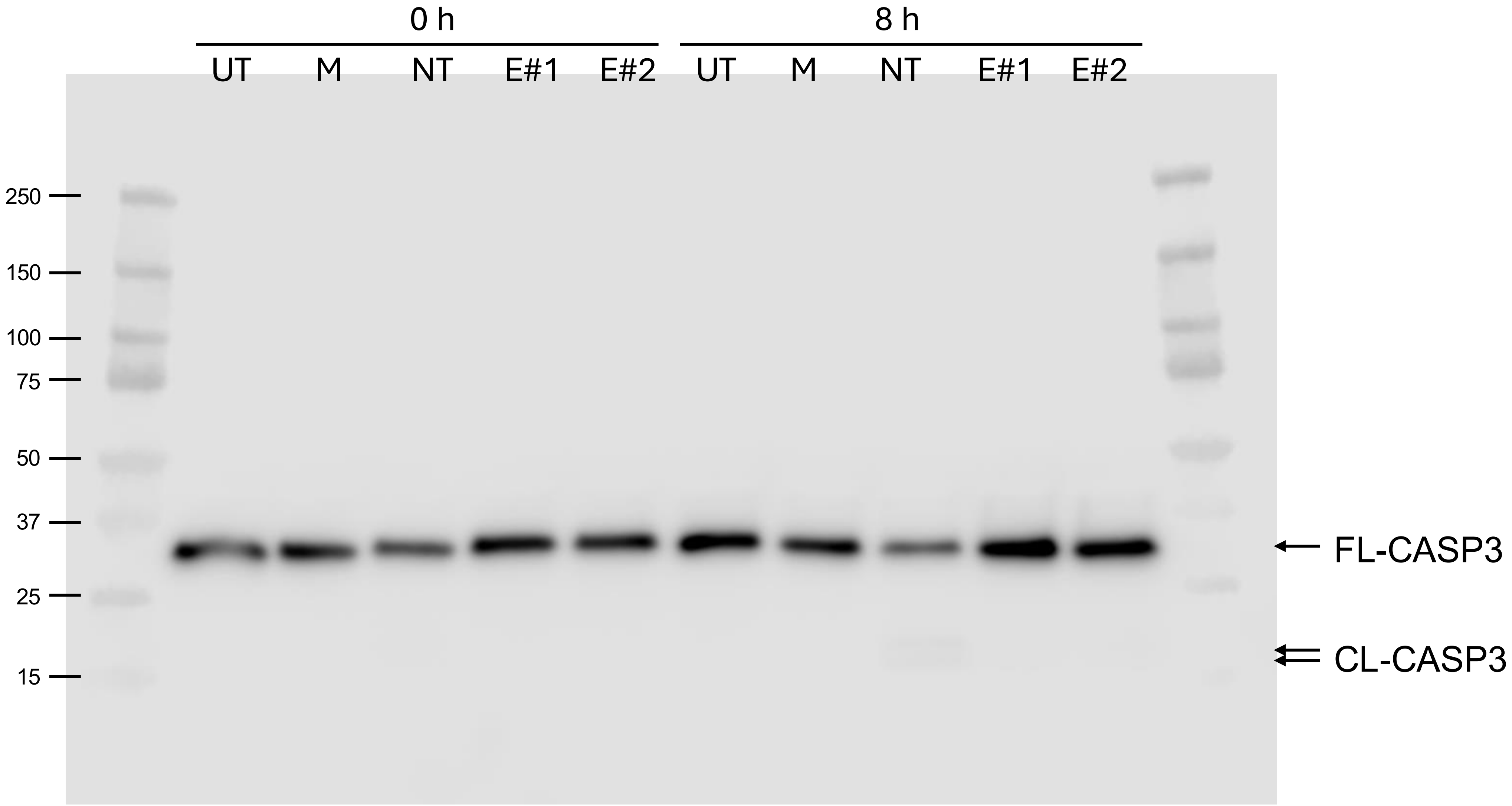

Protein standards: #1610374 (Bio-Rad)

Original western blot: Fig S8c

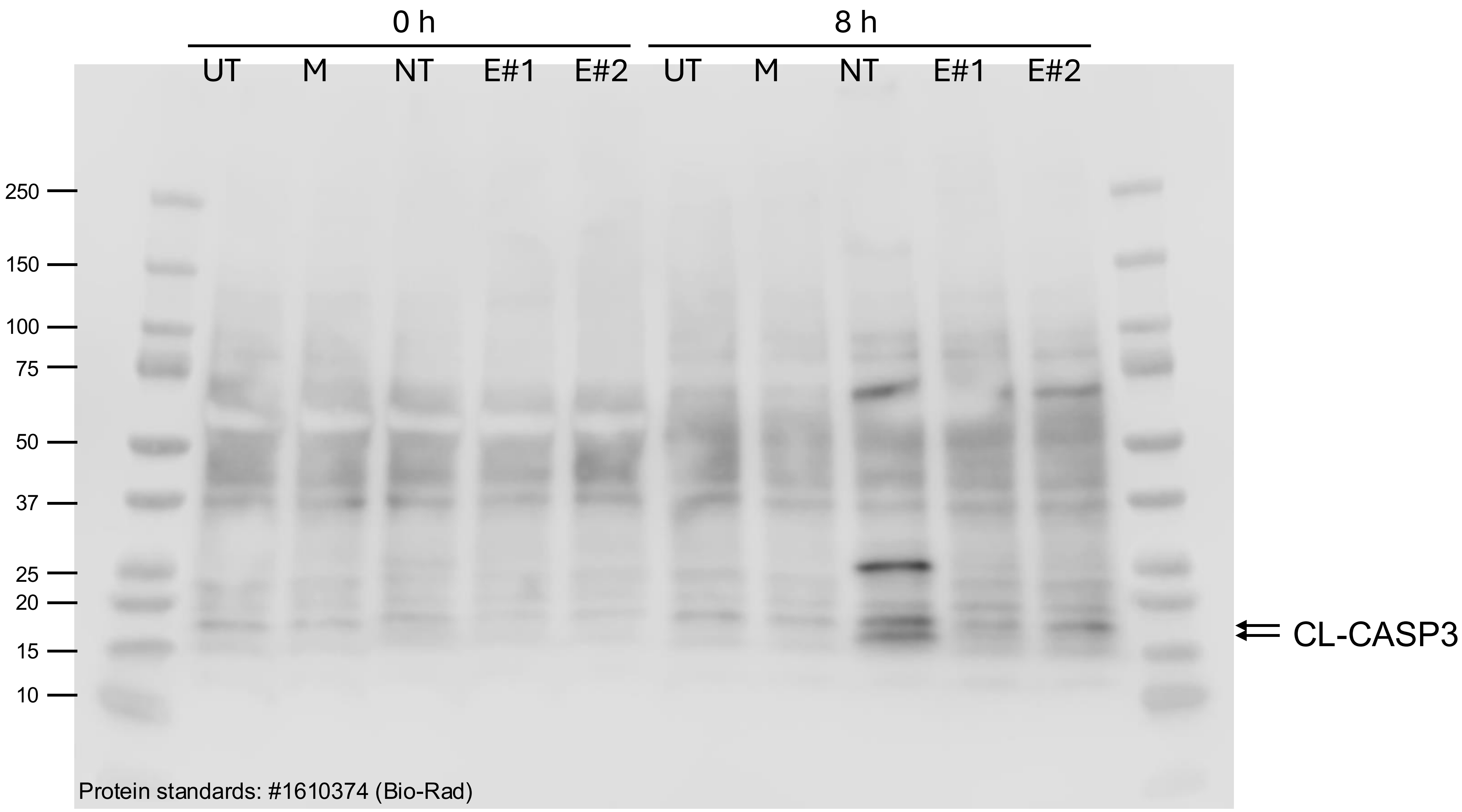

Original western blot: Fig S8c

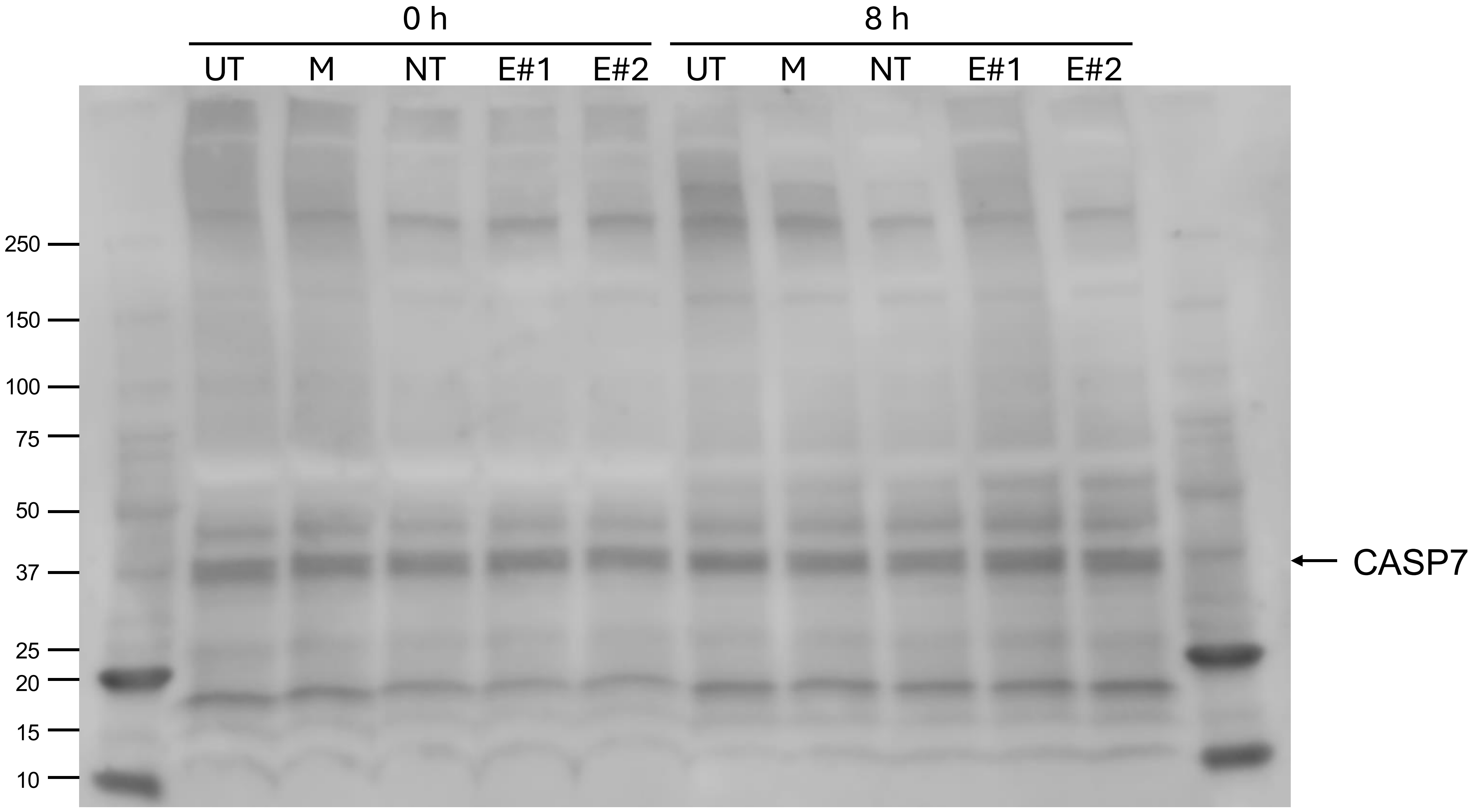

Protein standards: #1610374 (Bio-Rad)

Original western blot: Fig S8c

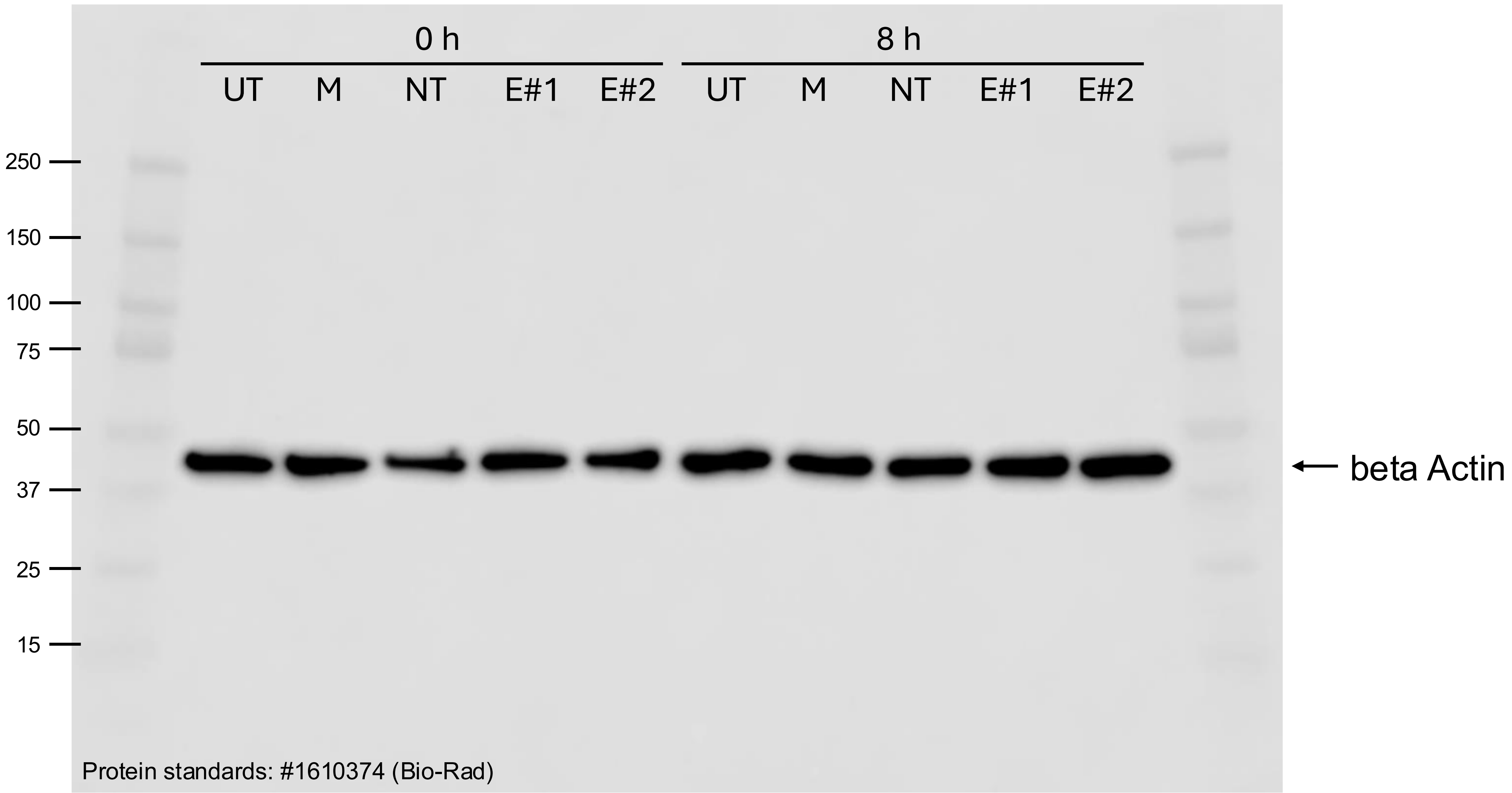

Supplement: Supplementary file 14 — Western Blot original images [file 41419_2025_8341_MOESM14_ESM.pdf]
